# Targeting focal adhesion kinase overcomes erlotinib resistance in smoke induced lung cancer by altering phosphorylation of epidermal growth factor receptor

**DOI:** 10.18632/oncoscience.395

**Published:** 2018-02-23

**Authors:** Hitendra S. Solanki, Remya Raja, Alex Zhavoronkov, Ivan V. Ozerov, Artem V. Artemov, Jayshree Advani, Aneesha Radhakrishnan, Niraj Babu, Vinuth N. Puttamallesh, Nazia Syed, Vishalakshi Nanjappa, Tejaswini Subbannayya, Nandini A. Sahasrabuddhe, Arun H. Patil, T.S. Keshava Prasad, Daria Gaykalova, Xiaofei Chang, Rachana Sathyendran, Premendu Prakash Mathur, Annapoorni Rangarajan, David Sidransky, Akhilesh Pandey, Evgeny Izumchenko, Harsha Gowda, Aditi Chatterjee

**Affiliations:** ^1^ Institute of Bioinformatics, International Tech Park, Bangalore 560066, India; ^2^ School of Biotechnology, Kalinga Institute of Industrial Technology, Bhubaneswar, Odisha 751024, India; ^3^ Insilico Medicine, Inc., Emerging Technology Centers, Johns Hopkins University at Eastern, Baltimore, MD 21218, USA; ^4^ Manipal Academy of Higher Education, Manipal, Karnataka 576104, India; ^5^ School of Biotechnology, Amrita University, Kollam 690525, India; ^6^ Center for Systems Biology and Molecular Medicine, Yenepoya (Deemed to be University), Mangalore 575018, India; ^7^ NIMHANS-IOB Proteomics and Bioinformatics Laboratory, Neurobiology Research Centre, National Institute of Mental Health and Neurosciences, Bangalore 560029, India; ^8^ Department of Otolaryngology-Head and Neck Surgery, Johns Hopkins University School of Medicine, Baltimore, MD 21231, USA; ^9^ Department of Molecular Reproduction, Development and Genetics, Indian Institute of Science, Bangalore, 560012, India; ^10^ McKusick-Nathans Institute of Genetic Medicine, Johns Hopkins University School of Medicine, Baltimore, MD 21205, USA; ^11^ Department of Biological Chemistry, Johns Hopkins University School of Medicine, Baltimore, MD 21205, USA; ^12^ Department of Oncology, Johns Hopkins University School of Medicine, Baltimore, MD 21205, USA; ^13^ Department of Pathology, Johns Hopkins University School of Medicine, Baltimore, MD 21205, USA

**Keywords:** cigarette smoke, epidermal growth factor receptor, NSCLC, phosphoproteomics, drug resistance

## Abstract

EGFR-based targeted therapies have shown limited success in smokers. Identification of alternate signaling mechanism(s) leading to TKI resistance in smokers is critically important. We observed increased resistance to erlotinib in H358 NSCLC (non-small cell lung carcinoma) cells chronically exposed to cigarette smoke (H358-S) compared to parental cells. SILAC-based mass-spectrometry approach was used to study altered signaling in H358-S cell line. Importantly, among the top phosphosites in H358-S cells we observed hyperphosphorylation of EGFR (Y1197) and non-receptor tyrosine kinase FAK (Y576/577). Supporting these observations, a transcriptomic-based pathway activation analysis of TCGA NSCLC datasets revealed that FAK and EGFR internalization pathways were significantly upregulated in smoking patients, compared to the never-smokers and were associated with elevated PI3K signaling and lower level of caspase cascade and E-cadherin pathways activation. We show that inhibition of FAK led to decreased cellular proliferation and invasive ability of the smoke-exposed cells, and restored their dependency on EGFR signaling. Our data suggests that activation of focal adhesion pathway significantly contributes to erlotinib resistance, and that FAK is a potential therapeutic target for management of erlotinib resistance in smoke-induced NSCLC.

## INTRODUCTION

Smoking is the leading cause of lung cancer, which attributes to 87% of lung cancer deaths in males and 70% of lung cancer deaths in females in the United States (Cancer Facts & Figures 2014). Non-small cell lung carcinoma (NSCLC) accounts for 85% of lung cancer cases, which is diagnosed primarily in current or former smokers [1]. NSCLC is usually diagnosed at an advanced stage owing to the late onset of clinical symptoms and inadequate screening programs. It is established that tyrosine kinases serve as excellent therapeutic targets [2]. Currently, the first line of treatment for NSCLC are EGFR inhibitors (erlotinib, gefitinib) which are known to benefit patients harboring specific activating somatic mutations (exon 19 deletion or exon 21 substitution (L585R) or exon 18 substitution (G719C, G719S, G719A)) in the kinase domain of EGFR [3]. Acquired drug resistance eventually arises in most, if not all, treated patients [4] and secondary mutations in EGFR (T790M), c-met or Her2 amplification are known to be associated with acquired drug resistance [5]. Among the NSCLC patients harboring EGFR mutations who underwent tyrosine kinase inhibitor (TKI) treatment, non-smokers have shown longer progression free survival response than ever smokers [6, 7]. Several *in vitro* studies have shown that acute exposure to cigarette smoke mediates development of lung cancer and resistance to TKIs in NSCLC in both wild type (WT) EGFR and TKI sensitive mutants [8-11]. However, underlying mechanism(s) leading to erlotinib resistance upon cigarette smoke exposure in NSCLC is not well understood. This preempts the need to investigate the underlying signaling pathways contributing to resistance to EGFR-targeted TKIs in NSCLC.

Mass spectrometry based-phosphoproteome profiling is widely used to identify alterations in signaling and to identify novel therapeutic targets in cancer [12-14]. We have shown previously that chronic exposure to cigarette smoke induces distinct molecular signatures in lung cancer cell line exposed to cigarette smoke [15]. In this study, we show that chronic exposure to cigarette smoke renders resistant to erlotinib in lung cancer cells. We carried out SILAC-based quantitative mass spectrometry analysis to identify aberrantly activated signaling pathways in lung cancer cells chronically exposed to cigarette smoke. We identified 238 phosphosites (or phosphopeptides) corresponding to 157 proteins of which 111 phosphosites were hyperphosphorylated while 66 were hypophosphorylated (2.0 -fold) in H358-S cells compared to parental cells. We observed hyperphosphorylation of key signaling molecules including EGFR (Y1197) (corresponds to the Y1173 of mature EGFR), focal adhesion kinase 1 (FAK or PTK2) (Y576/577) and Fyn related Src family tyrosine kinase (FRK or RAK) (Y46) amongst others. We identified differential phosphorylation status of EGFR in H358-S cells which directly correlated with erlotinib resistance. Using iPANDA, a bioinformatics software suite for qualitative analysis of intracellular signaling pathway activation based on transcriptomic data [16, 17], we revealed that FAK signaling and EGFR internalization pathway were significantly upregulated in smoking patients from TCGA NSCLC dataset, compared to the never-smoker counterparts. We further report that FAK signaling regulates EGFR phosphorylation in H358 smoke exposed cells and NSCLC cells derived from smokers independent of SRC. Our study underscores the importance of FAK pathway in regulating EGFR activity in NSCLC and could be an effective therapeutic strategy for NSCLC patients with smoking habits.

## RESULTS

### Chronic exposure to cigarette smoke enhanced tumorigenicity and erlotinib resistance in NSCLC

**Figure 1:**
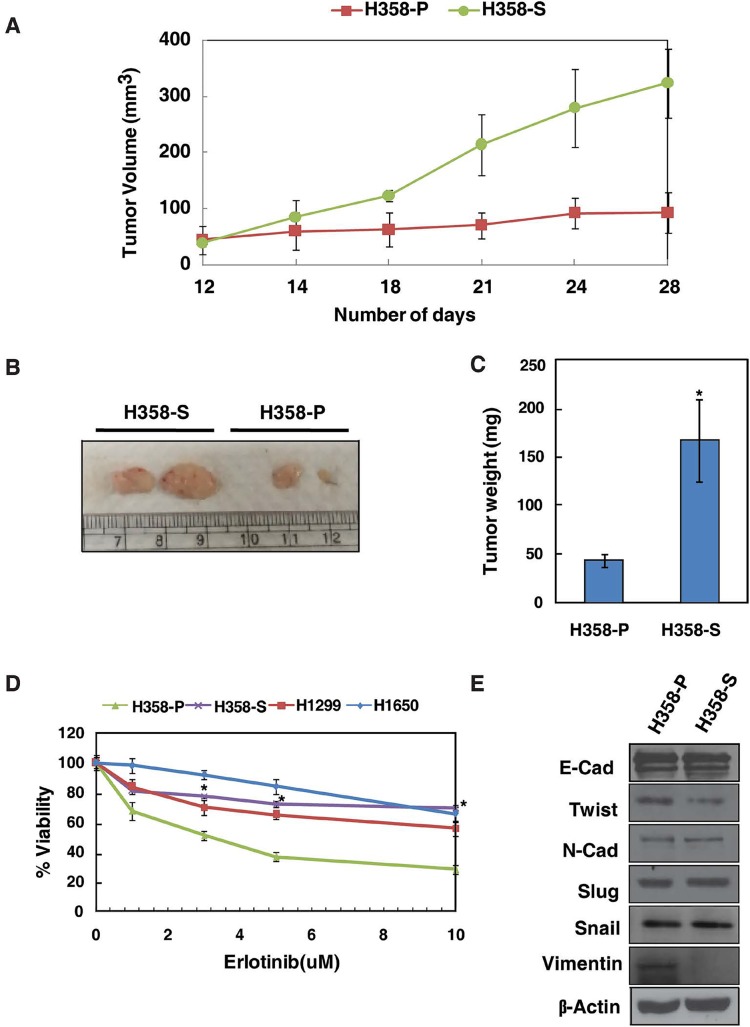
Chronic exposure to cigarette smoke enhanced tumorigenicity and erlotinib resistance in NSCLC (A) H358-P and H358-S (2×10^6^) cells were injected subcutaneously into the flanks of male NOD-SCID mice. The growth kinetics over a period of 3 weeks has been plotted. Representative pictures (B) and bar graph representing the tumor weights (C) are shown. (D) Cellular sensitivity of H358-P, H358-S, H1299 and H1650 cells treated with indicated concentrations of erlotinib. Experiments were performed in triplicates. *p < 0.05. (E) Western blot analysis of epithelial and mesenchymal markers in H358-P and H358-S cells. β-actin served as a loading control.

In our earlier studies we have shown that chronic exposure to smoke increased the proliferative and invasive abilities of lung cancer H358 cells [15]. The untreated cells and smoke-exposed cells were designated as H358-P and H358-S, respectively. In this study, we further reaffirmed the enhanced tumorigenic capacity of H358-S cells using an *in vivo* mice model. Xenograft studies indicated that mice bearing H358-S tumors showed increased growth kinetics compared to H358-P group (Figure 1A-C). H358 cells have been reported to be sensitive to erlotinib [18]. We next determined the chronic effects of cigarette smoke exposure to erlotinib sensitivity of H358-S and other NSCLC cells derived from smokers (H1299 (WT-EGFR) and H1650 (Exon 19 deletion)). As shown in Figure 1D, the H358-S cells acquired resistance to erlotinib (IC_50_ > 10 µM) compared to H358-P. The acquired resistance of H358-S cells were at par with, H1299 and H1650 NSCLC cell lines which are known to be resistant to erlotinib (IC_50_ > 10 µM).

### Chronic cigarette smoke exposure does not induce epithelial-mesenchymal transition in H358-S cells

Previous studies have shown that acute exposure to cigarette smoke induces epithelial to mesenchymal transition (EMT) transition in lung epithelial cells [19]. In addition, studies have shown a close link between cigarette smoke induced EMT and acquired resistance to EGFR-TKI in NSCLC [20]. To confirm whether chronic exposure to smoke induce EMT in H358-S cells, we checked the expression of epithelial and mesenchymal markers in H358-P and H358-S cells. As shown in Figure 1E, E-cadherin expression remained unchanged and we observed a decrease in expression of vimentin between H358-P and H358-S cells. Other mesenchymal markers remained unchanged between H358-P and H358-S cells. Studies in hepatocellular carcinoma and ovarian carcinoma indicate enhanced migratory and invasive properties of cells independent of EMT [21, 22]. Our data also indicates that the increased resistance to erlotinib in H358-S cells is independent of EMT transition, suggesting the existence of alternative mechanism(s) for erlotinib resistance in lung cancer cells upon chronic exposure to cigarette smoke.

### Chronic exposure to cigarette smoke induces altered signaling in lung cells

Acquired resistance to EGFR-TKI therapy has been reported in both WT-EGFR and EGFR sensitive mutants subgroup among smokers [6, 8]. Moreover, TKI-responsive patients often develop resistance to these drugs if they resume smoking post-treatment [23]. Studies also indicate that smokers acquire distinct EGFR mutations [24] and that exposure to smoke leads to aberrant phosphorylation of EGFR that confers TKI-resistance in smokers [25]. Consistently, our results show that H358 cells acquired resistance to erlotinib after chronic exposure to cigarette smoke. We carried out SILAC-based quantitative proteomic approach to systematically interrogate altered signaling pathways that could potentially contribute to erlotinib resistance in H358-S cells. The workflow for phosphotyrosine analysis is depicted in Supplementary Figure 1. Mass spectrometry based analysis led to the identification of 238 phosphosites corresponding to 157 proteins of which 111 phosphosites were hyperphosphorylated and 66 were hypophosphorylated (≥ 2-fold) in H358-S cells. The partial list of hyperphosphorylated sites is shown in Table 1. The complete list of identified phosphopeptides is provided in Supplementary Table 1.

**Table 1:** Partial list of hyperphosphorylated proteins upon cigarette smoke exposure in H358 cells

	Protein	Gene Symbol	Phosphopeptide	Site	CSC/parental (fold change)
1.	Mitogen-activated protein kinase 14	*MAPK14*	HTDDEMTG[Y]VATR	Y182	6.8
2.	Mitogen-activated protein kinase 3	*MAPK3*	IADPEHDHTGFLTE[Y]VATR	Y204	3.2
3.	Paxillin	*PXN*	VGEEEHV[Y]SFPNKQK	Y118	2.8
4.	Focal adhesion kinase 1	*PTK2*	YMEDST[Y]YKASK	Y576	3.1
5.	Epidermal growth factor receptor	*EGFR*	EAKPNGIFKGSTAENAE[Y]LR	Y1197	2.6
6.	Wiskott-Aldrich syndrome-like	*WASL*	DRETSKVI[Y]DFIEK	Y256	2.5
7.	Cortactin	*CTTN*	GPVSGTEPEPVYSMEAAD[Y]R	Y453	2.1

**Figure 2:**
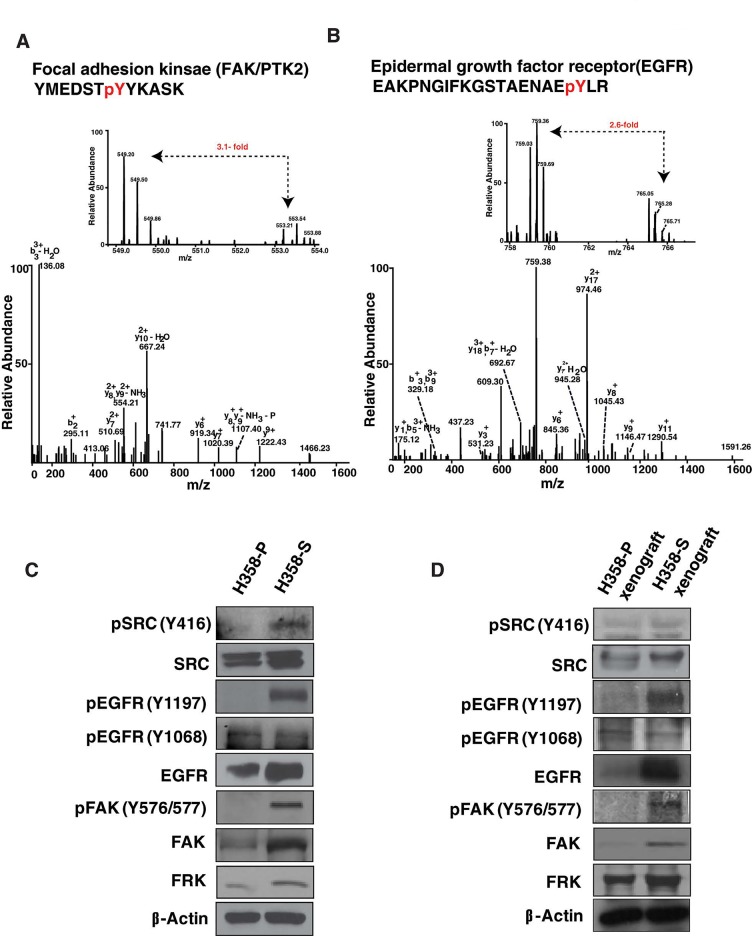
Representative MS and MS/MS spectra of hyperphosphorylated peptide of in H358 smoke-exposed cells (A) Focal adhesion kinase (Y576/577) (B) Epidermal growth factor receptor (Y1197) (C) Western blot analysis of p-SRC, p-EGFR, p-FAK and FRK in H358-P and H358-S cells (D) Western blot analysis of p-SRC, p-EGFR, p-FAK and FRK between H358-S xenograft tissue and H358-P xenograft tissue.

We identified hyperphosphorylation of receptor tyrosine kinases including EGFR at Y1197 (2.6-fold) and ephrin type-A receptor 1 (EPHA1) at Y781 (10.8-fold). Phosphorylation of EGFR at Y1197 leads to internalization of the receptor [26, 27]. The ephrin receptor is up-regulated in many cancer types including NSCLC, specifically in smokers [28-30]. In addition, we also observed hyperphosphorylation of non-receptor tyrosine kinases including PTK2/FAK and FRK/RAK at Y576/577 (3.1-fold) and Y46 (2.1-fold), respectively. Further, our data indicates hyperphosphorylation of several key members of SRC and FAK signaling cascade including paxillin (PXN) (2.8-fold), WASL (2.5-fold), CTTN (2.1-fold), CAV1 (2.0-fold) and Talin-1 (TLN1) (3.5-fold). These findings suggest widespread perturbation of tyrosine phosphorylation-regulated signaling in response to cigarette smoke in lung cancer cells. The MS and MS/MS spectra of hyperphosphorylated phosphopeptides of FAK (Y576/577) and EGFR (Y1197) are depicted in Figure 2 (A-B); MS and MS/MS spectra for FRK (Y46) is depicted in Supplementary Figure 2.

### Cigarette smoke induces aberrant EGFR phosphorylation in H358 lung cancer cells

Studies have reported that acute exposure of cigarette smoke induced altered phosphorylation of EGFR in lung cells [25]. We determined the phosphorylation status of EGFR at Y1197 in H358-S cell line. In concordance with our mass spectrometry data, Western blot analysis confirmed hyperphosphorylation of EGFR at its auto-phosphorylation site Y1197 in H358-S cells (Figure 2C). Phosphorylation at Y1197 is known to contribute to EGFR internalization [26, 27]. The phosphorylated form of EGFR, specifically at Y1068 is reported to be predictive marker to screen TKI-responsive population in NSCLC with wild-type EGFR [31]. Gowda *et al*., have shown that phosphorylation of EGFR at Y1068 can be a predictive marker of erlotinib sensitivity and used to screen TKIs sensitive pancreatic cancer patients [32]. Though we did not identify EGFR Y1068 in our mass spectrometry data, we determined the phosphorylation status of EGFR at Y1068 in H358-S cells. In concordance with previous published studies, we observed a loss of phosphorylation of EGFR at Y1068 in erlotinib resistant H358-S cells compared to the parental cells (Figure 2C). We also observed increased phosphorylation of SRC (Y416) and FAK (Y576/577) in H358-S cells. SRC is known to be phosphorylated and activated upon acute exposure of cigarette smoke or when treated with NNK-(Nitrosamine 4-(methylnitrosamino)-1-(3-pyridyl)-1-butanone), an important carcinogen present in cigarette smoke [8, 25]. Also, SRC plays a key role in phosphorylation of FAK at Y576 and Y577, leading to its activation [33, 34]. In addition, we identified hyperphosphorylation of FRK/RAK (Y46) (2.1-fold) in H358-S cells. FRK/RAK belongs to Src family of kinases and is reported to phosphorylate EGFR at Y1197, resulting in receptor internalization [35]. Due to unavailability of specific phospho-antibody against FRK, we studied the expression levels of FRK and our data shows increased expression of FRK in the H358-S cells. Overexpression and hyperphosphorylation of FRK is reported in many cancer types, including lung cancer [35, 36]. Our findings indicate an altered phosphorylation state of EGFR with concomitant activation of SRC/FAK signaling in erlotinib-resistant H358-S cells. In agreement with the *in vitro* data, H358-S xenograft tissue also showed increased phosphorylation of EGFR at Y1197 and a decreased phosphorylation at Y1068. Similarly, we observed hyperphosphorylation of SRC, FAK and an increased expression of FRK in the H358-S xenograft tissue compared to H358-P tumors (Figure 2D). Taken together, our data strongly suggests that treatment with cigarette smoke results in aberrant phosphorylation of EGFR with concomitant activation of SRC/FAK signaling and could be one of the factors contributing to erlotinib resistance.

### Cigarette smoke exposure correlates with upregulation of FAK signaling pathway

We first performed pathways enrichment analyses with either Search Tool for Interactions of Chemicals (STITCH) and Ingenuity Pathway Analysis (IPA) bioinformatic toolsets, using the differentially regulated kinases (from the phospho proteomic data) in H358-S cells as an input. Interestingly, analysis with STITCH revealed that focal adhesion pathway was among the top 5 signaling axes enriched in H358-S cells (Supplementary Table 2A). In addition, IPA revealed the enrichment of integrin and paxillin signaling cascade, where the activation of focal adhesion kinase is critically important (the list of top 5 canonical signaling cascades identified in IPA analysis is provided in Supplementary Table 2B). Although the direct comparison of the enrichment analyses generated by different approaches should be addressed with caution, this data further supports our observation that chronic exposure to cigarette smoke may activate the focal adhesion pathways to support proliferation and survival.

**Figure 3:**
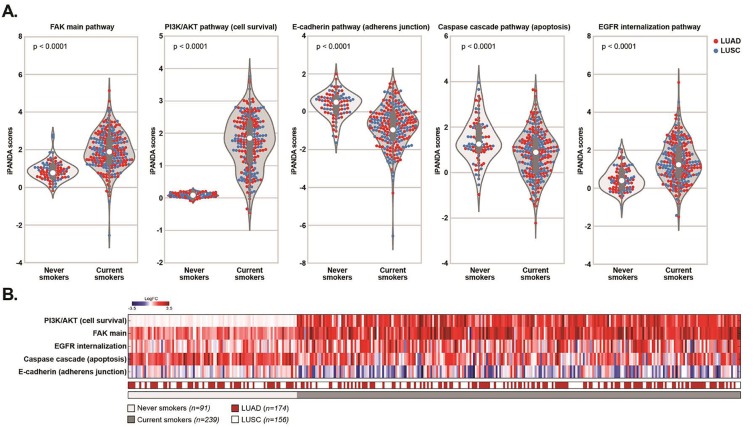
Cigarette smoke exposure correlates with upregulation of FAK signaling pathway in TCGA NSCLC dataset iPANDA software suite for analysis of intracellular signaling pathway activation based on transcriptomic data was used to estimate the level of the indicated signaling pathways in never-smokers (n=91) and currently smoking (n=239) patients with NSCLC from TCGA-LUAD (lung adenocarcinoma) and TCGA-LUSC (lung squamous cell carcinoma) datasets. TCGA transcriptomic data from normal lung of never-smoking individuals was used as a reference after proper normalization. (A) Violin plots showing the differences in pathway activation scores between the never-smoker and current-smoker patients. In violin plots: red dots represent LUAD patients, blue dots represent LUSC patients, the white dot represents the median value and the gray bar is the interquartile range. (B) The heatmap of indicated differentially activated pathways in all samples analyses. Downregulated iPANDA values for each sample/pathway are indicated in blue, whilst upregulated values are shaded in red.

We next used iPANDA, a bioinformatics software suite for qualitative analysis of intracellular signaling pathway activation based on transcriptomic data [16, 17], to assess the level of FAK pathway activation in current smokers (n=239) or never-smoker (n=91) patients with NSCLC (both adenocarcinoma and squamous cell carcinoma) derived from The Cancer Genome Atlas database. The transcriptomic data from noncancerous lung samples of never-smoking individuals (n=7) was used as a reference. Signaling events mediated by focal adhesion kinase were derived from the Pathway Interaction Database (PID) curated by NCI/Nature. Consistent with our cell line results, main FAK pathway activation was significantly upregulated in smoking patients compared to the never smokers (Figure 3A, 3B). In various solid malignancies including lung, FAK activation induces survival, migration and invasion signaling by promoting E-cadherin delocalization [37-39], upregulation of PI3K/AKT signaling [40, 41] and suppression of apoptosis [42]. Analysis of these signaling axes retrieved from the PID collection revealed that PI3K/AKT pathway was significantly upregulated in the current smoker cohort, whereas E cadherin signaling and caspase cascade apoptosis pathway were downregulated in this group of patients compared to the never-smokers (Figure 3A, 3B). Since cigarette smoke induced EGFR phosphorylation at Y1197 (Figure 2B-D) is associated with elevated EGFR internalization rate [43, 44], decreased sensitivity to EGFR inhibitors [45, 46] and shorter progression-free survival [47], we also assessed the levels of the EGFR internalization pathway activation in the same patient's cohorts. Notably, activation of the main EGFR internalization pathway was significantly higher in smoking patients compared to the never-smoking counterparts (Figure 3). These observations further emphasize the important role of cigarette smoke induced dysregulation of FAK signaling in lung tumorigenesis and resistance to EGFR targeted therapies.

### Inhibition of FAK and SRC decreases the invasive property of lung cancer cells

**Figure 4:**
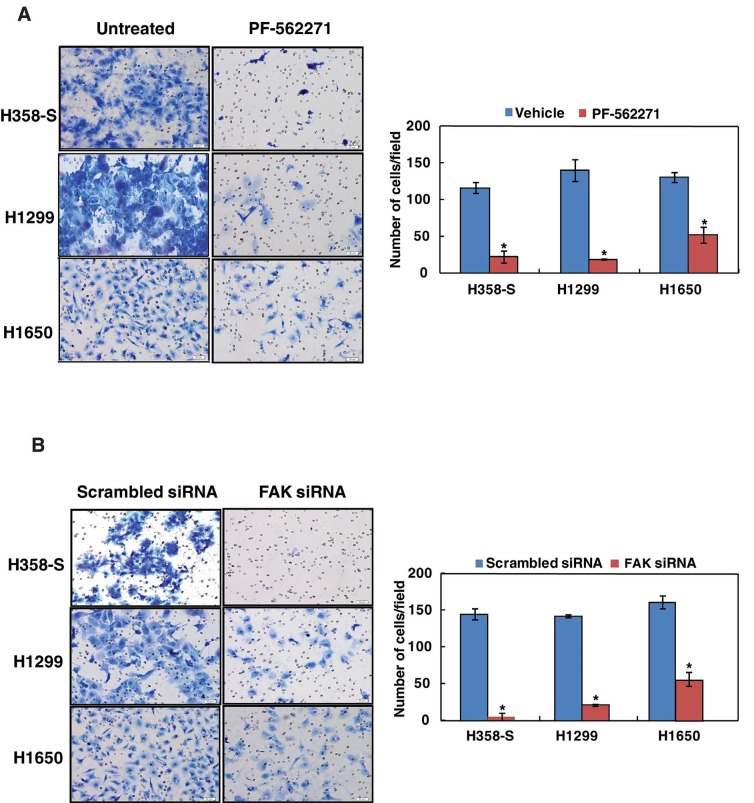
Inhibition of FAK decreases the invasive property of lung cancer cells Invasion assays were carried out in a transwell system using Matrigel-coated filters and the number of cells that migrated to the lower chamber was counted. Cells that migrated are visualized following methylene blue staining in H358-S and NSCLC cell lines, H1299 and H1650 as indicated. (A) Cells were treated with either DMSO (vehicle control) or PF-562271 and invaded cells were photographed. Representative images were photographed at a magnification 10x. Invaded cells were counted and relative changes in invasive ability of H358-S and NSCLC cells upon inhibition with PF-562271 was calculated and represented graphically (*p<0.05). (B) Cells were transfected with either scrambled siRNA or FAK siRNA and invaded cells were photographed. Invaded cells were counted and relative changes in invasive ability of H358-S and NSCLC cells upon FAK silencing was calculated and represented graphically (*p<0.05). Representative images were photographed at a magnification 10x.

Since both FAK and SRC were found to be activated in lung cancer cells chronically exposed to cigarette smoke, we assessed the potential role of FAK and SRC in regulating invasive potential in H358-S and a panel of NSCLC cells established from smokers. We observed a significant decrease in the invasive ability of the H538-S and other smoke-exposed NSCLC cells upon FAK inhibition/silencing using PF-562271 or FAK siRNA (Figure 4A-B). A similar decrease in the invasive property of these cells was observed when cells were treated with SRC inhibitor dasatinib (Supplementary Figure 3A).

### Targeting FAK overcomes erlotinib resistance in lung cancer cells

**Figure 5:**
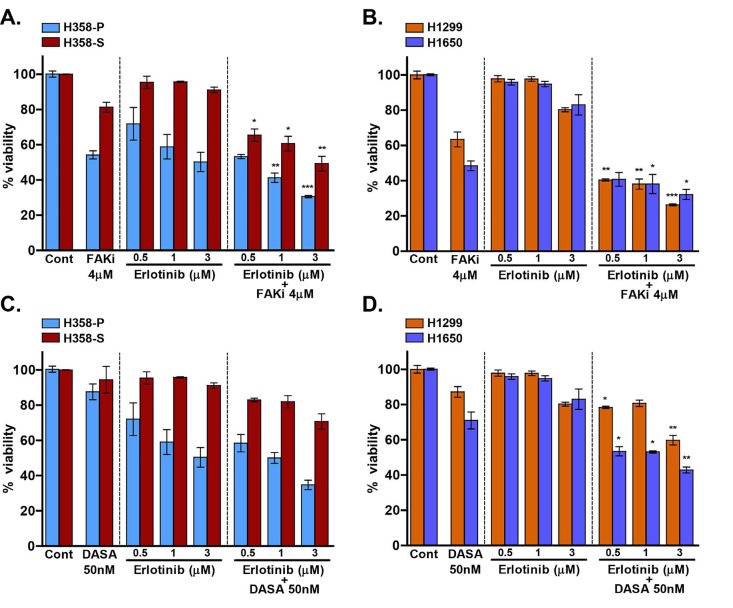
Targeting FAK overcomes erlotinib resistance in lung cancer cells H358-S cells were treated with FAK inhibitor (FAKi) PF 562271 (A) or dasatinib (DASA) (C) alone or in combination with varying concentrations of erlotinib (0.5 µM, 1 µM and 3 µM). NSCLC cells, H1299 and H1650 were treated with PF 562271 (B) or with dasatinib (D) alone or in combination with the indicated concentrations of erlotinib. Cell viability was assessed using MTT assay. The data is presented as mean of % viability ± SEM as compared to DMSO-treated cells which served as control. The significance of combination therapies was assessed relative to most effective single agent treatment (* p < 0.05, ** p < 0.01, *** p < 0.001).

Having observed that the inhibition of SRC/FAK signaling in H358-S and other NSCLC cells leads to decreased invasive ability, we sought to study the effects of these inhibitors on erlotinib sensitivity of smoke-exposed cells. In a recent study, combinatorial treatment of FAK inhibitor and erlotinib have shown synergistic effect in reducing cell viability in EGFR-TKI-resistant NSCLC cell lines [48]. H358-S, H1299 and H1650 cells were treated with varying concentration of erlotinib (0.5 µM, 1 µM and 3 µM) alone or in combination with either SRC inhibitor, dasatanib (50 nM) or FAK inhibitor, PF-562271 (4 µM) (Figure 5A-D). Combined treatment with PF-562271 (FAK inhibitor), significantly improved the sensitivity of H358-S cells to erlotinib (Figure 5A). Similarly, H1299 and H1650 cells also exhibited an increased sensitivity to erlotinib when treated in combination with FAK inhibitor (Figure 5B). Study by Gold *et al*., has reported synergistic effect of erlotinib and dasatinib in NSCLC [49] and another study has independently reported that the treatment with dasatinib can overcome cigarette smoke induced TKI resistance in NSCLC cells [8]. As FAK is a known downstream target of SRC, we next determined whether targeting SRC can have similar effect as targeting FAK. In contrast to the published results, when H358-S cells were treated with a combination of dasatinib and erlotinib, we did not observe any significant increase in the cellular response compared to the single agent therapy (Figure 5C). While the additive effect of dasatinib/erlotinib combination on survival of H1299 and H1650 cells was significant, it was not as egregious as one seen with erlotinib/PF-562271 combination (Figure 5D). Of note, a better response to the dasatinib/erlotinib therapy seen in the H1650 cells can be attributed to the fact that H1650 cell line is known to be sensitive to dasatinib [50]. Collectively, our data indicates that inhibition of FAK with PF-562271 in combination with erlotinib can effectively reduce the cell viability of NSCLC, specifically in smokers who develops resistance to EGFR-TKI therapy.

### FAK inhibition alters smoke induced EGFR phosphorylation in lung cancer cells

**Figure 6:**
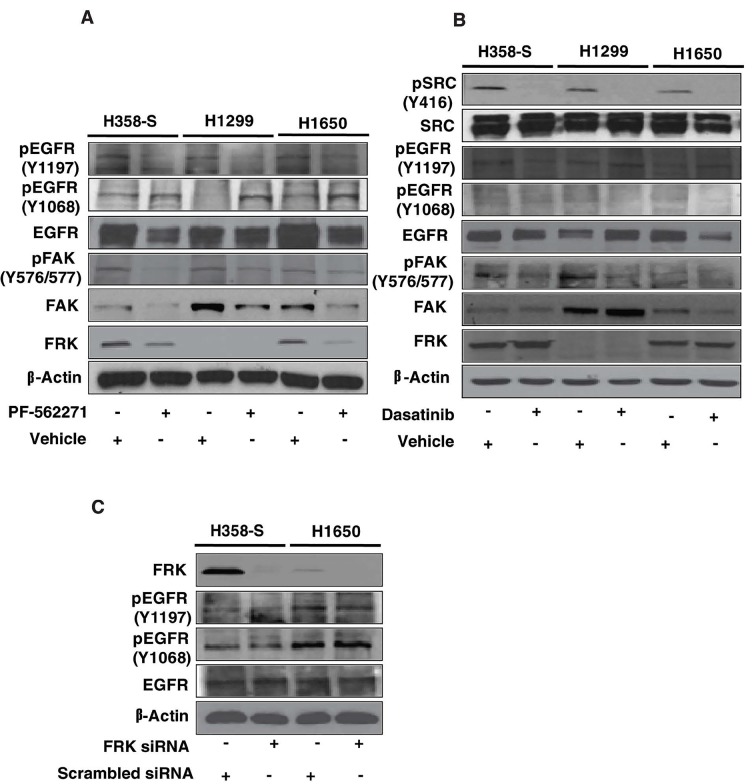
Western blot analysis depicting phosphorylation status of EGFR upon FAK and SRC inhibition in H358-S and in a panel of smoker-derived cell lines (H1299 and H1650) (A) The relative phosphorylation levels of in FAK (Y 576/577), EGFR (Y1197, Y1068) and FRK expression in H358-S, H1299 and H1650 cells treated either with DMSO (vehicle control) or dasatinib (50 nM). (B) The relative expression of phosphorylated SRC (Y416), FAK (Y 576/577), EGFR (Y1197, Y1068) and FRK expression in H358-S, H1299 and H1650 cells treated either with DMSO (vehicle control) and PF 562271 (4 µM). (C) H358-S and H1650 were transfected using scrambled or FRK siRNA and western blot analysis was performed for the indicated proteins. β-actin served as a loading control under all conditions.

Our results indicate that erlotinib resistance in NSCLC can be overcome by complementing the EGFR-TKI therapy with FAK inhibitors and not with SRC inhibition. Though FAK is reported to be downstream of SRC in literature, at this juncture it was unclear to us why SRC inhibition did not restore the sensitivity of the cells to erlotinib, while inhibition of FAK rendered the cells sensitive to erlotinib [37]. To understand this phenomenon, we studied the signaling mechanism in H358-S cells using FAK and SRC inhibitors, PF-562271 and dasatinib respectively. We first studied the effect of FAK inhibition on EGFR phosphorylation in H358-S cells. We had observed a decreased phosphorylation of EGFR at Y1068 and hyperphophorylation at EGFR Y1197 in H358-S cells compared to the parental cell-line. Upon inhibition of FAK, phosphorylation of EGFR at Y1068 was restored in H358-S cells whereas there was a loss of phosphorylation at EGFR Y1197 (Figure 6A). A similar response was also observed in the panel of smoker-derived NSCLC cell lines (H1299 and H1650). Next, we studied the effect of SRC inhibitor dasatinib on EGFR phosphorylation. SRC inhibition had little to no effect on the phosphorylation of EGFR at Y1197, and did not reinstate phosphorylation of EGFR at Y1068 (Figure 6B). Interestingly, we observed a decrease in FRK levels in presence of PF-562271 but not dasatinib (Figure 6A-B, FRK panel); indicating that FRK might function downstream to FAK signaling in smoke-exposed cells.

To understand the possible role of FRK in erlotinib resistance, we studied the effect of silencing FRK on cellular invasion. Supplementary Figure 3B shows that siRNA-mediated silencing of FRK led to decreased cellular invasion of the H358-S and other NSCLC cells. Next, we studied the effects of FRK knockdown on EGFR phosphorylation levels. We did not observe any significant change in the phosphorylation status of EGFR at both Y1197 and Y1068 upon silencing of FRK (Figure 6C). In addition, silencing of FRK in H358-S and NSCLC cells did not alter the sensitivity of cells to erlotinib (data not shown). Due to the lack of FRK phospho-antibody and specific inhibitors we could not delineate the role of FRK in mediating erlotinib resistance upon cigarette smoke exposure, and future studies are warranted to further evaluate its role in smoke-induces erlotinib resistance. Taken together our results indicate that inhibition of FAK increases/restores cellular sensitivity to erlotinib by activating EGFR, which is independent of SRC.

## DISCUSSION

TKIs (erlotinib, gefitinib and afatinib) are in clinical practice to treat patients harboring specific activating somatic mutations in EGFR (exon 19 deletion or exon 21 substitution (L585R) or exon 18 substitutions (G719C, G719S, G719A) [51]. However, patients with WT-EGFR have shown poor response to TKIs [52]. Patients with smoking habits are reported to develop resistance towards TKIs in both WT-EGFR and TKIs-sensitive EGFR mutants [6, 8]. There are limited reports which explain the mechanisms associated with acquired resistance to TKIs upon acute exposure of cigarette smoke [8, 25]. Filosto *et al*., have shown that acute exposure to cigarette smoke leads to the activation of SRC which in turn binds to EGFR and induce conformation change in ATP binding site of receptor [8]. Erlotinib is known to reversibly bind to the ATP binding site which interrupts further signaling cascade [53]. Along with SRC, CYP1A1 and Acetylcholine receptor (nAChR) were also suggested as potential targets to overcome smoking/nicotine-based acquired resistance towards TKIs [54, 55]. Long term exposure to cigarette smoke is known to induce carboplatin resistance in NSCLC through down-regulation of SMAD family member 3 (SMAD3) [56]. In this study, using an *in vitro* cellular model that mimics long term cigarette smoking in NSCLC, we demonstrate that chronic exposure to cigarette smoke renders resistance to erlotinib.

To systematically characterize the signaling pathways leading to erlotinib resistance due to cigarette smoke exposure, we studied the phosphoproteomic alterations in H358-S and H358-P cells. In our data, we identified aberrant phosphorylation of receptor tyrosine kinases EGFR (Y1197), EPHA1 (Y781) and non-receptor tyrosine kinases including FAK (Y576/577) and FRK (Y46) in H358-S cells. EPHA2 expression is reported to be up-regulated in NSCLC, specifically in smokers [30] and has been shown to regulate acquired resistance towards TKIs [57]. A recent study using multiple reaction monitoring (MRM) based proteomics has shown increased phosphorylation of FAK1 amongst other proteins in erlotinib-resistant cells [58]. Other molecules which were found to be hyperphosphorylated in H358-S cells includes MAPK14, MAPK3, PXN, WASL, CTTN, CAV1 and TLN1, many of which are known to play a key role in SRC and FAK signaling. PXN is the known substrate of FAK [59] and plays critical role in tumorigenesis by regulating cytoskeleton arrangement by connecting integrins to FAK [60]. Studies indicate that MAPK3, CTTN and PXN regulate chemo or TKIs-based targeted therapy in cancer. TLN1 is known to activate integrins which in turn prevent cells from anoikis and promote metastasis via AKT pathway [61]. CTTN is a known substrate of SRC and is reported to induce gefitinib resistance in head and neck squamous cell carcinoma (HNSCC) [61]. FRK (related to Src family of kinases) is known to be aberrantly phosphorylated in NSCLC and there are reports which indicate its dual role as tumor suppressor or promoter depending on cell type [35]. Hyperphosphorylation at MAPK3 is reported to induce cisplatin resistance in lung cancer cells [62]. Yoshida *et al*., have reported activation of Src-family kinase network and SRC signaling was shown to work independent of EGFR in TKI-resistant NSCLC cells [63]. Several other studies have shown that smoke-induced EMT contributed to erlotinib resistance [20, 64, 65]. However, we did not find mesenchymal switch upon chronic exposure to cigarette smoke in our cell line model.

FAK is upregulated and differentially phosphorylated in multiple cancers including breast cancer and NSCLC [66-68]. We identified hyperphosphorylation of FAK at Y576/577 upon chronic exposure to cigarette smoke in lung cancer cells. Emerging studies have shown FAK as potential cancer target in multiple cancer types [69-71] and phase I/II clinical trial outcomes of FAK inhibitors are awaited [72]. A recent study reported higher efficacy and synergistic effects of FAK inhibitor when used in combination with erlotinib using *in-vitro* and *in-vivo* experiments [48]. The SRC-3Delta4, a SRC3 splicing isoform is known to mediate the interaction of FAK with EGFR in mammals [73]. FAK is reported to negatively regulate RTK-MAPK signaling by reducing the recycling of EGFR on plasma membrane [74]. The internalized receptor is reported to retain phosphorylation at Y1197 and localize in perinuclear region and nucleus upon oxidative stress [27] and radiation insult [26]. Studies have shown that phosphorylation of EGFR at its activation site Y1068 is a predictive maker to select TKIs responsive patient in NSCLC [32]. The same activation site is also reported to regulate internalization of receptor [75]. Erlotinib resistant cells have shown reduced activity of EGFR in multiple cell lines including NSCLC cells [18]. Interestingly, in our data H358-S and smoker derived NSCLC cells had decreased phosphorylation of EGFR at Y1068 and increased phosphorylation at Y1197 which is previously reported as site for internalization of receptor. Supporting our cell line observations, analysis of the signaling pathway activation in a large cohort of NSCLC tumors from TCGA database predicted that FAK signaling and EGFR internalization pathway were significantly elevated in tumors derived from the smoking patients, further supporting the role of cigarette smoke induced dysregulation of FAK signaling in lung tumorigenesis and resistance to EGFR targeted therapies.

Subsequently, our data shows that FAK inhibition using PF-562271 reinstates EGFR phosphorylation at Y1068 with subsequent reduction in phosphorylation at Y1197 and renders the cells sensitive to erlotinib. SRC is known to phosphorylate FAK at Y576/577 residue and, reports have suggested the use of dasatinib in combination therapy with TKIs, in NSCLC especially in smokers [8]. Here we report that, SRC inhibition using dasatinib was unable to restore the dependency of the cells to EGFR signaling. Our study reveals that FAK inhibition led to significant reduction in the expression of FRK in H358-S and NSCLC cells, whereas FRK expression remains unchanged upon dasatinib treatment suggesting that FRK might work downstream of FAK. Our study suggests that activation of FAK independent of SRC is sufficient to confer erlotinib resistance in NSCLC specifically in smokers.

Taken together, our data shows that cigarette smoke-induced activation of FAK signaling results in altered EGFR activity, leading to erlotinib resistance in NSCLC. In summary, our findings suggest that combination therapy targeting FAK and EGFR could prove to be an effective approach to treat NSCLC, in smoker population where only anti-EGFR therapy has shown limited success.

## MATERIALS AND METHODS

### Cell culture and SILAC labelling

Human lung cancer cell lines H358, H1299 and H1650 were obtained from American Type Culture Collection (ATCC, Manassas, VA). H358 cells were cultured in DMEM supplemented with 10% fetal bovine serum and 1% penicillin/streptomycin mixture. H1299 and H1650 were maintained in RPMI supplemented with 10% fetal bovine serum (Clontech, Mountain View, CA) and 1% penicillin/streptomycin mixture. All cells were grown in a humidified incubator maintained at 37°C and 5% CO2. H358 cells were adapted and chronically treated with cigarette smoke condensate (CSC) as described in our previous study [15]. Briefly, the cells were exposed to CSC (0.1%) for 12 months in a smoke dedicated incubator. Untreated or control cells were cultured in regular incubator. Untreated and smoke treated/exposed cells were designated as H358-P and H358-S, respectively. H358-P cells were then adapted to 13C_6_ -lysine and 13C_6_ - arginine enriched SILAC media while H358-S cells were maintained in normal media.

### Trypsin digestion and Sep-Pak cleaning

H358-P and H358-S were washed with ice cold 1X phosphate buffer saline after 12 hour of serum starvation. The cells were harvested and lysed in urea lysis buffer (20 mM HEPES pH 8.0, 9 M urea, 1 mM sodium orthovanadate, 2.5 mM sodium pyrophosphate, 1 mM phosphoglycerophosphate). Protein estimation was carried out using BCA (Pierce, Waltham, MA). Equal amount of protein from both conditions was pooled and subjected to reduction and alkylation using dithiothreitol (5mM) and iodoacetamide (10mM), respectively. Further, the lysate was diluted with HEPES buffer (20 mM) to reduce the concentration of urea below 2M and kept for overnight digestion at 37°C using trypsin (Worthington Biochemical Corp, Lakewood, NJ). Sep-Pak C18 column (Waters, Cat#WAT051910) was used to clean the peptide digest, which was further lyophilized and stored at −80°C.

### Immunoaffinity enrichment of tyrosine phosphopeptides

Enrichment of tyrosine-containing peptides was done according to protocol described earlier [13, 76]. Briefly, lyophilized peptides were resuspended in 1.4 ml of IAP buffer (50 mM MOPS pH 7.2, 50 mM NaCl, 10 mM sodium phosphate) and 1M Tris Base was used adjusted pH at 7.2. P-Tyr-1000 beads (Cell Signaling Technology, Danvers, MA) were washed with IAP buffer at 4°C. Peptides were incubated for 20 minutes with P-Tyr-1000. After washing beads with ice cold IAP buffer (thrice) and ice cold water (twice), peptides were eluted using trifluoroacetic acid (TFA) (0.15%). Eluted peptides were concentrated by vacuum centrifugation and desalted using C18 StageTips prior to mass spectrometry analysis.

### LC-MS/MS and data analysis

The enriched phosphotyrosine-containing peptides were analyzed on LTQ-Orbitrap Velos mass spectrometer (Thermo Electron, Bremen, Germany) interfaced with Easy-nLC II nanoflow liquid chromatography system (Thermo Scientific, Odense, Southern Denmark). The peptide digest was reconstituted in 0.1% formic acid and loaded on trap column (75 µm × 2 cm) packed with Magic C_18_ AQ (Michrom Bioresources, Inc., Auburn, CA, USA) (5 µm particle size, pore size 100Å). Peptides were separated on an analytical column (75 µm × 15 cm) with a linear gradient of ACN from 5 to 60% maintained at a flow rate of 350 nl/min in a 120 minutes run. MS and MS/MS analysis in Orbitrap mass analyzer was carried out at mass resolution of 60,000 and 15,000 at m/z of 400 (scan range: 350-1700 m/z). HCD fragmentations of 15 most abundant ions were subjected to MS/MS analysis (isolation width of 1.90 m/z and normalized collision energy of 39%).

Mass spectrometry data was searched against a Human RefSeq database (RefSeq 59) which was appended with frequently observed contaminants using MASCOT (v 2.2) and SEQUEST search algorithms through the Proteome Discoverer platform (v1.4, Thermo Scientific, Bremen, Germany). For both algorithms, the search parameter included - maximum of 2 missed cleavage, fixed modification of carbamidomethylation at cysteine and dynamic modification included oxidation of methionine, phosphorylation at serine, threonine and tyrosine (+79.966 Da) and SILAC labels 13C6-Lysine; 13C6-Arginine (+6.02013 Da). The precursor mass error tolerance and fragment ion mass error tolerance was set to 20 ppm and 0.1 Da, respectively. In addition, decoy database search was carried out to calculate false discovery rate (FDR). FDR of 1% was considered for peptide identification. The PhosphoRS node (Version 3.0) in the Proteome Discoverer was used to calculate the phosphorylation probability at Y residue [77] and peptides greater than 75% site localization probability was considered for further analysis.

### LC-MS/MS data availability

The raw data has been submitted to ProteomeXchange Consortium (http://proteomecentral.proteomexchange.org) via the PRIDE public data repository [78] and can be accessed using the data identifier - PXD006705.

### Western blotting

The H358-P and H358-S cells were lysed using modified RIPA lysis buffer (Merck Millipore, Billerica, MA,) containing protease inhibitors (Roche, Indianapolis, IN,) and phosphatase inhibitors (Thermo Scientific, Bremen, Germany). Western blot analysis was performed as described previously using 30 μg protein lysates [79, 80]. Briefly, lysate were resolved and transferred on nitrocellulose membranes, which was further hybridized with primary antibodies and developed using Luminol reagent (Santa Cruz Biotechnology, Dallas, TX,). Anti-EGFR, phospho-EGFR (Y1068 and Y1197), anti-FAK, phospho-FAK (Y576/577), anti-SRC, and phospho-SRC (Y416) antibodies were obtained from Cell Signaling Technology (Cell Signaling Technology, Beverly, MA). Anti-FRK antibody was obtained from Santa Cruz Biotechnology. Beta-actin antibody was obtained from Sigma (St. Louis, MO). The FAK inhibitor PF-562271 was purchased from Santa Cruz Biotechnology (Dallas, TX). Erlotinib and Dasatinib were purchased from Selleckchem (Houston, TX). DMSO was used as vehicle in all experiments.

### siRNA transfection

The H358-S, H1299 and H1650 cells were transfected with ON-TARGETplus SMARTpool control siRNA and FRK siRNA (Dharmacon, Lafayette, CO) using RNAiMAX (Invitrogen, Grand Island, NY) following manufacturer's instructions. Transfection was carried out according to previously described protocol [79]. The efficiency of transfection was determined by western blot analysis. For MTT assays, drug treatment was done 24 hours post-siRNA transfection. For western blot analysis, cells were harvested 48 hours post-transfection. Cells were subjected to invasion assays 24 hours post-transfection and stained after 48 hours.

### Invasion assays

The invasion assays were performed similar to previously described protocol using a transwell system (BD Biosciences, San Jose, CA) with Matrigel-coated filters [80, 81]. Each experiment was performed in triplicate and the experiments were repeated three times.

### Cell viability assays

Cells were seeded in 96 well plates at density of 5000 cells/well and incubated overnight. To assess the effect of erlotinib or PF-562271 on cell viability, the cells were treated at different concentration of drug in media supplemented with 5% FBS and incubated for 72 hours. Cell viability was determined using MTT reagent and absorbance at 570 nm and 650 nm was taken using Multiscan plate reader. For combination treatment of erlotinib and PF-562271 or erlotinib and dasatinib, the cells were treated with PF-562271 or dasatinib for 24 hoursfollowed by erlotinib treatment. The MTT reading was taken post 72 hours of erlotinib treatment. All treatments was done in triplicates and each experiment was repeated thrice. The mean value of cell viability from replicates was taken to express the percentage of viability relative to DMSO as control treatment.

### Phosphotyrosine based pathway analysis

The pathway analysis of the differentially expressed proteins was carried out using QIAGEN's Ingenuity® Pathway Analysis (IPA®, QIAGEN Redwood City, www.qiagen.com/ingenuity) and STITCH tool (version 5.0) [82]. We had considered top 5 enriched pathways for both the tools.

### Expression data processing

RNA-Seq data was retrieved from TCGA database. Data preprocessing and normalization steps were performed in R version 3.1.0 using DEseq package from Bioconductor. To adjust for the possible batch and processing effect we have employed XPN algorithm (R package, CONOR), as previously described [83]. The resulting matrix contained mRNA expression information for over 20K genes across all analyzed samples. Normalized gene expression data were loaded into iPANDA [16, 17]. The software enables calculation of the Pathway Activation Score (PAS) for each of the pathways analyzed, a value which serves as a quantitative measure of differential pathway activation. A collection of intracellular signaling pathways strongly implicated with various solid malignancies was obtained from the NCI Pathway Interaction Database, and used for the computational algorithm as described previously [16, 17]. Calculated PAS values for pathways associated with FAK signaling are shown.

### *In vivo* studies

H358-P (2×10^6^) and H358-S cells (2×10^6^) were injected subcutaneously in flanks of 6-week-old NOD-SCID male mice. Two mice for each cell line were used for study. Mice were housed in pathogen free conditions and experiments were conducted according to the institutional ethical guidelines at the experimental animal facility of Indian Institute of Sciences, Bangalore, India. The measurement of tumor size was done using caliper every 3 and tumor volume was calculated using following formula - (π/6(d1×d2) 3/2) [84].

### Statistical analysis

All statistical analysis was performed using Graphpad Prism software (version 6.0) (2-tailed t-test) and data with p<0.05 were considered statistically significant.

## SUPPLEMENTARY MATERIALS FIGURES AND TABLES





## Abbreviations

BCA: Bicinchoninic acid assay
CSC: Cigarette smoke condensate
EGFR: Epidermal growth factor receptor
FAK: Focal adhesion kinase
IAP: Immunoaffinity purification
NSCLC: Non small cell lung carcinoma
SILAC: Stable isotope labeling by amino acids in cell culture
