# Correction: Targeting focal adhesion kinase overcomes erlotinib resistance in smoke induced lung cancer by altering phosphorylation of epidermal growth factor receptor

**DOI:** 10.18632/oncoscience.546

**Published:** 2021-09-23

**Authors:** Hitendra S. Solanki, Remya Raja, Alex Zhavoronkov, Ivan V. Ozerov, Artem V. Artemov, Jayshree Advani, Aneesha Radhakrishnan, Niraj Babu, Vinuth N. Puttamallesh, Nazia Syed, Vishalakshi Nanjappa, Tejaswini Subbannayya, Nandini A. Sahasrabuddhe, Arun H. Patil, T.S. Keshava Prasad, Daria Gaykalova, Xiaofei Chang, Rachana Sathyendran, Premendu Prakash Mathur, Annapoorni Rangarajan, David Sidransky, Akhilesh Pandey, Evgeny Izumchenko, Harsha Gowda, Aditi Chatterjee

**Affiliations:** ^1^Institute of Bioinformatics, International Tech Park, Bangalore 560066, India; ^2^School of Biotechnology, Kalinga Institute of Industrial Technology, Bhubaneswar, Odisha 751024, India; ^3^Insilico Medicine, Inc., Emerging Technology Centers, Johns Hopkins University at Eastern, Baltimore, MD 21218, USA; ^4^Manipal Academy of Higher Education, Manipal, Karnataka 576104, India; ^5^School of Biotechnology, Amrita University, Kollam 690525, India; ^6^Center for Systems Biology and Molecular Medicine, Yenepoya (Deemed to be University), Mangalore 575018, India; ^7^NIMHANS-IOB Proteomics and Bioinformatics Laboratory, Neurobiology Research Centre, National Institute of Mental Health and Neurosciences, Bangalore 560029, India; ^8^Department of Otolaryngology-Head and Neck Surgery, Johns Hopkins University School of Medicine, Baltimore, MD 21231, USA; ^9^Department of Molecular Reproduction, Development and Genetics, Indian Institute of Science, Bangalore, 560012, India; ^10^McKusick-Nathans Institute of Genetic Medicine, Johns Hopkins University School of Medicine, Baltimore, MD 21205, USA; ^11^Department of Biological Chemistry, Johns Hopkins University School of Medicine, Baltimore, MD 21205, USA; ^12^Department of Oncology, Johns Hopkins University School of Medicine, Baltimore, MD 21205, USA; ^13^Department of Pathology, Johns Hopkins University School of Medicine, Baltimore, MD 21205, USA; ^*^These authors contributed equally to the manuscript

Original article: Oncoscience. 2018; 5:21–38.  . https://doi.org/10.18632/oncoscience.395

**This article has been corrected:** Due to errors during figure assembly, the image for panel 3, column 1 in Figure 4B is an accidental duplicate of the image in panel 3, column 1 of Figure 4A. The corrected Figure 4 is shown below. The authors declare that these corrections do not change the results or conclusions of this paper.

**Original article**: Oncoscience. 2018 Feb 23;5(1-2):21–38

**PMCID**: PMC5854290 **PMID**: 29556515

**DOI**: 10.18632/oncoscience.395

**Figure 4:**
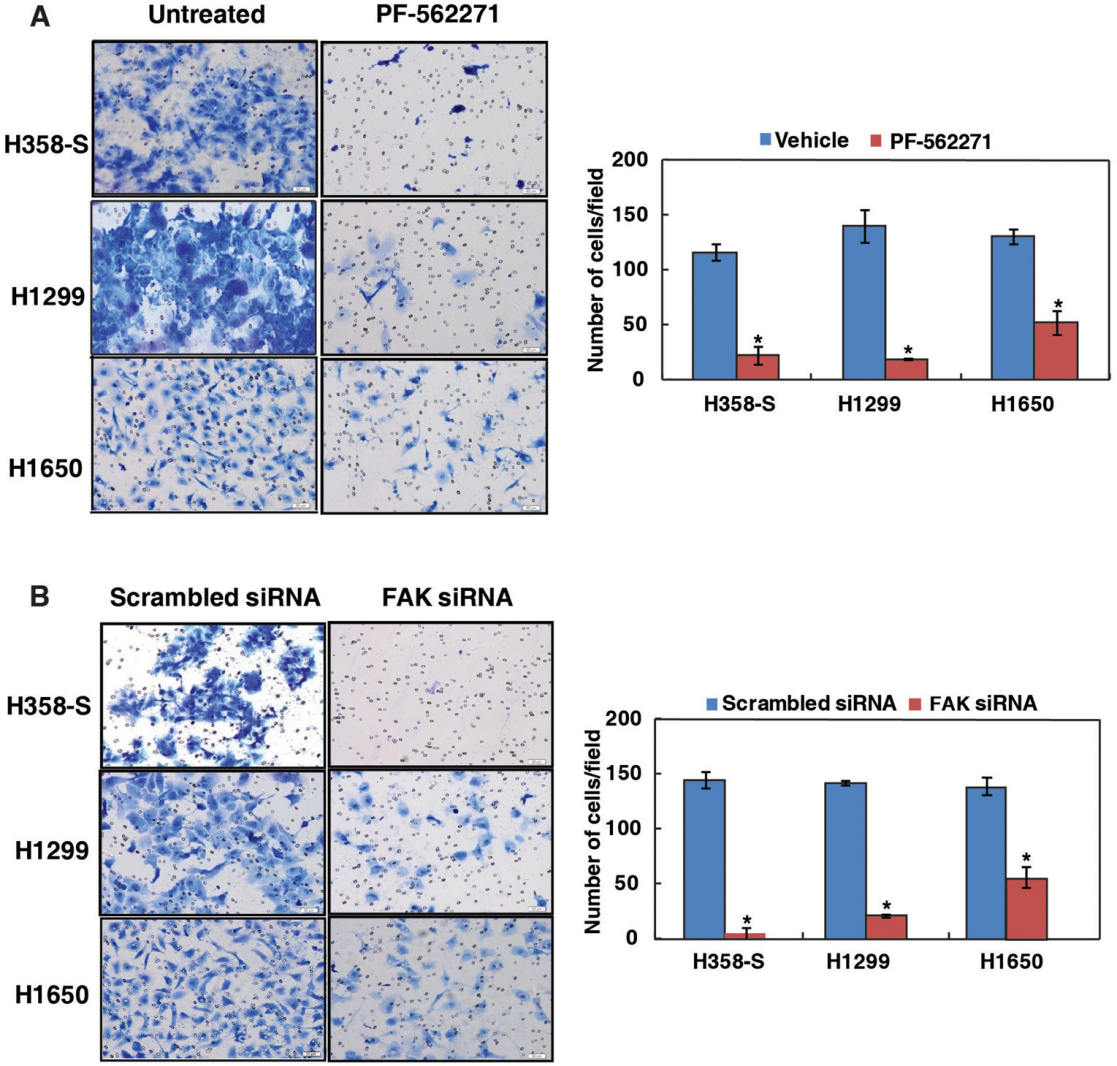
Inhibition of FAK decreases the invasive property of lung cancer cells. Invasion assays were carried out in a transwell system using Matrigel-coated filters and the number of cells that migrated to the lower chamber was counted. Cells that migrated are visualized following methylene blue staining in H358-S and NSCLC cell lines, H1299 and H1650 as indicated. (**A**) Cells were treated with either DMSO (vehicle control) or PF-562271 and invaded cells were photographed. Representative images were photographed at a magnification 10x. Invaded cells were counted and relative changes in invasive ability of H358-S and NSCLC cells upon inhibition with PF-562271 was calculated and represented graphically (^*^p < 0.05). (**B**) Cells were transfected with either scrambled siRNA or FAK siRNA and invaded cells were photographed. Invaded cells were counted and relative changes in invasive ability of H358-S and NSCLC cells upon FAK silencing was calculated and represented graphically (^*^p < 0.05). Representative images were photographed at a magnification 10x.

