# Supplementary material for: Targeting focal adhesion kinase overcomes erlotinib resistance in smoke induced lung cancer by altering phosphorylation of epidermal growth factor receptor
Source: Oncoscience. 2018 Feb 23;5(1-2):21–38. doi: 10.18632/oncoscience.395 (PMC5854290; doi:10.18632/oncoscience.395)
Supplement: Supplementary file 1 [file oncoscience-05-0021-s001.pdf]

SUPPLEMENTARY MATERIALS

Supplementary Figure 1

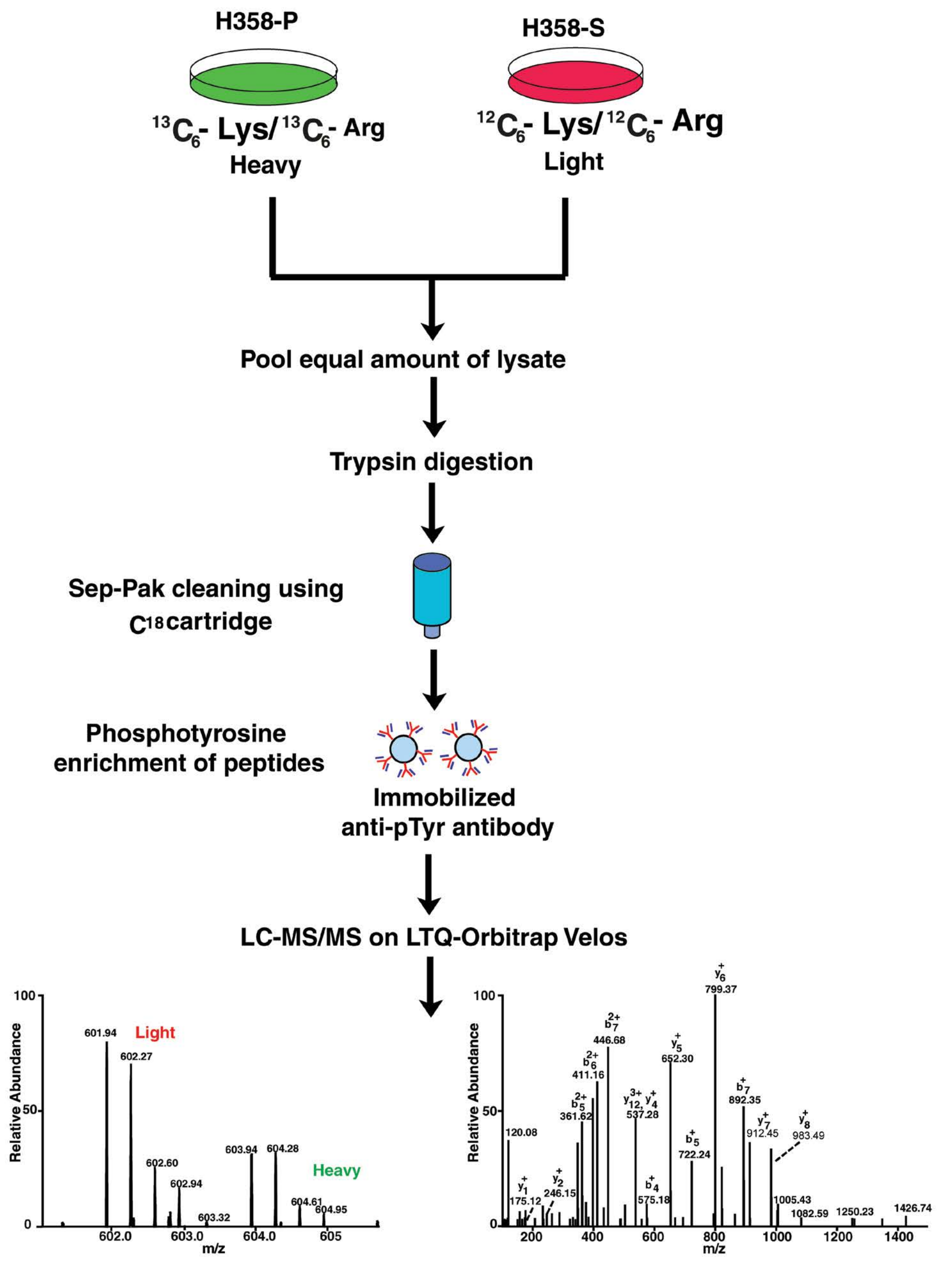

Supplementary Figure 1. Schematic workflow followed to identify proteome differentially phosphorylated at tyrosine residue upon chronic exposure to cigarette smoke. H358-P and H358-S cells were cultured in heavy media enriched with 13C6-Lys/13C6-Arg and light media (12C6-Lys/12C6-Arg), respectively. Equal amount of lysate from both populations were pooled and subjected to in-solution trypsin digestion, followed by Sep-Pak cleaning. The enrichment of tyrosine containing phosphopeptide was carried out using pY1000 antibody followed by mass spectrometry-based proteomic analysis to identify differentially phosphorylated proteins.

Supplementary Figure 2

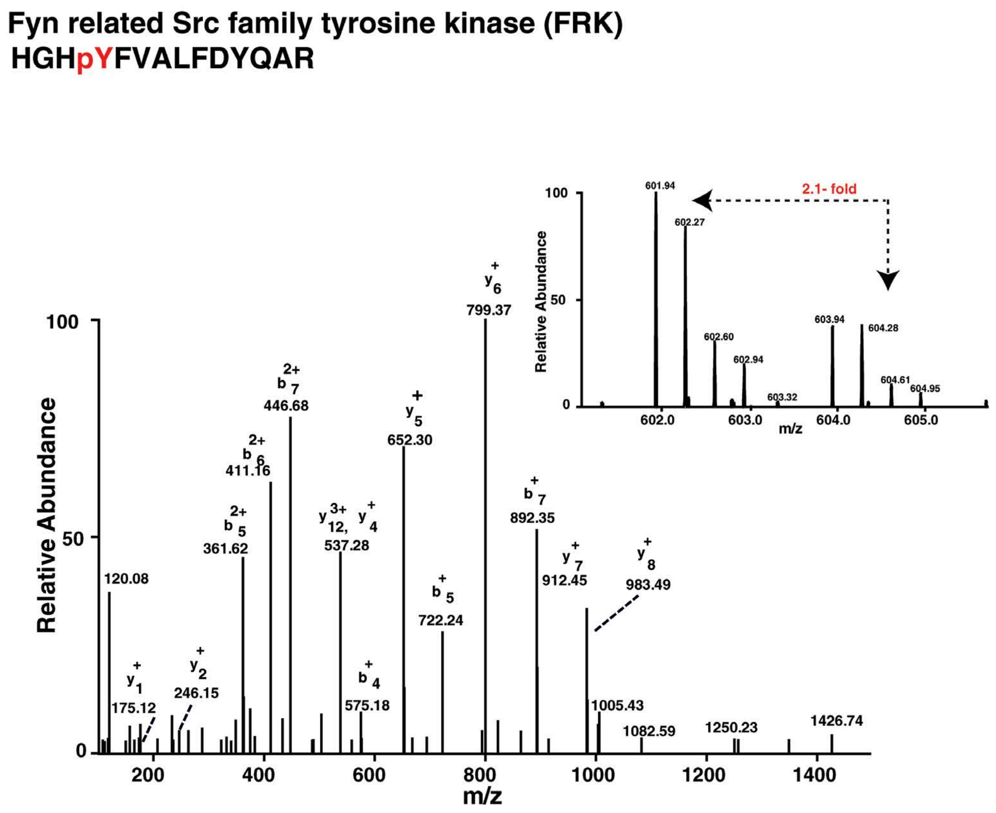

Supplementary Figure 2: Representative MS/MS spectra of hyperphosphorylated peptide of FRK in H358-S compared to H358-P cells

Supplementary Figure 3

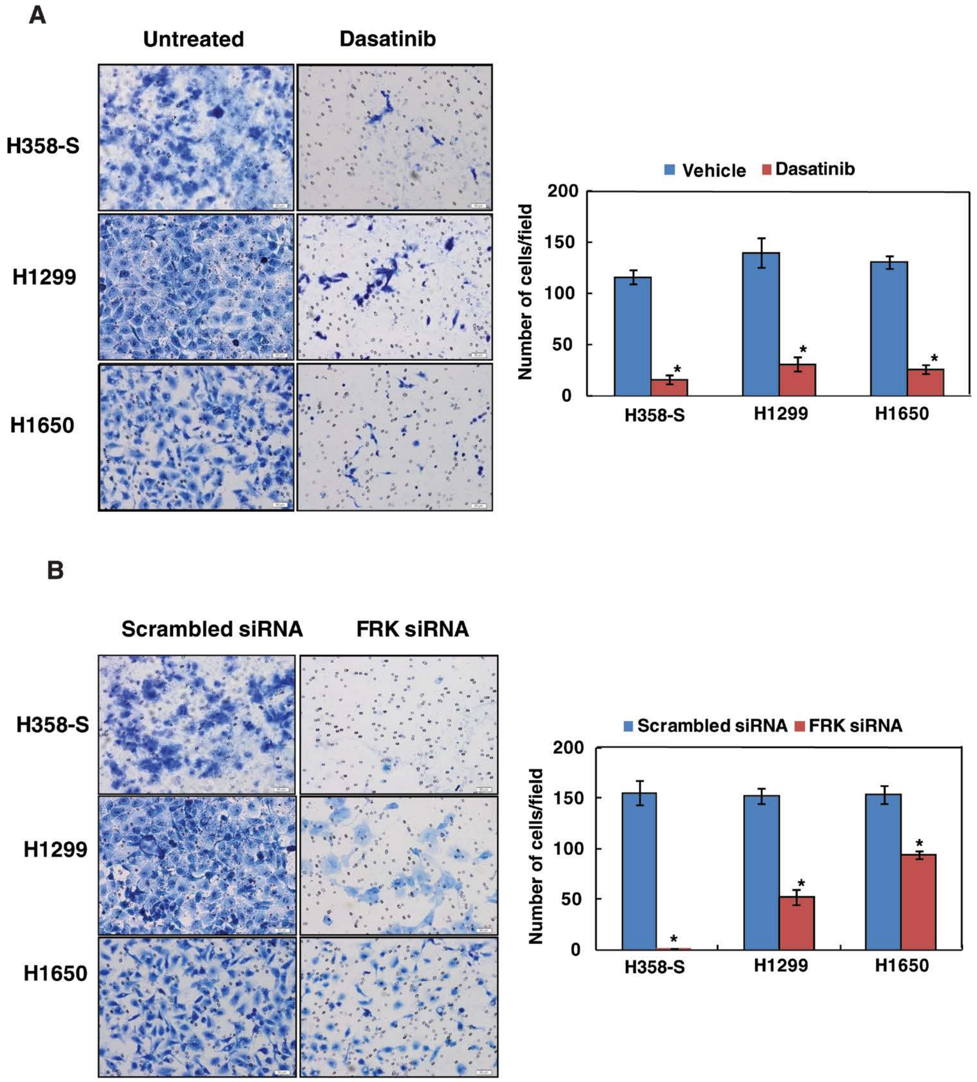

Supplementary Figure 3: Inhibition of SRC and silencing of FRK decreases the invasive property of lung cancer cells (A) Cells were treated with either DMSO (vehicle control) or Dasatinib and invaded cells were photographed. Graphical representation of invasive ability of H358-S and NSCLC cells upon inhibition with dasatinib (* $p<0.05$). Representative images were photographed at a magnification (10x). (B) Invasion assay was performed after tranfection with scrambled or FRK siRNA and invaded cells were photographed. Graphical representation of invasive ability of H358-S and NSCLC cells upon inhibition with FRK siRNA (* $p<0.05$). Representative images were photographed at a magnification (10x).

Supplementary Table 1: Complete list of identified phosphopeptides with fold change using Sequest and Mascot search algorithms.(See additional supplementary material file)

Supplementary Table 2: The pathway analysis of the differentially expressed proteins was carried out using STITCH and Ingenuity Pathway Analysis (IPA) tool. Top 5 pathways enriched by (A) STITCH and (B) IPA are shown.

Supplementary Table 2A

# KEGG pathway ID	Top Pathways	Associated Molecules
4510	Focal adhesion	ARHGAP35,ARHGAP5,BCAR1,CAV1,ERBB2,JUN,PTK2,PXN,SHC1,VCL
4520	Adherens junction	BAIAP2,CTNNA1,ERBB2,INSR,MLLT4,PARD3,PVRL4,VCL,WASL
4670	Leukocyte transendothelial migration	ARHGAP35,ARHGAP5,BCAR1,CTNNA1,MAPK12,MLLT4,PTK2,PTK2B,PXN,VCL
4062	Chemokine signaling pathway	BCAR1,PARD3,PRKCD,PTK2,PTK2B,PXN,SHC1,STAT1,WASL
4810	Regulation of actin cytoskeleton	ARHGAP35,BAIAP2,BCAR1,PTK2,PXN,VCL,WASL

Supplementary Table 2 B

Top Networks	Associated Molecules
Leukocyte Extravasation Signaling	PXN,MAPK1,PTK2B,PIK3R1,WASL,CTNNA1,MAPK9,PLCG1,AFDN,MAPK13,MAPK12,BCAR1,PTK2,ARHGAP5,MAPK14,PTPN11,GAB1,PRKCD,ARHGAP35,IRS2,PIK3R2,VCL,CTTN
Integrin Signaling	PXN,MAPK1,PIK3R1,WASL,PLCG1,TLN1,NCK1,BCAR3,BCAR1,ARHGAP5,PTK2,SHC1,PTPN11,GAB1,MAPK3,CAV1,IRS2,ITGB4,PIK3R2,VCL,CTTN,NEDD9
Paxillin Signaling	PXN,MAPK1,PTK2B,PIK3R1,MAPK9,TLN1,NCK1,MAPK13,MAPK12,BCAR1,PTK2,MAPK14,GAB1,PTPN11,IRS2,ITGB4,VCL,PIK3R2
Renin-Angiotensin Signaling	MAPK1,PTK2B,PIK3R1,MAPK9,PLCG1,MAPK13,MAPK12,PTK2,SHC1,MAPK14,GAB1,PTPN11,MAPK3,PRKCD,IRS2,PIK3R2,STAT1
PAK Signaling	PXN,PTK2B,MAPK1,PIK3R1,WASL,MAPK9,NCK1,MAPK12,PTK2,SHC1,GAB1,PTPN11,MAPK3,EPHB3,IRS2,PIK3R2
