# Supplementary material for: Targeting focal adhesion kinase overcomes erlotinib resistance in smoke induced lung cancer by altering phosphorylation of epidermal growth factor receptor
Source: Oncoscience. 2018 Feb 23;5(1-2):21–38. doi: 10.18632/oncoscience.395 (PMC5854290; doi:10.18632/oncoscience.395)
Supplement: Supplementary file 2 [file oncoscience-05-0021-s002.pdf]

www.impactjournals.com/oncoscience Oncoscience, Vol. 5(1-2), January 2018

SUPPLEMENTARY MATERIALS

Supplementary Table 1:Complete list of identified phosphopeptides with fold change using Sequest and Mascot search algorithms. See pages below.

Solanki *et al*., 2017. Targeting Focal Adhesion Kinase overcomes erlotinib resistance in smoke induced lung cancer by altering phosphorylation of Epidermal Growth Factor R[illegible]

Supplementary Table 1: Complete list of identified phosphopeptides with fold change using Sequest and Mascot search algorithms.

Gene Symbol	Gene Id	Description	Protein Group Accession	Sequence	Modifications	PhosphoRS Site Probabilities	Phospho Site (Protein)	Phospho Site (Peptide)	# PSMs	# Protein Groups	Phosphowindow	Xcorr	IonScore	MH+ [Da]	# Missed Cleavages	pY Light/Heavy
LSR	51599	lipolysis-stimulated lipoprotein receptor isoform 5	386781463	SSSAGGQGSyVPLLR	Y10(Phospho); R15(Label:13C(6))	S(1): 0.0; S(2): 0.0; S(3): 0.0; S(9): 2.5; Y(10): 97.5	Y264	Y10;	7	1	SAGGQGSyVPLLRDT	3.73	84	1564.74651	0	0.25
GAB1	2549	GRB2-associated-binding protein 1 isoform b	46370073	SSGSGSSVADERVDyVVVDQQK	Y15(Phospho)	S(1): 0.0; S(2): 0.0; S(4): 0.0; S(6): 0.0; S(7): 0.0; Y(15): 100.0	Y659	Y15;	15	1	VADERVDyVVVDQQK	5.55	42	2392.06437	1	2.29

FLNB	2317	filamin-B isoform 4	256222415	SSTETCySAIPK	C6(Carbamidomethyl); Y7(Phospho)	S(1): 0.0; S(2): 0.0; T(3): 0.1; T(5): 0.1; Y(7): 99.7; S(8): 0.1	Y2478	Y7;	4	1	RSSTETCySAIPKAS	1.74	26	1423.58208	0	1.72
HNRNPA1	3178	heterogeneous nuclear ribonucleoprotein A1 isoform a	4504445	SSGPYGGGGQyFAKPR	Y11(Phospho)	S(1): 0.0; S(2): 0.0; Y(5): 0.0; Y(11): 100.0	Y295	Y11;	4	1	PYGGGGQyFAKPRNQ	4.11	94	1708.74761	0	1.09
ARHGAP35	2909	rho GTPase-activating protein 35	150417981	SVSSSPWLPQDGFDPSDyAEPMDAVVKPR	Y18(Phospho)	S(1): 0.0; S(3): 0.0; S(4): 0.3; S(5): 0.0; S(16): 5.1; Y(18): 94.6	Y1087	Y18;	12	1	DGFDPSDyAEPMDAV	3.65		3257.45285	0	0.25
PRKCD	5580	protein kinase C delta type	31377782	SDSASSEPVGIyQGFEKK	Y12(Phospho)	S(1): 0.0; S(3): 0.0; S(5): 0.0; S(6): 0.0; Y(12): 100.0	Y313	Y12;	22	1	SSEPVGIyQGFEKKT	3.75	46	2008.89055	1	4.44

PRKCD	5580	protein kinase C delta type	31377782	SDSASSEPVGIyQGFEKK	Y12(Phospho)	S(1): 0.0; S(3): 0.0; S(5): 0.0; S(6): 0.0; Y(12): 100.0	Y313	Y12;	22	1	SSEPVGIyQGFEKKT	3.75		2008.88962	1	4.29
PRKCD	5580	protein kinase C delta type	31377782	SDSASSEPVGIyQGFEK	Y12(Phospho)	S(1): 0.0; S(3): 0.0; S(5): 0.0; S(6): 0.0; Y(12): 100.0	Y313	Y12;	12	1	SSEPVGIyQGFEKKT	4.07	53	1880.79521	0	3.97
MPZL1	9019	myelin protein zero-like protein 1 isoform a precursor	4506357	SESVVyADIR	Y6(Phospho)	S(1): 0.0; S(3): 0.0; Y(6): 100.0	Y263	Y6;	19	1	NKSESVVyADIRKN_	2.53	57	1218.5396	0	5.51
UMPS	7372	uridine 5'-monophosphate synthase	4507835	SGLSSPIyIDLR	Y8(Phospho); R12(Label:13C(6))	S(1): 0.0; S(4): 0.0; S(5): 0.0; Y(8): 100.0	Y37	Y8;	3	1	SGLSSPIyIDLRGIV	1.67	63	1406.70452	0	0.89

VCL	7414	vinculin isoform meta-VCL	7669550	SFLDSGyR	Y7(Phospho)	S(1): 0.0; S(5): 0.1; Y(7): 99.9	Y822	Y7;	8	1	KSFLDSGyRILGAVA	1.62	39	1024.41301	0	0.37
C11orf52	91894	uncharacterized protein C11orf52	131889517	SPGLMSEDSNLHyADIQVCSRPHAR	Y13(Phospho); C19(Carbamidomethyl)	S(1): 0.0; S(6): 8.4; S(9): 1.0; Y(13): 82.3; S(20): 8.4	Y78	Y13;	2	1	SEDSNLHyADIQVCS	3.08		2920.29262	0	2.12
SHB	6461	SH2 domain-containing adapter protein B	106879210	SKAGKGESAGyMEPYEAQR	K2(Label:13C(6)); K5(Label:13C(6)); Y11(Phospho); R19(Label:13C(6))	S(1): 0.0; S(8): 0.0; Y(11): 100.0; Y(15): 0.0	Y268	Y11;	11	1	GKGESAGyMEPYEAQ	4.34	79	2156.98125	2	0.29
SHB	6461	SH2 domain-containing adapter protein B	106879210	SKAGKGESAGyMEPYEAQR	K2(Label:13C(6)); K5(Label:13C(6)); S8(Phospho); R19(La	S(1): 0.0; S(8): 1.7; Y(11): 98.3; Y(15): 0.0	Y268	Y11;	11	1	GKGESAGyMEPYEAQ	4.34	46	2156.9802	2	0.27

					bel:13C (6))											
ITGB 1	3688	integri n beta-1 isofor m 1A precur sor	19743823	SAVTT VVNP KyEG K	K10(La bel:13C (6)); Y11(Ph ospho); K14(La bel:13C (6))	S(1): 0.0; T(4): 0.0; T(5): 0.0; Y(11): 100.0	Y795	Y11;	3	1	TTVV NPKyE GK_	3.06	40	1584. 8051	1	0.83
TGM 2	7052	protei n- gluta mine gamm a- gluta myltra nsfera se 2 isofor m a	39777597	SEGtY CCGP VPVR	Y5(Pho spho); C6(Car bamido methyl) ; C7(Car bamido methyl)	S(1): 0.0; T(4): 98.3; Y(5): 1.7	T368	T4;	5	1	PQEKS EGtYC CGPVP	3.26	80	1561. 61577	0	12.70

ANXA2	302	annexin A2 isoform 2	209862831	SYSPyDMLESIR	Y5(Phospho)	S(1): 0.0; Y(2): 0.0; S(3): 0.0; Y(5): 100.0; S(10): 0.0	Y238	Y5;	4	1	RYKSYSPyDMLESIR	2.83	66	1540.63786	0	0.64
ANXA2	302	annexin A2 isoform 2	209862831	SYSPyDMLESIRK	Y5(Phospho)	S(1): 0.0; Y(2): 0.0; S(3): 0.0; Y(5): 100.0; S(10): 0.0	Y238	Y5;	4	1	RYKSYSPyDMLESIR	2.96	43	1668.73638	1	0.63
ANXA2	302	annexin A2 isoform 2	209862831	SYSPyDMLESIRK	Y5(Phospho); R12(Label:13C(6)); K13(Label:13C(6))	S(1): 0.0; Y(2): 0.0; S(3): 0.0; Y(5): 100.0; S(10): 0.0	Y238	Y5;	4	1	RYKSYSPyDMLESIR	2.96	43	1680.77524	1	0.61
IRS2	8660	insulin receptor substrate 2	38683860	SYKAPYTCGGDSDQyVLMSSPVGR	C8(Carbamidomethyl); Y15(Phospho)	S(1): 0.0; Y(2): 0.0; Y(6): 0.0; T(7): 0.0; S(12): 0.0; Y(15): 100.0; S(19): 0.0; S(20): 0.0	Y823	Y15;	10	1	CGGDSDQyVLMSSPV	3.60	48	2718.15946	1	9.92

ANXA2	302	annexin A2 isoform 2	209862831	SLYyYIQQDTK	Y4(Phospho); K11(Label:13C(6))	S(1): 0.0; Y(3): 0.0; Y(4): 99.9; Y(5): 0.0; T(10): 0.0	Y317	Y4;	4	1	KYGKSLYyYIQQDTK	2.61	56	1507.67986	0	0.43
SLC38A2	54407	sodium-coupled neutral amino acid transporter 2	21361602	SHyADVDPENQNFLLESNLGKK	Y3(Phospho)	S(1): 0.0; Y(3): 100.0; S(17): 0.0	Y41	Y3;	4	1	QAALKSHyADVDPEN	6.35	67	2598.18821	1	2.17
ANXA2	302	annexin A2 isoform 2	209862831	SLyYYIQQDTK	Y3(Phospho); K11(Label:13C(6))	S(1): 0.0; Y(3): 100.0; Y(4): 0.0; Y(5): 0.0; T(10): 0.0	Y316	Y3;	4	1	RKYGKSLyYYIQQDT	2.61	67	1507.68181	0	0.36
PIK3R2	5296	phosphatidylinositol 3-kinase regulatory subunit beta	4826908	SREYDQLyEEYTR	Y8(Phospho)	S(1): 0.0; Y(4): 0.0; Y(8): 100.0; Y(11): 0.0; T(12): 0.0	Y464	Y8;	15	1	SREYDQLyEEYTRTS	4.23	56	1831.75334	1	3.02

PIK3R1	5295	phosphatidylinositol 3-kinase regulatory subunit alpha isoform 1	32455248	SREYDRLyEEYTR	Y8(Phospho)	S(1): 0.0; Y(4): 0.0; Y(8): 100.0; Y(11): 0.0; T(12): 0.0	Y467	Y8;	5	1	SREYDRLyEEYTRTS	2.70		1859.79526	2	2.71
PIK3R1	5295	phosphatidylinositol 3-kinase regulatory subunit alpha isoform 1	32455248	SREYDRLyEEYTR	Y8(Phospho)	S(1): 0.0; Y(4): 0.0; Y(8): 100.0; Y(11): 0.0; T(12): 0.0	Y467	Y8;	5	1	SREYDRLyEEYTRTS	2.70		1859.79424	2	2.66
CTNND1	1500	catenin delta-1 isoform 2A	332688222	SMGYDDLDyGMMSDYGTAR	Y9(Phospho)	S(1): 0.0; Y(4): 0.0; Y(9): 100.0; S(13): 0.0; Y(15): 0.0; T(17): 0.0	Y242	Y9;	15	1	MGYDDLDyGMMSDYG	4.30	96	2227.80278	0	1.44

ICK	22858	serine/threonine-protein kinase ICK	7662388	SKPPYTDyVSTR	Y8(Phospho)	S(1): 0.0; Y(5): 1.8; T(6): 1.8; Y(8): 94.7; S(10): 1.8; T(11): 0.0	Y159	Y8;	7	1	SKPPYTDyVSTRWYR	2.21	45	1493.66655	0	2.27
PLEKHA6	22874	pleckstrin homology domain-containing family A member 6	37595548	SEDIyADPAAYVMR	Y5(Phospho); R14(Label:13C(6))	S(1): 0.0; Y(5): 100.0; Y(11): 0.0	Y492	Y5;	5	1	PPRSEDIyADPAAYV	2.75	60	1686.7188	0	0.30
PTK2	5747	focal adhesion kinase 1 isoform a	24476013	SNDKVyENVTGLVK	Y6(Phospho)	S(1): 0.0; Y(6): 100.0; T(10): 0.0	Y925	Y6;	5	1	DRSNDKVyENVTGLV	2.75	54	1645.78105	1	1.21
NEDD9	4739	enhancer of filamentation 1 isoform 3	215422375	SWMDDyDyVHLQGK	Y6(Phospho); Y8(Phospho)	S(1): 0.0; Y(6): 100.0; Y(8): 100.0	Y629; Y631	Y6; Y8;	3	1	ERSWMDDyDYVHLQG;SWMDDYDyVHLQ	2.14	46	1916.69695	0	14.63

											GKE					
CTNND1	1500	catenin delta-1 isoform 2A	332688222	SLDNNySTPNER	Y6(Phospho); R12(Label:13C(6))	S(1): 0.0; Y(6): 98.5; S(7): 1.5; T(8): 0.0	Y844	Y6;	6	1	TKSLDNNySTPNERG	1.54	56	1495.61528	0	0.60
PKP4	8502	plakophilin-4 isoform b	53829376	StTNYVDFYSTK	Y5(Phospho); K12(Label:13C(6))	S(1): 0.1; T(2): 97.3; T(3): 0.1; Y(5): 2.5; Y(9): 0.0; S(10): 0.0; T(11): 0.0	T1122	T2;	5	1	TQYGLKStTNYVDFY	2.31		1511.63994	0	0.53
IRS2	8660	insulin receptor substrate 2	38683860	SSSSNLGADDGyMPMTPGAALAGSGSGSCR	Y12(Phospho); C29(Carbamidomethyl)	S(1): 0.2; S(2): 0.2; S(3): 0.2; S(4): 0.2; Y(12): 99.0; T(16): 0.0; S(24): 0.0; S(26): 0.0; S(28): 0.0	Y653	Y12;	20	1	NLGADDGyMPMTPGA	2.76	33	2941.17533	0	16.99

PVRL4	81607	poliovirus receptor-related protein 4 precursor	222136611	SySTLTTVR	Y2(Phospho); R9(Label:13C(6))	S(1): 1.7; Y(2): 98.3; S(3): 0.0; T(4): 0.0; T(6): 0.0; T(7): 0.0	Y445	Y2;	7	1	EEPEGRSySTLTTVR	2.07	58	1113.52788	0	0.27
PIK3R2	5296	phosphatidylinositol 3-kinase regulatory subunit beta	4826908	sREYDQLYEEYTR	Y4(Phospho)	S(1): 100.0; Y(4): 0.0; Y(8): 0.0; Y(11): 0.0; T(12): 0.0	S457	S1;	15	1	HQQYQDKsREYDQLY	4.23	44	1831.752	1	2.79
HNRNPA1	3178	heterogeneous nuclear ribonucleoprotein A1 isoform a	4504445	SSGPyGGGGQYFAKPR	Y5(Phospho); K14(Label:13C(6)); R16(Label:13C(6))	S(1): 2.0; S(2): 2.0; Y(5): 96.0; Y(11): 0.0	Y289	Y5;	4	1	GGRSSGPyGGGGQYF	4.11	94	1720.78752	0	1.50
KRT7	3855	keratin, type II	67782365	SAyGGPVGAGIR	Y3(Phospho)	S(1): 2.0; Y(3): 98.0	Y55	Y3;	4	1	RVAVRSAyGGPVG	3.15	71	1184.54497	0	0.87

		cytoskeletal 7									AG					
HNRNPA1	3178	heterogeneous nuclear ribonucleoprotein A1 isoform a	4504445	SSGPyGGGGQYFAKPR	Y5(Phospho)	S(1): 2.1; S(2): 2.1; Y(5): 95.8; Y(11): 0.0	Y289	Y5;	4	1	GGRSSGPyGGGGQYF	4.11	33	1708.74479	0	1.46
PRKCD	5580	protein kinase C delta type	31377782	SDsASSEPVGIyQGFEKK	S6(Phospho); Y12(Phospho)	S(1): 2.5; S(3): 97.5; S(5): 0.0; S(6): 0.0; Y(12): 100.0	S304; Y313	S3; Y12;	22	1	RASRRSDsASSEPVG;SSEPVGIyQGFEKKT	3.75		2088.85654	1	13.38
PRKCD	5580	protein kinase C delta type	31377782	SDsASSEPVGIyQGFEKK	S3(Phospho); Y12(Phospho)	S(1): 2.5; S(3): 97.5; S(5): 0.1; S(6): 0.0; Y(12): 100.0	S304; Y313	S3; Y12;	22	1	RASRRSDsASSEPVG;SSEPVGIyQGFEKKT	3.75	40	2088.85503	1	11.69

IRS2	8660	insulin receptor substrate 2	38683860	SDDyMPMSPASVSAPK	Y4(Phospho)	S(1): 2.9; Y(4): 97.1; S(8): 0.0; S(11): 0.0; S(13): 0.0	Y675	Y4;	4	1	GSCRSDDyMPMSPAS	2.61		1762.71684	0	16.22
ARHGAP35	2909	rho GTPase-activating protein 35	150417981	SVSSSPWLPQDGFDPsDYAEPMDAVVKPR	S5(Phospho); S16(Phospho)	S(1): 47.8; S(3): 47.8; S(4): 2.2; S(5): 2.2; S(16): 95.6; Y(18): 4.4	S1085	S16;	12	1	PQDGFDPsDYAEPMD	3.65		3337.41355	0	0.56
PRKCD	5580	protein kinase C delta type	31377782	SDSASSEPVGIyQGFEK	S3(Phospho); Y12(Phospho)	S(1): 50.0; S(3): 50.0; S(5): 0.0; S(6): 0.0; Y(12): 100.0	Y313	Y12;	12	1	SSEPVGIyQGFEKKT	4.07	40	1960.75591	0	8.75
PTPRE	5791	receptor-type tyrosine-protein phosphatase epsilon isoform 2	18860859	VVQDFIDIFSDyANFK	Y12(Phospho)	S(10): 0.0; Y(12): 100.0	Y638	Y12;	16	1	FIDIFSDyANFK_	3.87	57	2000.90483	0	0.31

PTPRE	5791	receptor-type tyrosine-protein phosphatase epsilon isoform 2	18860859	VVQDFIDIFSDyANFK	Y12(Phospho); K16(Label:13C(6))	S(10): 0.0; Y(12): 100.0	Y638	Y12;	16	1	FIDIFSDyANFK_	3.87	43	2006.9282	0	0.32
GSK3B	2932	glycogen synthase kinase-3 beta isoform 2	225903437	QLVRGEPNVSyICSR	R4(Label:13C(6)); Y11(Phospho); C13(Carbamidomethyl); R15(Label:13C(6))	S(10): 2.7; Y(11): 97.3; S(14): 0.0	Y216	Y11;	4	1	RGEPNVSyICSRYYR	3.26		1869.90616	1	0.51
EGFR	1956	epidermal growth factor receptor isofor	29725609	EAKPNGIFKGSTAENAEyLR	Y18(Phospho)	S(11): 0.0; T(12): 0.0; Y(18): 100.0	Y1197	Y18;	3	1	STAENAEyLRVAPQS	3.15		2275.07608	1	2.64

		m a precursor																
ARHGAP5	394	rho GTPase-activating protein 5 isoform b	71834861	VPLAHPEDMDPSDNyAEPIDTIFK	Y15(Phospho); K24(Label:13C(6))	S(12): 0.1; Y(15): 99.9; T(21): 0.0	Y1091	Y15;	4	1	DMDPSDNyAEPIDTI	3.38	31	2800.25919	0	0.09		
BCAR1	9564	breast cancer anti-estrogen resistance protein 1 isoform 7	282398123	VLPPEVADGGVVDSGVyAVPPPAER	Y17(Phospho); R25(Label:13C(6))	S(14): 0.1; Y(17): 99.9	Y408	Y17;	13	1	GVVDSGVyAVPPPAE	2.31		2576.26909	0	1.43		
KRT7	3855	keratin, type II cytoskeletal 7	67782365	LSSARPGGLGSSSLyGLGASRPR	Y15(Phospho)	S(2): 0.0; S(3): 0.0; S(11): 0.0; S(12): 0.0; S(13): 0.3; Y(15): 99.3; S(20): 0.3	Y40	Y15;	31	1	GLGSSSLyGLGASRP	3.55		2326.16738	0	0.69		

PRKCD	5580	protein kinase C delta type	31377782	RSDSASSEPVGIyQGFEKK	Y13(Phospho)	S(2): 0.0; S(4): 0.0; S(6): 0.0; S(7): 0.0; Y(13): 100.0	Y313	Y13;	18	1	SSEPVGIyQGFEKKT	5.12	59	2164.98648	2	6.23
PRKCD	5580	protein kinase C delta type	31377782	RSDSASSEPVGIyQGFEK	Y13(Phospho)	S(2): 0.0; S(4): 0.0; S(6): 0.0; S(7): 0.0; Y(13): 100.0	Y313	Y13;	14	1	SSEPVGIyQGFEKKT	5.14	79	2036.89739	1	4.79
PRKCD	5580	protein kinase C delta type	31377782	RSDSASSEPVGIyQGFEK	Y13(Phospho)	S(2): 0.0; S(4): 0.0; S(6): 0.0; S(7): 0.0; Y(13): 100.0	Y313	Y13;	14	1	SSEPVGIyQGFEKKT	5.14	43	2036.89688	1	3.95
BAIAP2	10458	brain-specific angiogenesis inhibitor 1-associated protein 2 isoform 3	5453565	LSDSySNTLPVR	Y5(Phospho)	S(2): 0.0; S(4): 1.8; Y(5): 96.5; S(6): 1.8; T(8): 0.0	Y337	Y5;	7	1	QSKLSDSySNTLPVR	2.72	83	1431.65032	0	1.58

PRKCD	5580	protein kinase C delta type	31377782	RSDsASSEPVGIyQGFEK	S4(Phospho); Y13(Phospho)	S(2): 0.0; S(4): 100.0; S(6): 0.0; S(7): 0.0; Y(13): 100.0	S304; Y313	S4; Y13;	14	1	RASRRSDsASSEPVG;SSEPVGIyQGFEKKT	5.14	54	2116.86235	1	6.24
BAIAP2	10458	brain-specific angiogenesis inhibitor 1-associated protein 2 isoform 3	5453565	LSDsYSNTLPVRK	Y5(Phospho)	S(2): 0.0; S(4): 96.0; Y(5): 2.0; S(6): 2.0; T(8): 0.0	S336	S4;	7	1	SQSKLSDsYSNTLPV	3.51	79	1559.74492	1	1.72
TNS3	64759	tensin-3	65288071	ESMCSTPAFPVSPETPyVK	C4(Carbamidomethyl); Y17(Phospho)	S(2): 0.0; S(5): 0.0; T(6): 0.0; S(12): 0.0; T(15): 0.1; Y(17): 99.9	Y855	Y17;	5	1	PVSPETPyVKTALRH	2.93	47	2206.94658	0	1.83

TNS3	64759	tensin-3	65288071	ESMCSTPAFPVSPETPyVK	M3(Oxidation); C4(Carbamidomethyl); Y17(Phospho)	S(2): 0.0; S(5): 0.0; T(6): 0.0; S(12): 0.0; T(15): 2.7; Y(17): 97.3	Y855	Y17;	5	1	PVSPETPyVKTALRH	2.93	55	2222.94048	0	1.89
EGFR	1956	epidermal growth factor receptor isoform a precursor	29725609	GSHQISLDNPDyQQDFFPK	Y12(Phospho)	S(2): 0.0; S(6): 0.0; Y(12): 100.0	Y1172	Y12;	9	1	ISLDNPDyQQDFFPK	4.36	63	2315.99009	0	4.70
EGFR	1956	epidermal growth factor receptor isoform a precursor	29725609	GSHQISLDNPDyQQDFFPK	Y12(Phospho)	S(2): 0.0; S(6): 0.0; Y(12): 100.0	Y1172	Y12;	9	1	ISLDNPDyQQDFFPK	4.36	91	2315.99321	0	3.64

ANXA2	302	annexin A2 isoform 2	209862831	LSLEGDHSTPPSAyGSVK	Y14(Phospho)	S(2): 0.0; S(8): 0.0; T(9): 0.0; S(12): 0.0; Y(14): 100.0; S(16): 0.0	Y24	Y14;	24	1	HSTPPSAyGSVKAYT	5.16	88	1924.8687	0	0.81
ANXA2	302	annexin A2 isoform 2	209862831	LSLEGDHSTPPSAyGSVK	Y14(Phospho); K18(Label:13C(6))	S(2): 0.0; S(8): 0.0; T(9): 0.0; S(12): 0.1; Y(14): 97.5; S(16): 2.4	Y24	Y14;	24	1	HSTPPSAyGSVKAYT	5.16	47	1930.88431	0	0.80
PTRF	284119	polymerase I and transcript release factor	42734430	KSFTPDHVVyAR	Y10(Phospho)	S(2): 0.0; T(4): 0.0; Y(10): 100.0	Y308	Y10;	2	1	FTPDHVVyARSKTAV	4.00	39	1499.70288	1	4.83
PPP1CA	5499	serine/threonine-protein phosphatase PP1-alpha catalyt	45827798	QSLETICLLLAyKIK	C7(Carbamidomethyl); Y12(Phospho)	S(2): 0.0; T(5): 0.2; Y(12): 99.8	Y66	Y12;	1	1	TICLLLAyKIKYPEN	2.26		1872.9699	1	2.27

		ic subunit isoform 2														
EVPL	2125	envoplakin	156104874	ISILEPETGKDMSPyEAYKR	Y15(Phospho)	S(2): 0.0; T(8): 0.0; S(13): 3.6; Y(15): 92.8; Y(18): 3.6	Y1700	Y15;	10	1	TGKDMSPyEAYKRGI	4.67	46	2407.11893	2	9.95
TNS3	64759	tensin-3	65288071	LSLGQyDNDAGGQLPFSK	Y6(Phospho); K18(Label:13C(6))	S(2): 0.0; Y(6): 100.0; S(17): 0.0	Y780	Y6;	4	1	RKLSLGQyDNDAGGQ	3.27	101	1995.91179	0	1.66
GPRC5C	55890	G-protein coupled receptor family C group 5 member C isoform b precursor	40217833	NSQVFRNPyVWD	R6(Label:13C(6)); Y9(Phospho)	S(2): 0.0; Y(9): 100.0	Y450	Y9;	2	1	SQVFRNPyVWD_	2.46	35	1610.71184	1	0.25

KRT7	3855	keratin, type II cytoskeletal 7	67782365	LSSARPGGLGSSSLyGLGASRPR	S3(Phospho); Y15(Phospho)	S(2): 0.1; S(3): 1.2; S(11): 32.9; S(12): 32.9; S(13): 32.9; Y(15): 100.0; S(20): 0.0	Y40	Y15;	31	1	GLGSSSLyGLGASRP	3.55	34	2406.13199	0	1.45
PRKCD	5580	protein kinase C delta type	31377782	RSDSASsEPVGIyQGFEKK	S4(Phospho); Y13(Phospho)	S(2): 0.1; S(4): 2.2; S(6): 2.2; S(7): 95.5; Y(13): 100.0	S307; Y313	S7; Y13;	18	1	RRSDSASsEPVGIYQ;SSEPVGIyQGFEKKT	5.12	67	2244.95513	2	7.10
PRKCD	5580	protein kinase C delta type	31377782	RSDSASSEPVGIyQGFEKK	S4(Phospho); Y13(Phospho)	S(2): 1.0; S(4): 33.0; S(6): 33.0; S(7): 33.0; Y(13): 100.0	Y313	Y13;	18	1	SSEPVGIyQGFEKKT	5.12	42	2244.95578	2	8.01
CTTN	2017	src substrate cortactin isoform a	20357552	LPSSPVyEDAASFK	Y7(Phospho)	S(3): 0.0; S(4): 0.0; Y(7): 100.0; S(12): 0.0	Y421	Y7;	6	1	RLPSSPVyEDAASFK	3.28	60	1590.7105	0	1.90
GAB1	2549	GRB2-associated-	46370073	DASSQDCyDIPR	C7(Carbamidomethyl);	S(3): 0.0; S(4): 0.0; Y(8): 100.0	Y406	Y8;	5	1	DASSQDCyDIPRAFP	2.43	74	1506.55742	0	1.44

		binding protein 1 isoform b			Y8(Phospho)											
SPTAN1	6709	spectrin alpha chain, non-erythrocytic 1 isoform 3	306966132	ALSSEGKPyVTKEELYQNLTR	Y9(Phospho)	S(3): 0.0; S(4): 0.0; Y(9): 97.8; T(11): 2.1; Y(16): 0.0; T(20): 0.0	Y2403	Y9;	5	1	LSSEGKPyVTKEELY	2.90		2506.21744	1	1.79
NEDD9	4739	enhancer of filamentation 1 isoform 3	215422375	HQSLSPNHPPPQLGQSVGSQNDAyDVPR	Y24(Phospho)	S(3): 0.0; S(5): 0.0; S(16): 0.0; S(19): 0.0; Y(24): 100.0	Y317	Y24;	9	1	VGSQNDAyDVPRGVQ	4.71	40	3105.41762	0	12.25
NEDD9	4739	enhancer of filamentation 1 isoform 3	215422375	HQSLSPNHPPPQLGQSVGSQNDAyDVPR	Y24(Phospho)	S(3): 0.0; S(5): 0.0; S(16): 0.0; S(19): 0.0; Y(24): 100.0	Y317	Y24;	9	1	VGSQNDAyDVPRGVQ	4.71		3105.41855	0	9.17

TNKS1BP1	85456	182 kDa tankyrase-1-binding protein	110556636	DVSLGTyGSR	Y7(Phospho); R10(Label:13C(6))	S(3): 0.0; T(6): 1.9; Y(7): 96.2; S(9): 1.9	Y940	Y7;	5	1	RDVSLGTyGSRAAEP	1.61	44	1140.50224	0	1.19
TAGLN2	8407	transgelin-2	4507357	GASQAGMTGyGMPR	Y10(Phospho); R14(Label:13C(6))	S(3): 0.0; T(8): 0.1; Y(10): 99.9	Y192	Y10;	10	1	SQAGMTGyGMPRQIL	3.32	53	1469.5999	0	0.43
TAGLN2	8407	transgelin-2	4507357	GASQAGMTGyGMPR	M7(Oxidation); Y10(Phospho); R14(Label:13C(6))	S(3): 0.0; T(8): 2.4; Y(10): 97.6	Y192	Y10;	10	1	SQAGMTGyGMPRQIL	3.32	48	1485.59563	0	0.42
CDKL5	6792	cyclin-dependent kinase-like 5	4507281	NLSEGNNANYTEyVATR	Y13(Phospho)	S(3): 0.0; Y(10): 0.0; T(11): 0.0; Y(13): 100.0; T(16): 0.0	Y171	Y13;	7	1	NNANYTEyVATRWYR	3.04	109	1995.84441	0	1.24
TNS3	64759	tensin-3	65288071	WDSYENLSADGEVLHTQGPVDGSLyA	Y25(Phospho)	S(3): 0.0; Y(4): 0.0; S(8): 0.0; T(16): 0.0; S(23): 0.2; Y(25): 99.8	Y354	Y25;	34	1	GPVDGSLyAKVRKKS	5.52	36	3031.33811	0	4.59

				K													
TNS3	64759	tensin-3	65288071	KLSLGQyDNDAGGQLPFSK	Y7(Phospho)	S(3): 0.0; Y(7): 100.0; S(18): 0.0	Y780	Y7;	10	1	RKLSLGQyDNDAGGQ	4.56	93	2117.99004	1	1.80
SYK	6850	tyrosine-protein kinase SYK isoform 2	205277435	QESTVSFNPyEPELAPWAADKGPQR	Y10(Phospho)	S(3): 0.2; T(4): 3.8; S(6): 3.8; Y(10): 92.2	Y300	Y10;	9	1	STVSFNPyEPELAPW	4.64	40	2897.31296	1	0.84
SHB	6461	SH2 domain-containing adapter protein B	106879210	GESAGyMEPYEAQR	Y6(Phospho); R14(Label:13C(6))	S(3): 1.8; Y(6): 98.2; Y(10): 0.0	Y268	Y6;	5	1	GKGESAGyMEPYEAQ	3.56	70	1673.66069	0	0.29

TNS3	64759	tensin-3	65288071	WDsYENLSADGEVLHTQGPVDGSLyAK	S3(Phospho); Y25(Phospho)	S(3): 96.2; Y(4): 3.7; S(8): 0.2; T(16): 0.0; S(23): 0.2; Y(25): 99.8	S332; Y354	S3; Y25;	34	1	DPLIRWDsYENLSAD;GPVDGSLyAKVRKKS	5.52	55	3111.30742	0	2.99
TNS3	64759	tensin-3	65288071	WDsYENLSADGEVLHTQGPVDGSLyAK	S3(Phospho); Y25(Phospho)	S(3): 99.9; Y(4): 0.1; S(8): 0.0; T(16): 0.0; S(23): 0.1; Y(25): 99.9	S332; Y354	S3; Y25;	34	1	DPLIRWDsYENLSAD;GPVDGSLyAKVRKKS	5.52	49	3111.30686	0	2.93
PKM	5315	pyruvate kinase isozymes M1/M2 isoform a	33286418	AEGSDVANAVLDGADCIMLSGETAKGDyPLEAVR	C16(Carbamidomethyl); Y28(Phospho)	S(4): 0.0; S(20): 6.7; T(23): 6.7; Y(28): 86.7	Y370	Y28;	2	1	GETAKGDyPLEAVRM	4.00		3574.61899	1	0.68
NEDD9	4739	enhancer of filamentation 1 isoform 3	215422375	RHQSLSPNHPPPQLGQSVGSQNDAyDVPR	Y25(Phospho)	S(4): 0.0; S(6): 0.0; S(17): 0.0; S(20): 0.0; Y(25): 100.0	Y317	Y25;	4	1	VGSQNDAyDVPRGVQ	5.70	34	3261.52114	1	10.38
RPS27	6232	40S ribosomal	4506711	LVQSPNSyFMDVK	Y8(Phospho)	S(4): 0.0; S(7): 1.4; Y(8): 98.6	Y31	Y8;	5	1	LVQSPNSyFMDVKC	2.58	72	1607.71599	0	0.79

		protein S27									P					
NCK1	4690	cytoplasmic protein NCK1 isoform 1	5453754	RKPSVPDSASPADDSFVDPGERLyDLNMPAYVK	Y31(Phospho)	S(4): 0.0; S(8): 0.0; S(10): 0.0; S(15): 0.0; Y(24): 95.4; Y(31): 4.6	Y105	Y24;	5	1	VDPGERLyDLNMPAY	5.42	33	3716.7333	2	7.10
PSMB4	5692	proteasome subunit beta type-4	22538467	VNNSTMLGASGDyADFQYLK	Y13(Phospho); K20(Label:13C(6))	S(4): 0.0; T(5): 0.0; S(10): 2.6; Y(13): 97.4; Y(18): 0.0	Y102	Y13;	6	1	MLGASGDyADFQYLK	1.73	54	2280.00054	0	0.50
HIPK1	204851	homeodomain-interacting protein kinase 1 isoform 2	38201638	AVCSTyLQSR	C3(Carbamidomethyl); Y6(Phospho)	S(4): 0.0; T(5): 0.0; Y(6): 100.0; S(9): 0.0	Y352	Y6;	6	1	SKAVCSTyLQSRYYR	2.67	63	1264.53887	0	0.76

CTTN	2017	src substrate cortactin isoform a	20357552	GPVSGTEPEPVYSMEAADyR	Y19(Phospho)	S(4): 0.0; T(6): 0.0; Y(12): 0.0; S(13): 0.0; Y(19): 100.0	Y453	Y19;	45	1	YSMEAADyREASSQQ	4.20	44	2234.93071	0	2.11
CTTN	2017	src substrate cortactin isoform a	20357552	GPVSGTEPEPVySMEAADYR	Y12(Phospho)	S(4): 0.0; T(6): 0.0; Y(12): 98.5; S(13): 1.5; Y(19): 0.0	Y446	Y12;	45	1	GTEPEPVySMEAADY	4.20	79	2234.93144	0	3.63
PKM	5315	pyruvate kinase isozymes M1/M2 isoform a	33286418	LNFSHGTHEyHAETIK	Y10(Phospho); K16(Label:13C(6))	S(4): 0.0; T(7): 0.0; Y(10): 97.5; T(14): 2.5	Y83	Y10;	5	1	FSHGTHEyHAETIKN	5.21	42	1969.88742	0	0.73
MAPK12	6300	PREDICTED: mitogen-activated protein kinase	397138106	QADSEMTGyVVTR	Y9(Phospho); R13(Label:13C(6))	S(4): 0.0; T(7): 0.0; Y(9): 99.9; T(12): 0.1	Y175	Y9;	6	1	ADSEMTGyVVTRWYR	4.28	69	1542.66069	0	2.34

		12																
MAPK12	6300	PREDICTED: mitogen-activated protein kinase 12	397138106	QADSEMTGyVVTR	M6(Oxidation); Y9(Phospho)	S(4): 0.0; T(7): 3.1; Y(9): 96.8; T(12): 0.1	Y175	Y9;	6	1	ADSEMTGyVVTRWYR	4.28	57	1552.63359	0	2.01		
CTTN	2017	src substrate cortactin isoform a	20357552	AELSYRGPVSGTEPEPVySMEAADYR	Y18(Phospho)	S(4): 0.0; Y(5): 0.0; S(10): 0.0; T(12): 0.0; Y(18): 97.9; S(19): 2.1; Y(25): 0.0	Y446	Y18;	13	1	GTEPEPVySMEAADY	3.63	58	2954.28513	1	3.58		
CTTN	2017	src substrate cortactin isoform a	20357552	GPVSGTEPEPVySMEAADYR	Y12(Phospho); M14(Oxidation)	S(4): 0.1; T(6): 2.4; Y(12): 95.0; S(13): 2.4; Y(19): 0.0	Y446	Y12;	45	1	GTEPEPVySMEAADY	4.20	38	2250.92754	0	3.97		

NCK1	4690	cytoplasmic protein NCK1 isoform 1	5453754	RKPsVPDSASPADDSFVDPGERLyDLNMPAYVK	S10(Phospho); S15(Phospho)	S(4): 94.5; S(8): 5.2; S(10): 0.3; S(15): 0.0; Y(24): 94.8; Y(31): 5.2	S85; Y105	S4; Y24;	5	1	GKVKRKPsVPDSASP;VDPGERLyDLNMPAY	5.42		3796.69301	2	11.93
ENO1	2023	alpha-enolase isoform 1	4503571	AAVPSGASTGIyEALELR	Y12(Phospho)	S(5): 0.0; S(8): 0.0; T(9): 0.0; Y(12): 100.0	Y44	Y12;	6	1	SGASTGIyEALELRD	3.93	100	1884.9124	0	0.52
BCAR1	9564	breast cancer anti-estrogen resistance protein 1 isoform 7	282398123	GLPPSNHHAVYDVPPsVSK	Y11(Phospho)	S(5): 0.0; Y(11): 2.3; S(16): 97.7; S(18): 0.1	S309	S16;	10	1	AVYDVPPsVSKDVPD	3.07		2080.9852	0	2.25
NEDD9	4739	enhancer of filamentation 1 isoform 3	215422375	GPVFSVPVGEIKPQGVyDIPPTK	Y17(Phospho)	S(5): 0.0; Y(17): 100.0; T(22): 0.0	Y214	Y17;	29	1	EIKPQGVyDIPPTKG	3.94		2504.28696	0	9.31

BCAR1	9564	breast cancer anti-estrogen resistance protein 1 isoform 7	282398123	GLLPSQYGQEVyDTPPMAVK	Y12(Phospho)	S(5): 0.0; Y(7): 0.0; Y(12): 100.0; T(14): 0.0	Y265	Y12;	12	1	SQYGQEVyDTPPMAV	2.98	63	2273.06108	0	1.93
PKP3	11187	plakophilin-3	6005830	GGVGSRADyDTLSLR	R6(Label:13C(6)); Y9(Phospho); R15(Label:13C(6))	S(5): 0.0; Y(9): 98.4; T(11): 1.6; S(13): 0.0	Y176	Y9;	6	1	GVGSRADyDTLSLRS	3.88	50	1658.7935	1	0.47
PRPF4B	8899	serine/threonine-protein kinase PRP4 homolog	89276756	LCDFGSASHVADNDITPyLVSR	C2(Carbamidomethyl); Y18(Phospho)	S(6): 0.0; S(8): 0.0; T(16): 0.0; Y(18): 97.5; S(21): 2.5	Y849	Y18;	74	1	ADNDITPyLVSRFYR	5.33		2517.10965	0	1.64

CTTN	2017	src substrate cortactin isoform a	20357552	MDKNASTFEDVTQVSSAyQK	Y18(Phospho)	S(6): 0.0; T(7): 0.0; T(12): 0.0; S(15): 0.0; S(16): 0.0; Y(18): 100.0	Y334	Y18;	10	1	VTQVSSAyQKTVPVE	2.89	36	2329.00467	1	2.36
CTTN	2017	src substrate cortactin isoform a	20357552	MDKNASTFEDVTQVSSAyQK	Y18(Phospho)	S(6): 0.0; T(7): 0.0; T(12): 0.0; S(15): 0.0; S(16): 0.1; Y(18): 99.9	Y334	Y18;	10	1	VTQVSSAyQKTVPVE	2.89	44	2329.00786	1	2.02
GPRC5A	9052	retinoic acid-induced protein 3	4506403	AHAWPSPYKDyEVKK	K9(Label:13C(6)); Y11(Phospho); K14(Label:13C(6)); K15(Label:13C(6))	S(6): 0.0; Y(8): 0.0; Y(11): 100.0	Y350	Y11;	60	1	WPSPYKDyEVKKEGS	4.10	51	1916.9412	2	15.72
GPRC5A	9052	retinoic acid-induced protein 3	4506403	AHAWPSPYKDyEVKK	Y11(Phospho)	S(6): 0.0; Y(8): 0.0; Y(11): 100.0	Y350	Y11;	60	1	WPSPYKDyEVKKEGS	4.10		1898.8843	2	16.03

GPRC5A	9052	retinoic acid-induced protein 3	4506403	AHAWPSPyKDYEVKK	Y8(Phospho)	S(6): 0.0; Y(8): 100.0; Y(11): 0.0	Y347	Y8;	60	1	AHAWPSPyKDYEVKK	4.10	37	1898.88286	2	21.05
GPRC5A	9052	retinoic acid-induced protein 3	4506403	AHAWPSPyKDYEVK	Y8(Phospho)	S(6): 0.0; Y(8): 100.0; Y(11): 0.0	Y347	Y8;	43	1	AHAWPSPyKDYEVKK	4.58	43	1770.78879	1	14.23
GPRC5A	9052	retinoic acid-induced protein 3	4506403	AHAWPSPyKDYEVK	Y8(Phospho); K9(Label:13C(6)); K14(Label:13C(6))	S(6): 0.0; Y(8): 100.0; Y(11): 0.0	Y347	Y8;	43	1	AHAWPSPyKDYEVKK	4.58	54	1782.82732	1	14.92
GPRC5A	9052	retinoic acid-induced protein 3	4506403	AHAWPSPyKDyEVKK	Y8(Phospho); Y11(Phospho)	S(6): 0.0; Y(8): 100.0; Y(11): 100.0	Y347; Y350	Y8; Y11;	60	1	AHAWPSPyKDYEVKK;WPSPYKDyEVKKEGS	4.10	51	1978.85039	2	35.37
GPRC5A	9052	retinoic acid-induced protei	4506403	AHAWPSPyKDYEVKK	Y8(Phospho)	S(6): 0.0; Y(8): 98.3; Y(11): 1.7	Y347	Y8;	60	1	AHAWPSPyKDYEVKK	4.10		1898.88388	2	11.64

		n 3														
DAPP 1	27071	dual adapter for phosphotyrosine and 3-phosphotyrosine and 3-phosphoinositide	158631203	KVEEPSIyESVR	Y8(Phospho)	S(6): 0.0; Y(8): 98.4; S(10): 1.6	Y139	Y8;	5	1	KVEEPSIyESVRVHT	3.67	57	1515.71025	1	0.46
C6orf 132	647024	uncharacterized protein C6orf 132	256574805	HKAPGSADyGFAPAAGR	Y9(Phospho)	S(6): 0.0; Y(9): 100.0	Y1142	Y9;	8	1	KAPGSADyGFAPAAG	4.79	47	1752.78458	1	11.63
C6orf 132	647024	uncharacterized protein C6orf 132	256574805	HKAPGSADyGFAPAAGR	Y9(Phospho)	S(6): 0.0; Y(9): 100.0	Y1142	Y9;	8	1	KAPGSADyGFAPAAG	4.79	86	1752.78594	1	11.56

SHB	6461	SH2 domain-containing adapter protein B	106879210	AGKGESAGyMEPYEAQR	Y9(Phospho)	S(6): 0.0; Y(9): 100.0; Y(13): 0.0	Y268	Y9;	15	1	GKGESAGyMEPYEAQ	5.94	85	1923.79314	1	0.25
SHB	6461	SH2 domain-containing adapter protein B	106879210	AGKGESAGyMEPYEAQR	Y9(Phospho)	S(6): 0.0; Y(9): 100.0; Y(13): 0.0	Y268	Y9;	15	1	GKGESAGyMEPYEAQ	5.94	52	1923.79447	1	0.24
GPRC5A	9052	retinoic acid-induced protein 3	4506403	AHAWPSPyKDyEVKK	Y8(Phospho); Y11(Phospho)	S(6): 0.1; Y(8): 99.9; Y(11): 100.0	Y347; Y350	Y8; Y11;	60	1	AHAWPSPyKDYEVKK;WPSPYKDyEVKKEGS	4.10		1978.84842	2	31.79
GSK3B	2932	glycogen synthase kinase-3 beta	225903437	GEPNVSyICSR	Y7(Phospho); C9(Carbamidomethyl)	S(6): 1.8; Y(7): 98.2; S(10): 0.0	Y216	Y7;	40	1	RGEPNVSyICSRYYR	2.56	62	1361.55461	0	1.05

		isoform 2														
GPRC5A	9052	retinoic acid-induced protein 3	4506403	AHAWPsPYKDyEVK	S6(Phospho); Y8(Phospho)	S(6): 100.0; Y(8): 2.2; Y(11): 97.8	S345; Y350	S6; Y11;	43	1	PRAHAWPsPYKDYEV;WPSPYKDyEVKKEGS	4.58		1850.7526	1	10.43
SHB	6461	SH2 domain-containing adapter protein B	106879210	AGKGESAGyMEPYEAQR	K3(Label:13C(6)); Y9(Phospho); M10(Oxidation); R17(Label:13C(6))	S(6): 2.1; Y(9): 97.9; Y(13): 0.0	Y268	Y9;	15	1	GKGESAGyMEPYEAQ	5.94	39	1951.82938	1	0.25
SHB	6461	SH2 domain-containing adapter protein B	106879210	AGKGESAGyMEPYEAQR	K3(Label:13C(6)); Y9(Phospho); M10(Oxidation); R17(Label:13C	S(6): 2.5; Y(9): 94.9; Y(13): 2.5	Y268	Y9;	15	1	GKGESAGyMEPYEAQ	5.94	31	1951.82854	1	0.23

					(6))											
PRPF 4B	8899	serine/ threonine-protein kinase PRP4 homolog	89276756	LCDF GsASH VADN DITPy LVSR	C2(Carbamidomethyl); S8(Phospho); S21(Phospho); R22(Label:13C(6))	S(6): 96.6; S(8): 3.4; T(16): 0.0; Y(18): 96.6; S(21): 3.4	S837; Y849	S6; Y18;	74	1	LKLC DFGsA SHVA DN;AD NDITP yLVSR FYR	5.33		2603. 09635	0	0.69
GPRC 5A	9052	retinoic acid-induced protein 3	4506403	AHAW PsPYK DyEV KK	Y8(Phospho); Y11(Phospho)	S(6): 96.9; Y(8): 3.1; Y(11): 100.0	S345; Y350	S6; Y11;	60	1	PRAH AWPsP YKDY EV;WP SPYK DyEV KKEG S	4.10		1978. 84714	2	5.60
GPRC 5A	9052	retinoic acid-induced protei	4506403	AHAW PsPYK DYEV K	S6(Phospho); Y8(Phospho)	S(6): 99.9; Y(8): 50.0; Y(11): 50.0	S345	S6;	43	1	PRAH AWPsP YKDY EV	4.58		1850. 75517	1	6.81

		n 3														
ELMO2	63916	engulfment and cell motility protein 2	33469947	EVCDGWSLPNPEyYTLR	C3(Carbamidomethyl); Y13(Phospho)	S(7): 0.0; Y(13): 95.1; Y(14): 2.4; T(15): 2.4	Y48	Y13;	11	1	WSLPNPEyYTLRYAD	1.97		2178.92534	0	7.13
SHC1	6464	SHC-transforming protein 1 isoform 4	194239668	ELFDDPSyVNVQNLDK	Y8(Phospho); K16(Label:13C(6))	S(7): 2.1; Y(8): 97.9	Y317	Y8;	12	1	ELFDDPSyVNVQNLD	3.91	72	1981.88921	0	2.52
SHC1	6464	SHC-transforming protein 1 isoform 4	194239668	ELFDDPSyVNVQNLDKAR	Y8(Phospho)	S(7): 2.3; Y(8): 97.7	Y317	Y8;	4	1	ELFDDPSyVNVQNLD	2.79	49	2202.99881	1	2.21
SHC1	6464	SHC-transforming protein 1	194239668	ELFDDPsYVNVQNLDK	S7(Phospho)	S(7): 98.1; Y(8): 1.9	S316	S7;	12	1	RELFDDPsYVNVQNL	3.91	34	1975.86765	0	2.30

		isoform 4														
PXN	5829	paxillin isoform 1	170932516	FIHQQPQSSSPVYGSsAK	S16(Phospho)	S(8): 0.0; S(9): 0.0; S(10): 0.0; Y(13): 2.2; S(15): 2.2; S(16): 95.7	S91	S16;	24	1	SSPVYGSsAKTSSVS	4.58	42	2027.9249	0	2.89
PXN	5829	paxillin isoform 1	170932516	FIHQQPQSSsPVyGSSAK	S10(Phospho); S15(Phospho)	S(8): 0.0; S(9): 0.0; S(10): 100.0; Y(13): 96.6; S(15): 3.3; S(16): 0.1	S85; Y88	S10; Y13;	24	1	HQQPQSSsPVYGSSA;PQSSSPVyGSSAKTS	4.58		2107.88523	0	5.82
CTNND1	1500	catenin delta-1 isoform 2A	332688222	LNGPQDHSHLLySTIPR	Y12(Phospho)	S(8): 0.0; Y(12): 93.0; S(13): 3.5; T(14): 3.5	Y42	Y12;	5	1	QDHSHLLySTIPRMQ	4.03		2027.97141	0	1.05
PXN	5829	paxillin isoform 1	170932516	FIHQQPQSSsPVyGSSAK	S10(Phospho); Y13(Phospho)	S(8): 0.1; S(9): 0.1; S(10): 99.8; Y(13): 99.9; S(15): 0.1; S(16): 0.0	S85; Y88	S10; Y13;	24	1	HQQPQSSsPVYGSSA;PQSSSPVyGSSAKTS	4.58	40	2107.89043	0	5.43

PRKCD	5580	protein kinase C delta type	31377782	TGVAGEDMQDNSGTyGK	Y15(Phospho)	T(1): 0.0; S(12): 0.0; T(14): 1.8; Y(15): 98.2	Y334	Y15;	5	1	MQDNSGTyGKIWEGS	3.85	82	1809.69988	0	3.86
HIPK3	10114	homeodomain-interacting protein kinase 3 isoform 2	114796630	TVCSTyLQSR	C3(Carbamidomethyl); Y6(Phospho)	T(1): 0.0; S(4): 0.0; T(5): 1.1; Y(6): 98.9; S(9): 0.0	Y359	Y6;	7	1	SKTVCSTyLQSRYYR	2.84	53	1294.55242	0	5.15
EFNB2	1948	ephrin-B2 precursor	4758250	TADSVFCPHyEK	C7(Carbamidomethyl); Y10(Phospho); K12(Label:13C(6))	T(1): 0.0; S(4): 0.0; Y(10): 100.0	Y304	Y10;	3	1	DSVFCPHyEKVSGDY	3.21	69	1539.62749	0	0.09

PTK2	5747	focal adhesion kinase 1 isoform a	24476013	THAVSVSETDDyAEIIDEEDTYTMPSTR	Y12(Phospho)	T(1): 0.0; S(5): 0.0; S(7): 2.0; T(9): 0.0; Y(12): 97.9; T(21): 0.0; Y(22): 0.0; T(23): 0.0; S(26): 0.0; T(27): 0.0	Y397	Y12;	28	1	SVSETDDyAEIIDEE	4.23	83	3255.35703	0	1.50
TLN1	7094	talin-1	223029410	TMQFEPSTMVyDACR	M2(Oxidation); Y11(Phospho); C14(Carbamidomethyl)	T(1): 0.0; S(7): 0.0; T(8): 0.0; Y(11): 100.0	Y26	Y11;	10	1	FEPSTMVyDACRIIR	4.07	46	1931.73796	0	3.09
TLN1	7094	talin-1	223029410	TMQFEPSTMVyDACR	Y11(Phospho); C14(Carbamidomethyl)	T(1): 0.0; S(7): 0.0; T(8): 0.0; Y(11): 100.0	Y26	Y11;	10	1	FEPSTMVyDACRIIR	4.07	70	1915.7387	0	3.54

CTTN	2017	src substrate cortactin isoform a	20357552	TQTPPVSPAPQPTEERLPSSPVyEDAASFK	Y23(Phospho)	T(1): 0.0; T(3): 0.0; S(7): 0.0; T(13): 0.2; S(19): 4.3; S(20): 4.3; Y(23): 91.3; S(28): 0.0	Y421	Y23;	8	1	RLPSSPVyEDAASFK	3.83		3306.55625	1	1.54
MAPK9	5601	mitogen-activated protein kinase 9 isoform JNK2 gamma	205277412	TACTNFMMTPYVVtR	C3(Carbamidomethyl); Y11(Phospho); R15(Label:13C(6))	T(1): 0.0; T(4): 0.0; T(9): 0.0; Y(11): 0.1; T(14): 99.9	T188	T14;	12	1	MMTPYVVtRYYRAPE	3.96	32	1877.80991	0	0.15
MAPK9	5601	mitogen-activated protein kinase 9 isoform JNK2	205277412	TACTNFMMTPyVVTR	C3(Carbamidomethyl); M7(Oxidation); Y11(Phospho); R15(Label:13C(6))	T(1): 0.0; T(4): 0.0; T(9): 0.1; Y(11): 99.9; T(14): 0.1	Y185	Y11;	12	1	TNFMMTPyVVTRYYR	3.96	56	1893.80657	0	0.16

		gamma															
MAPK9	5601	mitogen-activated protein kinase 9 isoform JNK2 gamma	205277412	TACTNFMMtPyVVTR	C3(Carbamidomethyl); M7(Oxidation); T9(Phospho); Y11(Phospho); R15(Label:13C(6))	T(1): 0.0; T(4): 0.0; T(9): 100.0; Y(11): 100.0; T(14): 0.0	T183; Y185	T9; Y11;	12	1	ACTNFMMtPYVVTRY;TNFMMTPyVVTRYYR	3.96	76	1973.77153	0	0.12
MAPK9	5601	mitogen-activated protein kinase 9 isoform JNK2	205277412	TACTNFMMtPyVVTR	C3(Carbamidomethyl); T9(Phospho); Y11(Phospho); R15(Label:13C(6))	T(1): 0.0; T(4): 0.0; T(9): 100.0; Y(11): 100.0; T(14): 0.0	T183; Y185	T9; Y11;	12	1	ACTNFMMtPYVVTRY;TNFMMTPyVVTRYYR	3.96	81	1957.76775	0	0.12

		gamma															
MAPK9	5601	mitogen-activated protein kinase 9 isoform JNK2 gamma	205277412	TACTNFMMTPyVVTR	C3(Carbamidomethyl); M7(Oxidation); M8(Oxidation); Y11(Phospho); R15(Label:13C(6))	T(1): 0.0; T(4): 0.0; T(9): 2.4; Y(11): 97.5; T(14): 0.1	Y185	Y11;	12	1	TNFMMTPyVVTRYYR	3.96	51	1909.80058	0	0.19
MAPK9	5601	mitogen-activated protein kinase 9 isoform JNK2	205277412	TACTNFMMTPyVVTR	C3(Carbamidomethyl); M8(Oxidation); Y11(Phospho); R15(Label:13C(6))	T(1): 0.0; T(4): 0.0; T(9): 2.4; Y(11): 97.6; T(14): 0.0	Y185	Y11;	12	1	TNFMMTPyVVTRYYR	3.96	65	1893.80657	0	0.17

		gamma														
EPHA2	1969	ephrin type-A receptor 2 precursor	32967311	TYVDPHTyEDPNQAVLK	Y8(Phospho); K17(Label:13C(6))	T(1): 0.0; Y(2): 0.0; T(7): 2.0; Y(8): 98.0	Y594	Y8;	14	1	TYVDPHTyEDPNQAV	2.99	43	2075.94101	0	0.02
NEDD9	4739	enhancer of filamentation 1 isoform 3	215422375	TGHGYVyEYPSR	Y7(Phospho)	T(1): 0.0; Y(5): 0.0; Y(7): 100.0; Y(9): 0.0; S(11): 0.0	Y166	Y7;	21	1	RTGHGYVyEYPSRYQ	4.03	56	1508.61785	0	15.84
NEDD9	4739	enhancer of filamentation 1 isoform 3	215422375	TGHGYVyEYPSR	Y7(Phospho)	T(1): 0.0; Y(5): 0.0; Y(7): 100.0; Y(9): 0.0; S(11): 0.0	Y166	Y7;	21	1	RTGHGYVyEYPSRYQ	4.03	44	1508.6203	0	12.77

SGK223	157285	tyrosine-protein kinase SgK223	157909822	TIyLSSPDSAVGVQWPR	Y3(Phospho); R17(Label:13C(6))	T(1): 2.5; Y(3): 97.4; S(5): 0.1; S(6): 0.0; S(9): 0.0	Y487	Y3;	6	1	EEDHRTIyLSSPDSA	1.82	36	1961.94353	0	0.25
EPHA4	2043	ephrin type-A receptor 4 precursor	4758280	TYVDPFTyEDPNQAVR	Y2(Phospho); Y8(Phospho)	T(1): 50.0; Y(2): 50.0; T(7): 2.3; Y(8): 97.7	Y602	Y8;	6	1	TYVDPFTyEDPNQAV	4.08	57	2074.81767	0	40.00
BCAR1	9564	breast cancer anti-estrogen resistance protein 1 isoform 7	282398123	DVPDGPLLREEtYDVPPAFAK	R9(Label:13C(6)); T12(Phospho); K21(Label:13C(6))	T(12): 97.8; Y(13): 2.2	T324	T12;	4	1	GPLLREEtYDVPPAF	3.30	50	2421.18033	1	1.87
STAT1	6772	signal transducer and activator of transcription	21536301	EAPEPMELDGPKGTGyIK	K12(Label:13C(6)); Y16(Phospho); K18(Label:13C(6))	T(14): 1.6; Y(16): 98.4	Y701	Y16;	6	1	DGPKGTGyIKTELIS	2.78	55	2023.94853	1	0.27

		1-alpha/beta isoform beta														
CFL1	1072	cofilin-1	5031635	NIILEEGKEILVGDVGQTVDDPyATFVK	Y23(Phospho)	T(18): 0.0; Y(23): 97.6; T(25): 2.4	Y68	Y23;	5	1	GQTVDDPyATFVKML	3.44	52	3142.56296	1	0.55
MAPK14	1432	mitogen-activated protein kinase 14 isoform 3	20986514	HTDDEMTGyVATR	Y9(Phospho)	T(2): 0.0; T(7): 0.0; Y(9): 99.9; T(12): 0.1	Y182	Y9;	24	1	TDDEMTGyVATRWYR	5.29	49	1575.61466	0	7.36
MAPK14	1432	mitogen-activated protein kinase 14 isoform 3	20986514	HTDDEMTGyVATR	Y9(Phospho); R13(Label:13C(6))	T(2): 0.0; T(7): 1.3; Y(9): 98.7; T(12): 0.0	Y182	Y9;	24	1	TDDEMTGyVATRWYR	5.29	92	1581.63323	0	6.84

MAPK14	1432	mitogen-activated protein kinase 14 isoform 3	20986514	HTDDEMtGyVATR	T7(Phospho); Y9(Phospho); R13(Label:13C(6))	T(2): 0.0; T(7): 100.0; Y(9): 100.0; T(12): 0.0	T180; Y182	T7; Y9;	24	1	RHTDDEMtGYVATRW;TDDEMTGyVATRWYR	5.29	67	1661.60198	0	2.52
MUC1	4582	mucin-1 isoform 15 precursor	324120968	DTYHPMSEYPTyHTHGR	Y12(Phospho); R17(Label:13C(6))	T(2): 0.0; Y(3): 0.0; S(7): 0.0; Y(9): 0.0; T(11): 0.0; Y(12): 100.0; T(14): 0.0	Y198	Y12;	10	1	PMSEYPTyHTHGRYV	3.50	29	2177.88279	0	0.19
MUC1	4582	mucin-1 isoform 15 precursor	324120968	DTYHPMSEyPTyHTHGR	Y9(Phospho); Y12(Phospho); R17(Label:13C(6))	T(2): 0.0; Y(3): 0.0; S(7): 2.6; Y(9): 97.4; T(11): 2.6; Y(12): 97.4; T(14): 0.0	Y195; Y198	Y9; Y12;	10	1	TYHPMSEyPTYHTHG;PMSEYPTyHTHGRYV	3.50	29	2257.85044	0	0.30
SHB	6461	SH2 domain-containing adapte	106879210	VTIADDySDPFDAK	Y7(Phospho); K14(Label:13C(6))	T(2): 0.0; Y(7): 97.9; S(8): 2.1	Y246	Y7;	5	1	KVTIADDySDPFDAK	2.91	57	1642.69707	0	0.22

		r protei n B														
YES1	7525	tyrosi ne-protei n kinase Yes	4885661	WTAP EAALy GR	Y9(Pho spho); R11(La bel:13C (6))	T(2): 0.0; Y(9): 100.0	Y446	Y9;	2	1	TAPEA ALyGR FTIKS	2.95	63	1320. 61443	0	1.00
EPHA 1	2041	ephrin type-A recept or 1 precur sor	22131665 0	ATDV DRED KLWL KPyVD LQAY EDPA QGAL DFTR	Y15(Ph ospho)	T(2): 0.3; Y(15): 99.7; Y(21): 0.0; T(32): 0.0	Y599	Y15;	5	1	DKLW LKPyV DLQA YE	4.14		3918. 86098	2	15.83
WAS L	8976	neural Wisko tt-Aldric h syndr ome protei n	51702526	ETSKV IyDFIE K	Y7(Pho spho)	T(2): 2.1; S(3): 2.1; Y(7): 95.8	Y256	Y7;	5	1	RETSK VIyDFI EKTG	2.60	30	1551. 7354	1	2.20
PKM	5315	pyruv ate kinase isozy mes M1/M	33286418	ITLDN AyME KCDE NILWL DYK	Y7(Pho spho); C11(Ca rbamid omethy l)	T(2): 3.1; Y(7): 96.9; Y(20): 0.0	Y148	Y7;	3	1	KITLD NAyM EKCD EN	2.53		2727. 2078	1	0.80

		2 isoform a														
PARD3	56288	partitioning defective 3 homolog isoform 7	296278198	DVTIGGSAPIyVK	Y11(Phospho); K13(Label:13C(6))	T(3): 0.0; S(7): 0.0; Y(11): 100.0	Y445	Y11;	7	1	IGGSAPIyVKNILPR	2.27	57	1405.70683	0	0.10
ITGB1	3688	integrin beta-1 isoform 1A precursor	19743823	WDTGENPIyK	Y9(Phospho); K10(Label:13C(6))	T(3): 0.0; Y(9): 100.0	Y783	Y9;	2	1	DTGENPIyKSAVTTV	2.83	51	1308.55998	0	0.67
ITGB1	3688	integrin beta-1 isoform 1A precursor	19743823	WDTGENPIyKSAVTTVVNPK	Y9(Phospho)	T(3): 0.0; Y(9): 100.0; S(11): 0.0; T(14): 0.0; T(15): 0.0	Y783	Y9;	7	1	DTGENPIyKSAVTTV	3.71	40	2299.10063	1	0.74
SGK223	157285	tyrosine-protein kinase SgK223	157909822	EATQPEPIyAESTKR	Y9(Phospho)	T(3): 0.0; Y(9): 100.0; S(12): 0.0; T(13): 0.0	Y411	Y9;	6	1	ATQPEPIyAESTKRK	2.57	40	1799.82207	1	0.09

ITGB1	3688	integrin beta-1 isoform 1A precursor	19743823	WDTGENPIyKSAVTTVVNPK	K10(Label:13C(6)); S11(Phospho); K20(Label:13C(6))	T(3): 0.0; Y(9): 98.2; S(11): 1.8; T(14): 0.0; T(15): 0.0	Y783	Y9;	7	1	DTGENPIyKSAVTTV	3.71	53	2311.14145	1	0.59
SGK223	157285	tyrosine-protein kinase SgK223	157909822	EATQPEPIyAESTK	Y9(Phospho); K14(Label:13C(6))	T(3): 0.0; Y(9): 99.8; S(12): 0.1; T(13): 0.1	Y411	Y9;	7	1	ATQPEPIyAESTKRK	2.72	31	1649.7404	0	0.11
MAPK7	5598	mitogen-activated protein kinase 7 isoform 2	20986499	GLCTSPAEHQYFMTEyVATR	C3(Carbamidomethyl); Y16(Phospho); R20(Label:13C(6))	T(4): 0.0; S(5): 0.0; Y(11): 0.0; T(14): 1.9; Y(16): 96.1; T(19): 1.9	Y82	Y16;	10	1	HQYFMTEyVATRWYR	3.61	40	2447.05034	0	0.21
WASL	8976	neural Wiskott-Aldrich syndrome protei	51702526	DRETSKVIyDFIEK	Y9(Phospho)	T(4): 0.0; S(5): 0.0; Y(9): 100.0	Y256	Y9;	2	1	RETSKVIyDFIEKTG	3.56		1822.85666	2	2.51

		n														
BCAR3	8412	breast cancer anti-estrogen resistance protein 3 isoform 1	387157906	HGETFTFRDPHLLDPTVEyVK	Y19(Phospho)	T(4): 0.0; T(6): 0.0; T(16): 0.0; Y(19): 100.0	Y117	Y19;	12	1	LLDPTVEyVKFSKER	4.55	36	2581.20334	1	1.91
BCAR3	8412	breast cancer anti-estrogen resistance protein 3 isoform 1	387157906	HGETFTFRDPHLLDPTVEyVKFSK	Y19(Phospho)	T(4): 0.0; T(6): 0.0; T(16): 0.0; Y(19): 96.9; S(23): 3.1	Y117	Y19;	21	1	LLDPTVEyVKFSKER	4.09		2943.41201	2	3.83
BCAR3	8412	breast cancer anti-estrogen resistance protei	387157906	HGETFTFRDPHLLDPTVEyVKFSK	Y19(Phospho)	T(4): 0.0; T(6): 0.0; T(16): 2.6; Y(19): 97.4; S(23): 0.0	Y117	Y19;	21	1	LLDPTVEyVKFSKER	4.09		2943.4094	2	3.55

		n 3 isoform 1																
BCAR3	8412	breast cancer anti-estrogen resistance protein 3 isoform 1	387157906	HGETFTFRDPHLLDPTVEyVK	Y19(Phospho)	T(4): 0.0; T(6): 0.0; T(16): 2.7; Y(19): 97.3	Y117	Y19;	12	1	LLDPTVEyVKFSKER	4.55		2581.20498	1	4.54		
PTPN11	5781	tyrosine-protein phosphatase non-receptor type 11 isoform 1	33356177	IQNTGDyYDLYGGEK	Y7(Phospho)	T(4): 0.0; Y(7): 98.2; Y(8): 1.8; Y(11): 0.0	Y62	Y7;	6	1	KIQNTGDyYDLYGE	3.75	76	1815.74773	0	3.80		

PABPC1	26986	polyadenylate-binding protein 1	46367787	IVATKPLyVALAQR	K5(Label:13C(6)); Y8(Phospho); R14(Label:13C(6))	T(4): 0.0; Y(8): 100.0	Y364	Y8;	2	1	IVATKPLyVALAQRK	3.06	56	1634.94182	0	1.01
SHB	6461	SH2 domain-containing adapter protein B	106879210	DKVTIADDySDPFDAKNDLK	Y9(Phospho)	T(4): 0.0; Y(9): 97.6; S(10): 2.4	Y246	Y9;	11	1	KVTIADDySDPFDAK	4.43	36	2350.04422	2	0.25
SHB	6461	SH2 domain-containing adapter protein B	106879210	DKVTIADDySDPFDAK	K2(Label:13C(6)); Y9(Phospho); K16(Label:13C(6))	T(4): 0.0; Y(9): 98.0; S(10): 1.9	Y246	Y9;	10	1	KVTIADDySDPFDAK	4.28	53	1891.83908	1	0.24
SHB	6461	SH2 domain-containing adapter	106879210	DKVTIADDySDPFDAK	Y9(Phospho)	T(4): 0.0; Y(9): 98.4; S(10): 1.6	Y246	Y9;	10	1	KVTIADDySDPFDAK	4.28	83	1879.8029	1	0.22

		protein B																
IQGAP1	8826	ras GTPase-activating-like protein IQGAP1	4506787	LQQTyAALNSK	Y5(Phospho)	T(4): 2.0; Y(5): 98.0; S(10): 0.0	Y1510	Y5;	4	1	LVKLQQTyAALNSKA	2.48	50	1316.62273	0	3.75
CDK1	983	cyclin-dependent kinase 1 isoform 2	16306492	IGEGTYGVVyKGR	Y10(Phospho)	T(5): 0.0; Y(6): 0.0; Y(10): 100.0	Y19	Y10;	15	1	EGTYGVVyKGRHKTT	3.74	46	1478.70362	1	2.04
CDK1	983	cyclin-dependent kinase 1 isoform 2	16306492	IGEGTYGVVyKGR	Y10(Phospho)	T(5): 0.0; Y(6): 0.0; Y(10): 100.0	Y19	Y10;	15	1	EGTYGVVyKGRHKTT	3.74	81	1478.70439	1	1.82

IL18	3606	interleukin-18 isoform 1 proprotein	4504653	FIDNTLYFIAEDDENLESDyFGKLESK	Y20(Phospho)	T(5): 0.0; Y(7): 0.0; S(18): 2.5; Y(20): 97.5; S(26): 0.0	Y37	Y20;	7	1	DENLESDyFGKLESK	3.90	52	3295.46445	1	5.38
CDK5	1020	cyclin-dependent kinase 5 isoform 2	256542306	IGEGTyGTVFK	Y6(Phospho); K11(Label:13C(6))	T(5): 1.4; Y(6): 98.6; T(8): 0.0	Y15	Y6;	5	1	EKIGEGTyGTVFKAK	2.72	60	1257.58391	0	1.33
CDK1	983	cyclin-dependent kinase 1 isoform 2	16306492	IGEGtyGVVYKGR	T5(Phospho); Y6(Phospho)	T(5): 100.0; Y(6): 100.0; Y(10): 0.0	T14; Y15	T5; Y6;	15	1	IEKIGEGtYGVVYKG;EKIGEGTyGVVYKGR	3.74	70	1558.66948	1	1.00
BCAR1	9564	breast cancer anti-estrogen resistance protein 1 isofor	282398123	RPGPGTLyDVPR	Y8(Phospho)	T(6): 0.0; Y(8): 100.0	Y385	Y8;	7	1	RPGPGTLyDVPRERV	3.00	29	1407.67618	0	1.79

		m 7														
ANP32A	8125	acidic leucine-rich nuclear phosphoprotein 32 family member A	5453880	LLPQLTYLDGyDR	Y11(Phospho)	T(6): 0.1; Y(7): 2.3; Y(11): 97.6	Y148	Y11;	4	1	QLTYLDGyDRDDKEA	1.98	43	1646.78301	0	0.40
MAPK13	5603	mitogen-activated protein kinase 13	4506085	HADAEMTGyVVTR	Y9(Phospho); R13(Label:13C(6))	T(7): 0.0; Y(9): 100.0; T(12): 0.0	Y182	Y9;	121	1	ADAEMTGyVVTRWYR	4.95	87	1535.66411	0	4.28
MAPK13	5603	mitogen-activated protein kinase 13	4506085	HADAEMTGyVVTR	M6(Oxidation); Y9(Phospho)	T(7): 0.1; Y(9): 99.9; T(12): 0.0	Y182	Y9;	121	1	ADAEMTGyVVTRWYR	4.95	44	1545.63901	0	5.04

MAPK13	5603	mitogen-activated protein kinase 13	4506085	HADAEMTGyVVTR	M6(Oxidation); Y9(Phospho); R13(Label:13C(6))	T(7): 2.0; Y(9): 96.0; T(12): 2.0	Y182	Y9;	121	1	ADAEMTGyVVTRWYR	4.95	70	1551.65996	0	4.75
ERBB2	2064	receptor tyrosine-protein kinase erbB-2 isoform b	54792098	LLDIDETEyHADGGKVPIK	Y9(Phospho)	T(7): 2.6; Y(9): 97.4	Y847	Y9;	4	1	LDIDETEyHADGGKV	3.63		2193.05802	1	1.95
EPHA1	2041	ephrin type-A receptor 1 precursor	221316650	LLDDFDGTyETQGGKIPIR	Y9(Phospho)	T(8): 0.0; Y(9): 100.0; T(11): 0.0	Y781	Y9;	15	1	LDDFDGTyETQGGKI	4.49	60	2218.04038	1	10.75
CDK2	1017	cyclin-dependent kinase 2 isofor	166362719	VEKIGEGTyGVVYK	Y9(Phospho)	T(8): 0.0; Y(9): 100.0; Y(13): 0.0	Y15	Y9;	15	1	EKIGEGTyGVVYKAR	4.50	65	1621.78789	1	1.25

		m 2															
BCAR3	8412	breast cancer anti-estrogen resistance protein 3 isoform 1	387157906	DPHLLDPTVEyVK	Y11(Phospho)	T(8): 0.1; Y(11): 99.9	Y117	Y11;	4	1	LLDPTVEyVKFSKER	1.85	31	1605.75945	0	4.21
C11orf52	91894	uncharacterized protein C11orf52	131889517	HVHLENATEyATLR	Y10(Phospho)	T(8): 1.5; Y(10): 98.5; T(12): 0.0	Y103	Y10;	11	1	HLENATEyATLRFPQ	3.45	58	1733.79949	0	2.37
CDK1	983	cyclin-dependent kinase 1 isoform 2	16306492	IEKIGEGTyGVVYK	K3(Label:13C(6)); Y9(Phospho); K14(Label:13C(6))	T(8): 1.6; Y(9): 98.4; Y(13): 0.0	Y15	Y9;	8	1	EKIGEGTyGVVYKGR	5.70	64	1647.8416	1	2.45
CDK1	983	cyclin-dependent	16306492	IEKIGEGTyGVVYK	Y9(Phospho)	T(8): 1.6; Y(9): 98.4; Y(13): 0.0	Y15	Y9;	8	1	EKIGEGTyGVVYKGR	5.70	51	1635.80252	1	2.48

		kinase 1 isoform 2														
CDK1	983	cyclin-dependent kinase 1 isoform 2	16306492	IEKIGEGtyGVVYK	T8(Phospho); Y9(Phospho)	T(8): 100.0; Y(9): 100.0; Y(13): 0.0	T14; Y15	T8; Y9;	8	1	IEKIGEGtYGVVYKG;EKIGEGTyGVVYKGR	5.70	71	1715.76995	1	1.01
CDK1	983	cyclin-dependent kinase 1 isoform 2	16306492	IEKIGEGtyGVVYKGR	T8(Phospho); Y9(Phospho)	T(8): 100.0; Y(9): 100.0; Y(13): 0.0	T14; Y15	T8; Y9;	8	1	IEKIGEGtYGVVYKG;EKIGEGTyGVVYKGR	4.35	73	1928.89372	2	0.93
CDK1	983	cyclin-dependent kinase 1 isoform 2	16306492	IEKIGEGtyGVVYKGR	T8(Phospho); Y9(Phospho)	T(8): 100.0; Y(9): 100.0; Y(13): 0.0	T14; Y15	T8; Y9;	8	1	IEKIGEGtYGVVYKG;EKIGEGTyGVVYKGR	4.35	47	1928.89267	2	0.90
CDK2	1017	cyclin-dependent kinase	166362719	VEKIGEGtyGVVYK	T8(Phospho); Y9(Phospho)	T(8): 100.0; Y(9): 100.0; Y(13): 0.0	T14; Y15	T8; Y9;	15	1	VEKIGEGtYGVVYKA;EKIGEGTy	4.50	91	1701.75395	1	0.42

		2 isoform 2									GVVYKAR					
CDK1	983	cyclin-dependent kinase 1 isoform 2	16306492	IEKIGEGtyGVVYK	T8(Phospho); Y9(Phospho)	T(8): 100.0; Y(9): 99.9; Y(13): 0.1	T14; Y15	T8; Y9;	8	1	IEKIGEGtYGVVYKG;EKIGEGTyGVVYKGR	5.70	59	1715.76889	1	1.00
CDK2	1017	cyclin-dependent kinase 2 isoform 2	166362719	VEKIGEGTyGVVYK	Y9(Phospho)	T(8): 2.0; Y(9): 98.0; Y(13): 0.0	Y15	Y9;	15	1	EKIGEGTyGVVYKAR	4.50	55	1621.78562	1	1.27
SDCBP	6386	syntenin-1 isoform 1	56243522	VDKVIQAQTAFSANPANPAILSEASAPIPHDGNLyPR	Y35(Phospho)	T(9): 0.0; S(12): 0.0; S(22): 0.0; S(25): 0.0; Y(35): 100.0	Y46	Y35;	9	1	IPHDGNLyPRLYPEL	5.07	44	3952.95547	1	0.53
SDCBP	6386	syntenin-1 isoform 1	56243522	VDKVIQAQTAFSANPANPAILSEASAP	Y35(Phospho)	T(9): 0.0; S(12): 0.0; S(22): 0.0; S(25): 0.0; Y(35): 100.0	Y46	Y35;	9	1	IPHDGNLyPRLYPEL	5.07		3952.95957	1	0.53

				IPHDGNLyPR													
MAPK1	5594	mitogen-activated protein kinase 1	66932916	VADPDHDHTGFLTEyVATR	Y15(Phospho)	T(9): 0.0; T(13): 0.0; Y(15): 100.0; T(18): 0.0	Y187	Y15;	28	1	HTGFLTEyVATRWYR	5.53	47	2223.98746	0	1.80
MAPK1	5594	mitogen-activated protein kinase 1	66932916	VADPDHDHTGFLTEyVATR	Y15(Phospho)	T(9): 0.0; T(13): 0.0; Y(15): 100.0; T(18): 0.0	Y187	Y15;	28	1	HTGFLTEyVATRWYR	5.53	64	2223.97295	0	1.61
MAPK1	5594	mitogen-activated protein kinase 1	66932916	VADPDHDHTGFLTEyVATR	Y15(Phospho)	T(9): 0.0; T(13): 0.0; Y(15): 100.0; T(18): 0.0	Y187	Y15;	28	1	HTGFLTEyVATRWYR	5.53	49	2223.97116	0	1.54

MAPK3	5595	mitogen-activated protein kinase 3 isoform 3	158138507	IADPEHDHTGFLTEyVATR	Y15(Phospho)	T(9): 0.0; T(13): 0.0; Y(15): 99.9; T(18): 0.0	Y204	Y15;	14	1	HTGFLTEyVATRWYR	5.36	57	2252.00553	0	2.87
MAPK3	5595	mitogen-activated protein kinase 3 isoform 3	158138507	IADPEHDHTGFLTEyVATR	Y15(Phospho)	T(9): 0.0; T(13): 0.1; Y(15): 97.6; T(18): 2.4	Y204	Y15;	14	1	HTGFLTEyVATRWYR	5.36	65	2252.00762	0	3.19
MAPK1	5594	mitogen-activated protein kinase 1	66932916	VADPDHDHTGFLTEyVATR	Y15(Phospho)	T(9): 0.0; T(13): 0.1; Y(15): 99.9; T(18): 0.0	Y187	Y15;	28	1	HTGFLTEyVATRWYR	5.53	58	2223.99346	0	1.64
MAPK1	5594	mitogen-activated protei	66932916	VADPDHDHTGFLtEyVATR	T13(Phospho); Y15(Phospho)	T(9): 0.0; T(13): 100.0; Y(15): 100.0;	T185; Y187	T13; Y15;	28	1	HDHTGFLtEYVATRW;HTGFLTE	5.53	65	2303.93755	0	4.01

		n kinase 1				T(18): 0.0					yVATR WYR					
MAP K1	5594	mitogen-activated protein kinase 1	66932916	VADPDHDHTGFLtEyVATR	T13(Phospho); Y15(Phospho)	T(9): 0.0; T(13): 100.0; Y(15): 100.0; T(18): 0.0	T185; Y187	T13; Y15;	28	1	HDHTGFLtEYVATRW;HTGFLTEyVATRWYR	5.53	39	2303.93955	0	3.65
MAP K3	5595	mitogen-activated protein kinase 3 isoform 3	158138507	IADPEHDHTGFLtEyVATR	T13(Phospho); Y15(Phospho)	T(9): 0.0; T(13): 100.0; Y(15): 100.0; T(18): 0.0	T202; Y204	T13; Y15;	14	1	HDHTGFLtEYVATRW;HTGFLTEyVATRWYR	5.36	66	2331.9644	0	1.66
MAP K3	5595	mitogen-activated protein kinase 3 isoform 3	158138507	IADPEHDHTGFLtEyVATR	T13(Phospho); Y15(Phospho)	T(9): 0.0; T(13): 100.0; Y(15): 99.9; T(18): 0.1	T202; Y204	T13; Y15;	14	1	HDHTGFLtEYVATRW;HTGFLTEyVATRWYR	5.36	49	2331.96183	0	1.79

CTNND1	1500	catenin delta-1 isoform 2A	332688222	YRPSMEGyR	R2(Label:13C(6)); Y8(Phospho); R9(Label:13C(6))	Y(1): 0.0; S(4): 0.0; Y(8): 100.0	Y194	Y8;	3	1	YRPSMEGyRAPSRQD	2.47	30	1250.54119	0	1.45
CAV1	857	caveolin-1 isoform alpha	15451856	YVDSEGHLyTVPIR	T10(Phospho); R14(Label:13C(6))	Y(1): 0.0; S(4): 0.0; Y(9): 100.0; T(10): 0.0	Y14	Y9;	11	1	VDSEGHLyTVPIREQ	4.49	94	1734.81951	0	2.02
PTK2	5747	focal adhesion kinase 1 isoform a	24476013	YMEDSTyYK	M2(Oxidation); Y7(Phospho); K9(Label:13C(6))	Y(1): 0.0; S(5): 0.0; T(6): 0.0; Y(7): 98.2; Y(8): 1.7	Y576	Y7;	16	1	RYMEDSTyYKASKGK	2.39	57	1301.47331	0	2.34
PTK2	5747	focal adhesion kinase 1 isoform a	24476013	YMEDSTyyKASK	Y7(Phospho); Y8(Phospho)	Y(1): 0.0; S(5): 0.0; T(6): 0.1; Y(7): 99.9; Y(8): 99.9; S(11): 0.1	Y576; Y577	Y7; Y8;	7	1	RYMEDSTyYKASKGK;YMEDSTYyKASKGKL	2.85	49	1645.58823	1	3.11

PTK2	5747	focal adhesion kinase 1 isoform a	24476013	YMEDSTyYKASK	Y7(Phospho)	Y(1): 0.0; S(5): 0.0; T(6): 1.6; Y(7): 96.7; Y(8): 1.6; S(11): 0.0	Y576	Y7;	7	1	RYMEDSTyYKASKGK	2.85	48	1565.62223	1	2.73
PTK2	5747	focal adhesion kinase 1 isoform a	24476013	YMEDSTyYK	Y7(Phospho); K9(Label:13C(6))	Y(1): 0.0; S(5): 0.0; T(6): 1.8; Y(7): 96.4; Y(8): 1.8	Y576	Y7;	16	1	RYMEDSTyYKASKGK	2.39	48	1285.47783	0	2.32
PTK2	5747	focal adhesion kinase 1 isoform a	24476013	YMEDSTyYKASK	Y7(Phospho)	Y(1): 0.0; S(5): 0.0; T(6): 1.8; Y(7): 96.4; Y(8): 1.8; S(11): 0.0	Y576	Y7;	7	1	RYMEDSTyYKASKGK	2.85	59	1565.62273	1	2.37
PTK2	5747	focal adhesion kinase 1 isoform a	24476013	YMEDSTyyK	Y7(Phospho); Y8(Phospho); K9(Label:13C(6))	Y(1): 0.0; S(5): 0.0; T(6): 1.9; Y(7): 98.1; Y(8): 100.0	Y576; Y577	Y7; Y8;	16	1	RYMEDSTyYKASKGK;YMEDSTYyKASKGKL	2.39	45	1365.44377	0	3.12

PTK2	5747	focal adhesion kinase 1 isoform a	24476013	YMEDSTyyKASK	Y7(Phospho); Y8(Phospho)	Y(1): 0.0; S(5): 2.1; T(6): 2.1; Y(7): 96.0; Y(8): 99.9; S(11): 0.0	Y576; Y577	Y7; Y8;	7	1	RYMEDSTyYKASKGK;YMEDSTYyKASKGKL	2.85	52	1645.58769	1	3.24
STAT3	6774	signal transducer and activator of transcription 3 isoform 3	47458820	YCRPESQEHPEADPGSAAPyLK	C2(Carbamidomethyl); Y20(Phospho)	Y(1): 0.0; S(6): 0.0; S(16): 0.0; Y(20): 100.0	Y705	Y20;	4	1	DPGSAAPyLKTKFIC	4.49	38	2582.10221	0	0.76
STAT3	6774	signal transducer and activator of transcription 3 isoform 3	47458820	YCRPESQEHPEADPGSAAPyLK	C2(Carbamidomethyl); Y20(Phospho)	Y(1): 0.0; S(6): 0.0; S(16): 2.5; Y(20): 97.5	Y705	Y20;	4	1	DPGSAAPyLKTKFIC	4.49	40	2582.10259	0	0.76

STAT3	6774	signal transducer and activator of transcription 3 isoform 2	21618338	YCRPESQEHPEADPGAAPyLK	C2(Carbamidomethyl); Y19(Phospho)	Y(1): 0.0; S(6): 0.0; Y(19): 100.0	Y704	Y19;	4	1	ADPGAAPyLKTKFIC	3.90		2495.0689	0	0.92
STAT3	6774	signal transducer and activator of transcription 3 isoform 2	21618338	YCRPESQEHPEADPGAAPyLK	C2(Carbamidomethyl); Y19(Phospho)	Y(1): 0.0; S(6): 0.0; Y(19): 100.0	Y704	Y19;	4	1	ADPGAAPyLKTKFIC	3.90	31	2495.06852	0	0.85
EEF1A1	1915	elongation factor 1-alpha 1	4503471	YEEIVKEVSTyIK	K6(Label:13C(6)); Y11(Phospho); K13(Label:13C(6))	Y(1): 0.0; S(9): 0.0; T(10): 1.4; Y(11): 98.5	Y177	Y11;	5	1	IVKEVSTyIKKIGYN	3.09	65	1692.85283	1	0.69

SCEL	8796	sciellin isoform 3	238908501	YIQTVySTSDR	Y6(Phospho)	Y(1): 0.0; T(4): 0.0; Y(6): 98.2; S(7): 1.7; T(8): 0.0; S(9): 0.0	Y566	Y6;	8	1	GKYIQTVySTSDRSV	2.18	52	1412.61003	0	12.69
PTTG1IP	754	pituitary tumor-transforming gene 1 protein-interacting protein precursor	4757886	YGLFKEENPyAR	Y10(Phospho)	Y(1): 0.0; Y(10): 100.0	Y174	Y10;	8	1	LFKEENPyARFENN_	3.93	50	1566.70024	1	0.23
PTTG1IP	754	pituitary tumor-transforming gene 1 protein-	4757886	YGLFKEENPyAR	Y10(Phospho)	Y(1): 0.0; Y(10): 100.0	Y174	Y10;	8	1	LFKEENPyARFENN_	3.93	50	1566.69889	1	0.20

		interacting protein precursor														
LPP	4026	lipoma-preferred partner isoform a	5031887	YYEGYyAAGPGYGGR	Y6(Phospho)	Y(1): 0.0; Y(2): 0.0; Y(5): 2.3; Y(6): 97.7; Y(12): 0.0	Y301	Y6;	6	1	GRYYEGYyAAGPGYG	2.63	44	1723.67766	0	2.01
CFL1	1072	cofilin-1	5031635	YALYDATyETKESK	Y8(Phospho)	Y(1): 0.0; Y(4): 0.0; T(7): 0.0; Y(8): 98.0; T(10): 2.0; S(13): 0.0	Y89	Y8;	23	1	YALYDATyETKESKK	3.51	37	1761.76279	1	1.08
CFL1	1072	cofilin-1	5031635	YALYDATyETK	Y8(Phospho)	Y(1): 0.0; Y(4): 0.0; T(7): 1.8; Y(8): 98.2; T(10): 0.0	Y89	Y8;	6	1	YALYDATyETKESKK	3.02	50	1417.59233	0	0.95
CFL1	1072	cofilin-1	5031635	YALYDATyETKESK	Y8(Phospho)	Y(1): 0.0; Y(4): 0.0; T(7): 2.0; Y(8): 98.0; T(10): 0.0; S(13): 0.0	Y89	Y8;	23	1	YALYDATyETKESKK	3.51	70	1761.76128	1	0.82

PI4KA	5297	phosphatidylinositol 4-kinase alpha isoform 1	348041302	YAGEVyGMIR	Y6(Phospho); R10(Label:13C(6))	Y(1): 0.0; Y(6): 100.0	Y1154	Y6;	3	1	NRYAGEVyGMIRFSG	2.29	44	1244.54643	0	0.88
PTK2B	2185	protein-tyrosine kinase 2-beta isoform b	27886588	YIEDEDyyKASVTRLPIK	Y7(Phospho); Y8(Phospho)	Y(1): 0.0; Y(7): 100.0; Y(8): 100.0; S(11): 0.0; T(13): 0.0	Y579; Y580	Y7; Y8;	27	1	RYIEDEDyYKASVTR;YIEDEDYyKASVTRL	3.60	56	2363.0667	2	2.85
PTK2B	2185	protein-tyrosine kinase 2-beta isoform b	27886588	YIEDEDyyKASVTRLPIK	Y7(Phospho); Y8(Phospho)	Y(1): 0.0; Y(7): 100.0; Y(8): 100.0; S(11): 0.0; T(13): 0.0	Y579; Y580	Y7; Y8;	27	1	RYIEDEDyYKASVTR;YIEDEDYyKASVTRL	3.60	34	2363.0643	2	2.72
PTK2B	2185	protein-tyrosine kinase 2-beta isoform b	27886588	YIEDEDyyKASVTR	Y7(Phospho); Y8(Phospho)	Y(1): 0.0; Y(7): 100.0; Y(8): 100.0; S(11): 0.0; T(13): 0.0	Y579; Y580	Y7; Y8;	10	1	RYIEDEDyYKASVTR;YIEDEDYyKASVTRL	2.89	58	1911.74358	1	1.97

S100A11	6282	protein S100-A11	5032057	YAGKDGyNYTLSK	Y7(Phospho)	Y(1): 0.0; Y(7): 100.0; Y(9): 0.0; T(10): 0.0; S(12): 0.0	Y30	Y7;	4	1	KYAGKDGyNYTLSKT	3.14	38	1559.67851	1	0.75
MYO1B	4430	unconventional myosin-Ib isoform 2	44889481	yFLEMK	Y1(Phospho); M5(Oxidation)	Y(1): 100.0	Y823	Y1;	2	1	LQRIVQKyFLEMKNK	1.25	26	926.38127	0	3.02
VTA1	51534	vacuolar protein sorting-associated protein VTA1 homolog	21361741	yAGSALQYEDVSTAVQNLQK	Y1(Phospho)	Y(1): 100.0; S(4): 0.0; Y(8): 0.0; S(12): 0.0; T(13): 0.0	Y278	Y1;	13	1	RAQKYCKyAGSALQY	4.69	71	2265.0463	0	3.22
PPP1CA	5499	serine/threonine-protein phosphatase PP1-	45827798	yGQFSGLNPGGRPITPPR	Y1(Phospho)	Y(1): 100.0; S(5): 0.0; T(15): 0.0	Y262	Y1;	4	1	ADKNKGKyGQFSGLN	2.36	30	1993.97312	0	4.19

		alpha catalytic subunit isoform 2														
PTK2	5747	focal adhesion kinase 1 isoform a	24476013	yMEDSTYYK	Y1(Phospho)	Y(1): 100.0; S(5): 0.0; T(6): 0.0; Y(7): 0.0; Y(8): 0.0	Y570	Y1;	16	1	GDFGLSRyMEDSTYY	2.39	63	1279.4594	0	0.75
PTK2	5747	focal adhesion kinase 1 isoform a	24476013	yMEDSTyYK	Y1(Phospho); Y7(Phospho)	Y(1): 100.0; S(5): 0.1; T(6): 2.2; Y(7): 95.6; Y(8): 2.2	Y570; Y576	Y1; Y7;	16	1	GDFGLSRyMEDSTYY;RYMEDSTyYKASKGK	2.39	41	1359.42473	0	2.16
PDLIM5	10611	PDZ and LIM domain protein 5 isoform g	374093210	yTEFYHVPTHSDASK	Y1(Phospho)	Y(1): 100.0; T(2): 0.0; Y(5): 0.0; T(9): 0.0; S(11): 0.0; S(14): 0.0	Y144	Y1;	19	1	RKHIVERyTEFYHVP	4.45	30	1861.77811	0	1.84

ATIC	471	bifunctional purine biosynthesis protein PURH	20127454	yGMNPHQTPAQLYTLQPK	Y1(Phospho); K18(Label:13C(6))	Y(1): 100.0; T(8): 0.0; Y(13): 0.0; T(14): 0.0	Y208	Y1;	8	1	VSQMPLRyGMNPHQT	4.23	45	2173.02573	0	0.59
ATIC	471	bifunctional purine biosynthesis protein PURH	20127454	yGMNPHQTPAQLYTLQPK	Y1(Phospho); K18(Label:13C(6))	Y(1): 100.0; T(8): 0.0; Y(13): 0.0; T(14): 0.0	Y208	Y1;	8	1	VSQMPLRyGMNPHQT	4.23		2173.02593	0	0.54
PTTG1IP	754	pituitary tumor-transforming gene 1 protein-interacting protein precursor	4757886	yGLFKEENPYAR	Y1(Phospho)	Y(1): 100.0; Y(10): 0.0	Y165	Y1;	8	1	HDEIRKKyGLFKEEN	3.93	59	1566.70061	1	0.16

PTTG1IP	754	pituitary tumor-transforming gene 1 protein-interacting protein precursor	4757886	yGLFKEENPYAR	Y1(Phospho); K5(Label:13C(6)); R12(Label:13C(6))	Y(1): 100.0; Y(10): 0.0	Y165	Y1;	8	1	HDEIRKKyGLFKEEN	3.93	41	1578.7399	1	0.15
DSC3	1825	desmocollin-3 isoform Dsc3b preproprotein	148539848	YTySEWHSFTQPR	Y3(Phospho); R13(Label:13C(6))	Y(1): 2.0; T(2): 2.0; Y(3): 94.0; S(4): 2.0; S(8): 0.0; T(10): 0.0	Y818	Y3;	8	1	VDNCRYTySEWHSFT	2.20	58	1787.75078	0	0.23
IFNGR1	3459	interferon gamma receptor 1 precursor	4557880	yVSLITSYQPFSLEK	Y1(Phospho)	Y(1): 97.1; S(3): 2.9; T(6): 0.0; S(7): 0.0; Y(8): 0.0; S(12): 0.0	Y304	Y1;	5	1	ETKPESKyVSLITSY	2.18	35	1854.8958	0	1.38

MTHFS	10588	5-formyltetrahydrofolate cyclo-ligase isoform b	315113868	yRFQSNHMDMVR	Y1(Phospho)	Y(1): 97.3; S(5): 2.7	Y26	Y1;	6	1	KICFIPRyRFQSNHM	3.05		1663.68747	1	0.86
DTNB	1838	dystrobrevin beta isoform 8	372626425	yVFSQMSDSNGLMIFSK	S7(Phospho); M13(Oxidation)	Y(1): 99.8; S(4): 0.0; S(7): 0.2; S(9): 0.0; S(16): 0.0	Y93	Y1;	2	1	KMLDKLRyVFSQMSD	2.11		2049.90361	0	5.19
VTA1	51534	vacuolar protein sorting-associated protein VTA1 homolog	21361741	yAGSALQYEDVSTAVQNLQK	Y1(Phospho)	Y(1): 99.9; S(4): 0.1; Y(8): 0.0; S(12): 0.0; T(13): 0.0	Y278	Y1;	13	1	RAQKYCKyAGSALQY	4.69	77	2265.04814	0	2.93
EPHA4	2043	ephrin type-A receptor 4 precur	4758280	VLEDDPEAAYtTR	Y10(Phospho)	Y(10): 1.8; T(11): 96.4; T(12): 1.8	T780	T11;	5	1	DDPEAAYtTRGGKIP	3.31	45	1559.6635	0	17.70

		sor														
NED D9	4739	enhancer of filamentation 1 isoform 3	215422375	DEAGLREKDyDFPPPMR	R6(Label:13C(6)); K8(Label:13C(6)); Y10(Phospho); R17(Label:13C(6))	Y(10): 100.0	Y241	Y10;	30	1	AGLREKDyDFPPPMR	4.29	42	2133.98142	2	14.12
NED D9	4739	enhancer of filamentation 1 isoform 3	215422375	DEAGLREKDyDFPPPMR	Y10(Phospho)	Y(10): 100.0	Y241	Y10;	30	1	AGLREKDyDFPPPMR	4.29	49	2115.91948	2	22.74
NED D9	4739	enhancer of filamentation 1 isoform 3	215422375	DEAGLREKDyDFPPPMR	Y10(Phospho)	Y(10): 100.0	Y241	Y10;	30	1	AGLREKDyDFPPPMR	4.29	31	2115.93271	2	13.28
NED D9	4739	enhancer of filamentation 1	215422375	ANPQERDGVyDVPLHNPPDAK	Y10(Phospho)	Y(10): 100.0	Y345	Y10;	2	1	PQERDGVyDVPLHNP	4.45		2412.0972	1	10.79

		isoform 3														
TNS3	64759	tensin-3	65288071	QQQMVVAHQySFAPDGEAR	Y10(Phospho)	Y(10): 100.0; S(11): 0.0	Y601	Y10;	4	1	QMVVAHQySFAPDGE	3.79	33	2241.97702	0	2.15
TYK2	7297	non-receptor tyrosine-protein kinase TYK2	187608615	LLAQAEGEPCyIR	C10(Carbamidomethyl); Y11(Phospho); R13(Label:13C(6))	Y(11): 100.0	Y292	Y11;	4	1	QAEGEPCyIRDSGVA	3.65	56	1605.74419	0	1.32
TJP2	9414	tight junction protein ZO-2 isoform 4	282165804	IEIAQKHPDIyAVPIK	K6(Label:13C(6)); Y11(Phospho); K16(Label:13C(6))	Y(11): 100.0	Y1085	Y11;	4	1	AQKHPDIyAVPIKTH	4.24	43	1927.06279	1	1.38
BCAR1	9564	breast cancer anti-estrogen resistance protei	282398123	HLLAPGPQDIyDVPPVR	Y11(Phospho)	Y(11): 100.0	Y247	Y11;	12	1	APGPQDIyDVPPVRG	3.89	83	1966.97685	0	1.97

		n 1 isoform 7														
TJP2	9414	tight junction protein ZO-2 isoform 4	282165804	IEIAQKHPDIyAVPIK	Y11(Phospho)	Y(11): 100.0	Y1085	Y11;	4	1	AQKHPDIyAVPIKTH	4.24		1915.02005	1	1.32
TKT	7086	transketolase isoform 1	4507521	NMAEQIIQEIySQIQSK	Y11(Phospho)	Y(11): 98.3; S(12): 1.7; S(16): 0.0	Y275	Y11;	10	1	EQIIQEIySQIQSKK	2.98	48	2102.98239	0	2.10
CTNNA1	1495	catenin alpha-1	55770844	NAGNEQDLGIQyK	Y12(Phospho); K13(Label:13C(6))	Y(12): 100.0	Y177	Y12;	2	1	EQDLGIQyKALKPEV	3.49	41	1535.68535	0	0.82
BCAR1	9564	breast cancer anti-estrogen resistance protein 1 isoform 7	282398123	GPNGRDPLLEVyDVPPSVEK	Y12(Phospho)	Y(12): 100.0; S(17): 0.0	Y285	Y12;	8	1	RDPLLEVyDVPPSVE	3.33		2261.08574	1	3.83

BCAR1	9564	breast cancer anti-estrogen resistance protein 1 isoform 7	282398123	GPNGRDPLLEVyDVPPSVEK	Y12(Phospho)	Y(12): 100.0; S(17): 0.0	Y285	Y12;	8	1	RDPLLEVyDVPPSVE	3.33		2261.08646	1	0.91
VCL	7414	vinculin isoform meta-VCL	7669550	ILLRNPGNQAAyEHFETMK	R4(Label:13C(6)); Y12(Phospho); K19(Label:13C(6))	Y(12): 100.0; T(17): 0.0	Y692	Y12;	4	1	NPGNQAAyEHFETMK	4.50	41	2324.13169	1	0.69
ARHGAP42	143872	rho GTPase-activating protein 42	221307575	KLWLEAMDGKEPIyTLPAIISK	Y14(Phospho)	Y(14): 100.0; T(15): 0.0; S(21): 0.0	Y376	Y14;	5	1	MDGKEPIyTLPAIIS	4.06	36	2596.35129	2	10.81

TMEM192	201931	transmembrane protein 192	154240704	AKPEPDILEEEKIyAYPSNITSETGFR	Y14(Phospho)	Y(14): 100.0; Y(16): 0.0; S(18): 0.0; T(21): 0.0; S(22): 0.0; T(24): 0.0	Y213	Y14;	3	1	ILEEEKIyAYPSNIT	3.19		3177.50901	1	0.53
PEAK1	79834	pseudopodium-enriched atypical kinase 1	148368962	NAIKVPIVINPNAyDNLAIYK	Y14(Phospho)	Y(14): 100.0; Y(20): 0.0	Y635	Y14;	8	1	IVINPNAyDNLAIYK	4.40	31	2423.27764	1	5.67
PEAK1	79834	pseudopodium-enriched atypical kinase 1	148368962	NAIKVPIVINPNAyDNLAIYK	Y14(Phospho)	Y(14): 100.0; Y(20): 0.0	Y635	Y14;	8	1	IVINPNAyDNLAIYK	4.40	55	2423.26847	1	4.60
BCR	613	breakpoint cluster region protein isofor	82546845	KGHGQPGADAEKPFyVNVEFHHER	Y15(Phospho)	Y(15): 100.0	Y177	Y15;	2	1	ADAEKPFyVNVEFHH	3.20		2829.28774	1	0.30

		m 2														
PTK2	5747	focal adhesion kinase 1 isoform a	24476013	CIGEGQFGDVHQGIyMSPENPALAVAIK	C1(Carbamidomethyl); Y15(Phospho)	Y(15): 99.9; S(17): 0.1	Y441	Y15;	4	1	GDVHQGIyMSPENPA	3.22	50	3081.42624	0	3.14
LYN	4067	tyrosine-protein kinase Lyn isoform A	4505055	LYAVVTREEPIyIITEYMAK	R7(Label:13C(6)); Y12(Phospho); K20(Label:13C(6))	Y(2): 0.0; T(6): 0.1; Y(12): 99.9; T(15): 0.1; Y(17): 0.0	Y316	Y12;	9	1	VTREEPIyIITEYMA	2.87		2494.27457	1	0.44
PTTG1IP	754	pituitary tumor-transforming gene 1 protein-interacting protein	4757886	KYGLFKEENPyAR	Y11(Phospho)	Y(2): 0.0; Y(11): 100.0	Y174	Y11;	4	1	LFKEENPyARFENN_	3.66	39	1694.79399	2	0.23

		precursor															
PTTG1IP	754	pituitary tumor-transforming gene 1 protein-interacting protein precursor	4757886	KYGLFKEENPyAR	Y11(Phospho)	Y(2): 0.0; Y(11): 100.0	Y174	Y11;	4	1	LFKEENPyARFENN_	3.66	37	1694.79563	2	0.20
DYRK1A	1859	dual specificity tyrosine-phosphorylation-regulated kinase 1A isoform 5	18765756	IYQyIQSR	Y4(Phospho)	Y(2): 0.0; Y(4): 100.0; S(7): 0.0	Y321	Y4;	9	1	LGQRIYQyIQSRFYR	2.28	52	1150.53105	0	1.02

ANXA5	308	annexin A5	4502107	LYDAyELK	Y5(Phospho)	Y(2): 0.0; Y(5): 100.0	Y94	Y5;	2	1	PSRLYDAyELKHALK	1.75	37	1094.48003	0	0.43
ANXA5	308	annexin A5	4502107	LYDAyELKHALK	Y5(Phospho); K8(Label:13C(6)); K12(Label:13C(6))	Y(2): 0.0; Y(5): 100.0	Y94	Y5;	3	1	PSRLYDAyELKHALK	2.25	48	1555.80411	1	0.43
CTNND1	1500	catenin delta-1 isoform 2A	332688222	HYEDGYPGGSDNyGSLSR	Y13(Phospho); R18(Label:13C(6))	Y(2): 0.0; Y(6): 0.0; S(10): 0.0; Y(13): 100.0; S(15): 0.0; S(17): 0.0	Y174	Y13;	13	1	YPGGSDNyGSLSRVT	5.11	74	2059.81327	0	1.05
CTNND1	1500	catenin delta-1 isoform 2A	332688222	HYEDGYPGGSDNyGSLSR	Y13(Phospho)	Y(2): 0.0; Y(6): 0.0; S(10): 0.0; Y(13): 99.9; S(15): 0.0; S(17): 0.0	Y174	Y13;	13	1	YPGGSDNyGSLSRVT	5.11	71	2053.79375	0	1.07
PIK3R1	5295	phosphatidylinositol 3-kinase regula	32455248	EYDRLyEEYTR	Y6(Phospho)	Y(2): 0.0; Y(6): 100.0; Y(9): 0.0; T(10): 0.0	Y467	Y6;	3	1	SREYDRLyEEYTRTS	2.72		1616.66423	1	2.60

		tory subunit alpha isoform 1														
NEDD9	4739	enhancer of filamentation 1 isoform 3	215422375	LyQVPNPQAAPR	Y2(Phospho)	Y(2): 100.0	Y92	Y2;	4	1	TFGQQKLyQVPNPQA	2.92	51	1433.6939	0	39.41
PTPN11	5781	tyrosine-protein phosphatase non-receptor type 11 isoform 1	33356177	VyENVGLMQQQK	Y2(Phospho); K12(Label:13C(6))	Y(2): 100.0	Y580	Y2;	4	1	REDSARVyENVGLMQ	3.64	75	1522.70403	0	1.45
PTPN11	5781	tyrosine-protein phosphatase non-recept	33356177	VyENVGLMQQQK	Y2(Phospho); M8(Oxidation)	Y(2): 100.0	Y580	Y2;	4	1	REDSARVyENVGLMQ	3.64	37	1532.68205	0	1.43

		or type 11 isoform 1														
ITGB4	3691	integrin beta-4 isoform 3 precursor	54607033	DySTLTSVSSHDSR	Y2(Phospho)	Y(2): 100.0; S(3): 0.0; T(4): 0.0; T(6): 0.0; S(7): 0.0; S(9): 0.0; S(10): 0.0; S(13): 0.0	Y1440	Y2;	8	1	STTLPRDySTLTSVS	3.80	72	1634.67034	0	2.82
EPS8	2059	epidermal growth factor receptor kinase substrate 8	56682953	VySQITVQK	Y2(Phospho); K9(Label:13C(6))	Y(2): 100.0; S(3): 0.0; T(6): 0.0	Y774	Y2;	4	1	CPEGARVySQITVQK	2.98	51	1151.58281	0	2.03
TARS	6897	threonine--tRNA ligase, cytoplasmic isoform 1	38202255	IyGISFPDPK	Y2(Phospho)	Y(2): 100.0; S(5): 0.0	Y298	Y2;	3	1	METLQRIyGISFPDP	2.13	48	1216.56474	0	1.49

SDCBP	6386	syntenin-1 isoform 1	56243522	LyPELSQYMGLSLNEEEIR	Y2(Phospho); R19(Label:13C(6))	Y(2): 100.0; S(6): 0.0; Y(8): 0.0; S(12): 0.0	Y50	Y2;	10	1	GNLYPRLyPELSQYM	2.65	68	2370.10723	0	0.63
ANXA2	302	annexin A2 isoform 2	209862831	AyTNFDAER	Y2(Phospho)	Y(2): 100.0; T(3): 0.0	Y30	Y2;	4	1	AYGSVKAyTNFDAER	2.22	52	1166.45073	0	0.59
MLLT4	4301	afadin isoform 2	90819233	EyFTFPASK	Y2(Phospho); K9(Label:13C(6))	Y(2): 100.0; T(4): 0.0; S(8): 0.0	Y1230	Y2;	4	1	TQTYTREyFTFPASK	1.67	36	1175.50774	0	0.47
PGAM1	5223	phosphoglycerate mutase 1	4505753	HyGGLTGLNKAETAAK	Y2(Phospho)	Y(2): 100.0; T(6): 0.0; T(13): 0.0	Y92	Y2;	4	1	WRLNERHyGGLTGLN	5.79	98	1710.8206	1	0.59
PGAM1	5223	phosphoglycerate mutase 1	4505753	HyGGLTGLNKAETAAK	Y2(Phospho)	Y(2): 100.0; T(6): 0.0; T(13): 0.0	Y92	Y2;	4	1	WRLNERHyGGLTGLN	5.79	53	1710.82083	1	0.53
ARHGAP5	394	rho GTPase-activating protein 5 isoform b	71834861	GYSDEIyVVPDDSQNR	Y7(Phospho); R16(Label:13C(6))	Y(2): 4.1; S(3): 4.1; Y(7): 91.8; S(13): 0.0	Y1109	Y7;	6	1	KGYSDEIyVVPDDSQ	2.44	35	1942.82463	0	0.14

ANXA2	302	annexin A2 isoform 2	209862831	AyTNFDAERDALNIETAIK	Y2(Phospho)	Y(2): 98.4; T(3): 1.6; T(16): 0.0	Y30	Y2;	8	1	AYGSVKAyTNFDAER	4.29	72	2235.03374	1	0.52
ANXA2	302	annexin A2 isoform 2	209862831	AyTNFDAERDALNIETAIK	Y2(Phospho)	Y(2): 99.9; T(3): 0.1; T(16): 0.0	Y30	Y2;	8	1	AYGSVKAyTNFDAER	4.29	41	2235.02964	1	0.56
PIK3R2	5296	phosphatidylinositol 3-kinase regulatory subunit beta	4826908	EyDQLYEEYTR	Y6(Phospho)	Y(2): 99.9; Y(6): 0.1; Y(9): 0.1; T(10): 0.0	Y460	Y2;	4	1	YQDKSREyDQLYEEY	2.48	38	1588.62065	0	2.90
C9orf169	375791	UPF0574 protein C9orf169	259013211	GNQGAAPIQNQQAWQQPGNPySSSQR	Y21(Phospho)	Y(21): 88.2; S(22): 3.9; S(23): 3.9; S(24): 3.9	Y91	Y21;	8	1	WQQPGNPySSSQRQA	3.01		2892.28177	0	1.38
GSTP1	2950	glutathione S-transferase P	4504183	PPYTVVyFPVR	Y7(Phospho)	Y(3): 0.0; T(4): 0.0; Y(7): 100.0	Y8	Y7;	1	1	MPPYTVVyFPVRGRC	0.00	57	1417.69255	0	2.06
INSR	3643	insulin receptor	119395738	DIYETDyYR	Y7(Phospho)	Y(3): 0.0; T(5): 0.0; Y(7): 98.3; Y(8): 1.7	Y1177	Y7;	5	1	RDIYETDyYRKGGKG	2.27	41	1317.50395	0	0.67

		isoform Short preproprotein															
INSR	3643	insulin receptor isoform Short preproprotein	119395738	DIYETDyYRK	Y7(Phospho)	Y(3): 0.0; T(5): 1.8; Y(7): 96.4; Y(8): 1.8	Y1177	Y7;	6	1	RDIYETDyYRKGGKG	2.53	38	1445.59734	1	0.63
MET	4233	hepatocyte growth factor receptor isoform b precursor	42741655	DMYDKEyYSVHNK	K5(Label:13C(6)); Y7(Phospho); K13(Label:13C(6))	Y(3): 0.0; Y(7): 100.0; Y(8): 0.0; S(9): 0.0	Y1234	Y7;	17	1	RDMYDKEyYSVHNKT	3.93	80	1783.7459	1	0.56

MET	4233	hepatocyte growth factor receptor isoform b precursor	42741655	DMYDKEyYSVHNK	M2(Oxidation); K5(Label:13C(6)); Y7(Phospho); K13(Label:13C(6))	Y(3): 0.0; Y(7): 100.0; Y(8): 0.0; S(9): 0.0	Y1234	Y7;	17	1	RDMYDKEyYSVHNKT	3.93	40	1799.73941	1	0.82
MET	4233	hepatocyte growth factor receptor isoform b precursor	42741655	DMYDKEyYSVHNK	Y7(Phospho)	Y(3): 0.0; Y(7): 100.0; Y(8): 0.0; S(9): 0.0	Y1234	Y7;	17	1	RDMYDKEyYSVHNKT	3.93	37	1771.70542	1	0.61
WASL	8976	neural Wiskott-Aldrich syndrome protein	51702526	VIyDFIEK	Y3(Phospho)	Y(3): 100.0	Y256	Y3;	4	1	RETSKVIyDFIEKTG	2.35	41	1106.51628	0	2.49
ITSN2	50618	intersectin-2	194294525	LIyLVPEK	Y3(Phospho)	Y(3): 100.0	Y553	Y3;	2	1	EYQNKLIyL	1.89	37	1054.56084	0	1.16

		isoform 2									VPEKQL					
CTNNA1	1495	catenin alpha-1	55770844	LVyDGIR	Y3(Phospho)	Y(3): 100.0	Y619	Y3;	2	1	IDASRLVyDGIRDIR	1.74	39	915.43334	0	0.79
JUP	3728	junction plakoglobin	12056468	LNyGIPAIVK	Y3(Phospho); K10(Label:13C(6))	Y(3): 100.0	Y480	Y3;	2	1	QNSVRLNyGIPAIVK	2.46	35	1173.6375	0	0.53
DDX3X	1654	ATP-dependent RNA helicase DDX3X isoform 3	301171475	KQyPISLVLAPTR	Y3(Phospho)	Y(3): 100.0; S(6): 0.0; T(12): 0.0	Y250	Y3;	4	1	RYGRRKQyPISLVLA	3.62	77	1565.84453	1	1.78
C9orf169	375791	UPF0574 protein C9orf169	259013211	NPyAHISIPR	Y3(Phospho)	Y(3): 100.0; S(7): 0.0	Y12	Y3;	5	1	EMVVKNPyAHISIPR	2.67	53	1247.59197	0	2.69

SHB	6461	SH2 domain-containing adapter protein B	106879210	LDyCGGSGEPGGVQR	Y3(Phospho); C4(Carbamidomethyl); R15(Label:13C(6))	Y(3): 100.0; S(7): 0.0	Y114	Y3;	4	1	RAMCRLDyCGGSGEP	3.11	95	1637.6751	0	0.86
PKP3	11187	plakophilin-3	6005830	GQyHTLQAGFSSR	Y3(Phospho); R13(Label:13C(6))	Y(3): 100.0; T(5): 0.0; S(11): 0.0; S(12): 0.0	Y84	Y3;	4	1	RGTSRGQyHTLQAGF	4.15	52	1537.68938	0	0.47
PKP3	11187	plakophilin-3	6005830	ADyDTLSLR	Y3(Phospho)	Y(3): 100.0; T(5): 0.0; S(7): 0.0	Y176	Y3;	4	1	GVGSRADyDTLSLRS	2.56	56	1133.48699	0	1.02
INSR	3643	insulin receptor isoform Short preproprotein	119395738	DIyETDyYRK	Y3(Phospho); Y7(Phospho); R9(Label:13C(6)); K10(Label:13C(6))	Y(3): 100.0; T(5): 2.2; Y(7): 95.5; Y(8): 2.2	Y1173; Y1177	Y3; Y7;	6	1	FGMTRDIyETDYYRK;RDIYETDyYRKGGKG	2.53	28	1537.60564	1	0.44
PIK3R1	5295	phosphatidylinositol 3-kinase	32455248	DQyLMWLTQK	Y3(Phospho)	Y(3): 100.0; T(8): 0.0	Y580	Y3;	2	1	LRKTRDQyLMWLTQK	1.60	35	1405.62175	0	3.20

		regulatory subunit alpha isoform 1															
KIAA1217	56243	sickle tail protein homolog isoform 1	50843820	NVyYELNDVR	Y3(Phospho); R10(Label:13C(6))	Y(3): 100.0; Y(4): 0.0	Y244	Y3;	4	1	KDESRNVyYELNDVR	2.44	39	1370.60918	0	1.35	
RPL15	6138	60S ribosomal protein L15 isoform 1	15431293	QGyVIYR	Y3(Phospho)	Y(3): 100.0; Y(6): 0.0	Y59	Y3;	2	1	GYKAKQGyVIYRIRV	1.75	39	978.44414	0	2.02	
BAIAP2	10458	brain-specific angiogenesis inhibitor 1-associated protein 2	5453565	QRPYSVAVPAFSQGLDDyGAR	Y18(Phospho)	Y(4): 0.0; S(5): 0.0; S(12): 0.0; Y(18): 100.0	Y505	Y18;	3	1	FSQGLDDyGARSMSS	4.01	37	2377.09275	0	2.05	

		isoform 3														
ITGB4	3691	integrin beta-4 isoform 3 precursor	54607033	VCAYGAQGEGPySSLVSCR	C2(Carbamidomethyl); Y12(Phospho); C18(Carbamidomethyl)	Y(4): 0.0; Y(12): 98.2; S(13): 1.8; S(14): 0.0; S(17): 0.0	Y1207	Y12;	7	1	GAQGEGPySSLVSCR	4.06	90	2140.88359	0	2.48
ITGB4	3691	integrin beta-4 isoform 3 precursor	54607033	VCAYGAQGEGPySSLVSCR	C2(Carbamidomethyl); Y12(Phospho); C18(Carbamidomethyl)	Y(4): 0.0; Y(12): 98.4; S(13): 1.5; S(14): 0.0; S(17): 0.0	Y1207	Y12;	7	1	GAQGEGPySSLVSCR	4.06	71	2140.88156	0	2.75
NEDD9	4739	enhancer of filamentation 1 isoform 3	215422375	DGVyDVPLHNPPDAK	Y4(Phospho)	Y(4): 100.0	Y345	Y4;	4	1	PQERDGVyDVPLHNP	3.58	35	1716.76297	0	13.95

NED D9	4739	enhan cer of filame ntatio n 1 isofor m 3	21542237 5	DGVy DVPL HNPP DAK	Y4(Pho spho)	Y(4): 100.0	Y345	Y4;	4	1	PQER DGVy DVPL HNP	3.58	70	1716. 76311	0	13.86
NED D9	4739	enhan cer of filame ntatio n 1 isofor m 3	21542237 5	EKDy DFPPP MR	Y4(Pho spho)	Y(4): 100.0	Y241	Y4;	32	1	AGLR EKDy DFPPP MR	3.14	55	1474. 60698	1	7.90
PAR D3	56288	partiti oning defect ive 3 homol og isofor m 7	29627819 8	ERDyA EIQDF HR	Y4(Pho spho)	Y(4): 100.0	Y990	Y4;	4	1	RQAR ERDyA EIQDF H	3.81	66	1658. 69512	1	0.71
PAR D3	56288	partiti oning defect ive 3 homol og isofor m 7	29627819 8	ERDyA EIQDF HR	Y4(Pho spho)	Y(4): 100.0	Y990	Y4;	4	1	RQAR ERDyA EIQDF H	3.81		1658. 69455	1	0.58

RPS10	6204	40S ribosomal protein S10	4506679	IAIyELLFK	Y4(Phospho); K9(Label:13C(6))	Y(4): 100.0	Y12	Y4;	2	1	KKNRIAIyELLFKEG	2.38	63	1195.64665	0	0.97
PTRF	284119	polymerase I and transcript release factor	42734430	VMIyQDEVKLPAK	Y4(Phospho); K9(Label:13C(6)); K13(Label:13C(6))	Y(4): 100.0	Y156	Y4;	2	1	RNFKVMIyQDEVKLP	1.84	51	1625.84343	1	0.87
DDX3X	1654	ATP-dependent RNA helicase DDX3X isoform 3	301171475	RKQyPISLVLAPTR	Y4(Phospho)	Y(4): 100.0; S(7): 0.0; T(13): 0.0	Y250	Y4;	3	1	RYGRRKQyPISLVLA	3.82	32	1721.94541	2	1.91
INPPL1	3636	phosphatidylinositol 3,4,5-trisphosphate 5-phosphatase	222136583	ERLyEWISIDKDEAGAK	Y4(Phospho)	Y(4): 100.0; S(8): 0.0	Y886	Y4;	8	1	LGTRERLyEWISIDK	3.95	55	2102.978	2	1.19

		2														
TBCB	1155	tubulin-folding cofactor B	50428925	LGEyEDVSR	Y4(Phospho); R9(Label:13C(6))	Y(4): 100.0; S(8): 0.0	Y98	Y4;	4	1	SGARLGEyEDVSRVE	2.08	69	1153.48747	0	0.95
GPRC5C	55890	G-protein coupled receptor family C group 5 member C isoform b precursor	40217833	GVGyETILKEQK	Y4(Phospho)	Y(4): 100.0; T(6): 0.0	Y336	Y4;	4	1	YPTRGVGyETILKEQ	2.86	50	1444.70793	1	0.23
GPRC5C	55890	G-protein coupled receptor	40217833	GVGyETILK	Y4(Phospho); K9(Label:13C(6))	Y(4): 100.0; T(6): 0.0	Y336	Y4;	4	1	YPTRGVGyETILKEQ	1.53	42	1065.53313	0	0.31

		family C group 5 member C isoform b precursor														
VASP	7408	vasodilator-stimulated phosphoprotein	4507869	VQIyHNPTANSFR	Y4(Phospho)	Y(4): 100.0; T(8): 0.0; S(11): 0.0	Y39	Y4;	5	1	AFSRVQIyHNPTANS	3.54	59	1626.7404	0	1.05
FRK	2444	tyrosine-protein kinase FRK	4503787	HGHyFVALFDYQAR	Y4(Phospho)	Y(4): 100.0; Y(11): 0.0	Y46	Y4;	14	1	QSQRHGHyFVALFDY	3.92	44	1803.79685	0	2.95
FRK	2444	tyrosine-protein kinase FRK	4503787	HGHyFVALFDYQAR	Y4(Phospho)	Y(4): 100.0; Y(11): 0.0	Y46	Y4;	14	1	QSQRHGHyFVALFDY	3.92	42	1803.7974	0	2.17
FRK	2444	tyrosine-protein	4503787	HGHyFVALFDYQAR	Y4(Phospho)	Y(4): 100.0; Y(11): 0.0	Y46	Y4;	14	1	QSQRHGHyFVALFDY	3.92	55	1803.79717	0	1.62

		kinase FRK														
FRK	2444	tyrosine-protein kinase FRK	4503787	HGHyFVALFDYQAR	Y4(Phospho); R14(Label:13C(6))	Y(4): 100.0; Y(11): 0.0	Y46	Y4;	14	1	QSQRHGHyFVALFDY	3.92	68	1809.81865	0	1.52
HEG1	57493	protein HEG homolog 1 precursor	153792110	NGLyPAYTGLPGSR	Y4(Phospho); R14(Label:13C(6))	Y(4): 100.0; Y(7): 0.0; T(8): 0.0; S(13): 0.0	Y1347	Y4;	6	1	ELERNGLyPAYTGLP	2.67	53	1551.72954	0	0.40
EPHB3	2049	ephrin type-B receptor 3 precursor	17975768	LQQYIAPGMKVyIDPFTYEDPNEAVR	Y12(Phospho)	Y(4): 2.9; Y(12): 97.1; T(17): 0.0; Y(18): 0.0	Y608	Y12;	6	1	IAPGMKVyIDPFTYE	2.63		3137.47556	1	2.20
INPPL1	3636	phosphatidylinositol 3,4,5-trisphosphate 5-phosphatase 2	222136583	ERLyEWISIDKDEAGAK	Y4(Phospho)	Y(4): 97.8; S(8): 2.2	Y886	Y4;	8	1	LGTRERLyEWISIDK	3.95	38	2102.97978	2	1.46

TLN1	7094	talin-1	223029410	ALDyYMLR	Y4(Phospho)	Y(4): 98.6; Y(5): 1.4	Y70	Y4;	4	1	EAGKALDyYMLRNGD	1.75	40	1124.48479	0	1.41
PTPRA	5786	receptor-type tyrosine-protein phosphatase alpha isoform 2 precursor	18450369	VVQEYIDAFSDyANFK	Y12(Phospho); K16(Label:13C(6))	Y(5): 0.0; S(10): 0.0; Y(12): 100.0	Y789	Y12;	13	1	YIDAFSDyANFK_	4.22	69	1994.88945	0	0.43
PTPRA	5786	receptor-type tyrosine-protein phosphatase alpha isoform 2 precursor	18450369	VVQEYIDAFSDyANFK	Y12(Phospho); K16(Label:13C(6))	Y(5): 0.0; S(10): 0.0; Y(12): 100.0	Y789	Y12;	13	1	YIDAFSDyANFK_	4.22	60	1994.88956	0	0.42

BCAR1	9564	breast cancer anti-estrogen resistance protein 1 isoform 7	282398123	VGQGYVYEAAQPEQDEyDIPR	Y17(Phospho)	Y(5): 0.0; Y(7): 0.0; Y(17): 100.0	Y232	Y17;	14	1	AQPEQDEyDIPRHLL	3.68	53	2507.07622	0	1.76
CTNNA1	1495	catenin alpha-1	55770844	EVKEyAQVFR	K3(Label:13C(6)); Y5(Phospho); R10(Label:13C(6))	Y(5): 100.0	Y419	Y5;	4	1	NEKEVKEyAQVFREH	2.92	47	1360.67058	1	0.37
PRKCD	5580	protein kinase C delta type	31377782	GRGEyFAIK	R2(Label:13C(6)); Y5(Phospho); K9(Label:13C(6))	Y(5): 100.0	Y374	Y5;	4	1	ELKGRGEyFAIKALK	2.76	52	1132.55901	1	0.49
TNS3	64759	tensin-3	65288071	AGVDyAPNLPPFPSPADVK	Y5(Phospho)	Y(5): 100.0; S(14): 0.0	Y802	Y5;	4	1	WGKAGVDyAPNLPPF	2.48	38	2034.96037	0	1.37

JAK2	3717	tyrosine-protein kinase JAK2	4826776	EVGDyGQLHETEVLLK	Y5(Phospho)	Y(5): 100.0; T(11): 0.0	Y570	Y5;	7	1	VRREVGDyGQLHETE	3.15	30	1909.89713	0	1.19
JAK2	3717	tyrosine-protein kinase JAK2	4826776	EVGDyGQLHETEVLLK	Y5(Phospho); K16(Label:13C(6))	Y(5): 100.0; T(11): 0.0	Y570	Y5;	7	1	VRREVGDyGQLHETE	3.15	66	1915.9157	0	1.37
PLCG1	5335	1-phosphatidylinositol 4,5-bisphosphate phosphodiesterase gamma-1 isoform b	33598946	NPGFyVEANPMPTFK	Y5(Phospho)	Y(5): 100.0; T(13): 0.0	Y783	Y5;	5	1	EGRNPGFyVEANPMP	2.40	43	1791.78032	0	2.60

ACTB	60	actin, cytoplasmic 1	4501885	EKLCyVALDFEQEMATAASSSSLEK	K2(Label:13C(6)); C4(Carbamidomethyl); Y5(Phospho); K25(Label:13C(6))	Y(5): 100.0; T(16): 0.0; S(19): 0.0; S(20): 0.0; S(21): 0.0; S(22): 0.0	Y218	Y5;	2	1	DIKEKLCyVALDFEQ	3.07	37	2899.32236	1	0.85
AHNAK	79026	neuroblast differentiation-associated protein AHNAK isoform 1	61743954	VKGEyDVTVPK	K2(Label:13C(6)); Y5(Phospho); K11(Label:13C(6))	Y(5): 100.0; T(8): 0.0	Y715	Y5;	4	1	GTKVKGEyDVTVPKL	3.02		1326.67412	1	0.54
KIAA1217	56243	sickle tail protein homolog isoform 1	50843820	NEGFyADPYLYHEGR	Y5(Phospho); R15(Label:13C(6))	Y(5): 100.0; Y(9): 0.0; Y(11): 0.0	Y393	Y5;	8	1	MYRNEGFyADPYLYH	3.32	75	1916.79399	0	0.92

BCAR1	9564	breast cancer anti-estrogen resistance protein 1 isoform 7	282398123	VGQGYVyEAAQPEQDEYDIPR	Y7(Phospho)	Y(5): 2.3; Y(7): 97.7; Y(17): 0.0	Y222	Y7;	14	1	RVGQGYVyEAAQPEQ	3.68	37	2507.07451	0	2.79
BCAR1	9564	breast cancer anti-estrogen resistance protein 1 isoform 7	282398123	VGQGYVYEAAQPEQDEyDIPR	Y7(Phospho); Y17(Phospho)	Y(5): 50.0; Y(7): 50.0; Y(17): 100.0	Y232	Y17;	14	1	AQPEQDEyDIPRHLL	3.68	33	2587.04166	0	2.44
AHNAK	79026	neuroblast differentiation-associated protein AHNAK	61743954	MKGDyDVTVPK	K2(Label:13C(6)); Y5(Phospho); K11(Label:13C(6))	Y(5): 97.7; T(8): 2.3	Y1469	Y5;	4	1	GPKMKGDyDVTVPKV	2.29		1344.63188	1	0.45

		isoform 1														
ATP1A1	476	sodium/potassium-transporting ATPase subunit alpha-1 isoform a	21361181	GIVVyTGDR	Y5(Phospho); R9(Label:13C(6))	Y(5): 98.7; T(6): 1.3	Y260	Y5;	4	1	TARGIVVyTGDRTVM	2.20	39	1065.50591	0	0.44
AHNAK	79026	neuroblast differentiation-associated protein AHNAK isofor	61743954	VKGEyDMTVPK	K2(Label:13C(6)); Y5(Phospho); K11(Label:13C(6))	Y(5): 99.9; T(8): 0.1	Y964	Y5;	2	1	GPKVKGEyDMTVPKL	2.21		1358.6469	1	0.75

		m 1																
LYN	4067	tyrosine-protein kinase Lyn isoform A	4505055	EEPIyIITEYMAK	Y5(Phospho); K13(Label:13C(6))	Y(5): 99.9; T(8): 0.1; Y(10): 0.0	Y316	Y5;	4	1	VTREEPIyIITEYMA	2.21	50	1685.78276	0	0.41		
G6PD	2539	glucose-6-phosphate 1-dehydrogenase isoform b	108773793	VGFQyEGTYK	Y5(Phospho); K10(Label:13C(6))	Y(5): 99.9; T(8): 0.1; Y(9): 0.0	Y503	Y5;	3	1	MKRVGFQyEGTYKWV	1.78	26	1277.55351	0	2.41		
FRK	2444	tyrosine-protein kinase FRK	4503787	WKLEDYFETDSSySDANNFIR	K2(Label:13C(6)); Y13(Phospho); R21(Label:13C(6))	Y(6): 0.0; T(9): 0.0; S(11): 0.0; S(12): 1.5; Y(13): 97.0; S(14): 1.5	Y497	Y13;	11	1	YFETDSSySDANNFI	4.31	78	2692.16196	1	0.50		

HNRNPA2B1	3181	heterogeneous nuclear ribonucleoproteins A2/B1 isoform B1	14043072	NMGGPYGGGNyGPGGSGGSGGYGGR	Y11(Phospho)	Y(6): 0.1; Y(11): 99.9; S(16): 0.1; S(19): 0.0; Y(22): 0.0	Y336	Y11;	2	1	GPYGGGNyGPGGSGG	5.56	105	2269.86846	0	1.39
CTNND1	1500	catenin delta-1 isoform 2A	332688222	FHPEPyGLEDDQR	Y6(Phospho)	Y(6): 100.0	Y226	Y6;	4	1	HRFHPEPyGLEDDQR	2.51	38	1682.68425	0	1.47
EPB41L1	2036	band 4.1-like protein 1 isoform d	385251401	IRPGEyEQFESTIGFK	Y6(Phospho)	Y(6): 100.0; S(11): 0.0; T(12): 0.0	Y312	Y6;	3	1	IKIRPGEyEQFESTI	2.64		1980.91216	0	6.52
BCAR1	9564	breast cancer anti-estrogen resistance protein 1	282398123	AQQGLyQVPGPSPQFQSPPAK	Y6(Phospho)	Y(6): 100.0; S(12): 0.0; S(17): 0.0	Y126	Y6;	14	1	SKAQQGLyQVPGPSP	4.44	55	2305.11197	0	1.74

		isoform 7														
BCAR1	9564	breast cancer anti-estrogen resistance protein 1 isoform 7	282398123	AQQGLyQVPGPSPQFQSPPAK	Y6(Phospho); K21(Label:13C(6))	Y(6): 100.0; S(12): 0.0; S(17): 0.0	Y126	Y6;	14	1	SKAQQGLyQVPGPSP	4.44	46	2311.12895	0	1.81
PSMA2	5683	proteasome subunit alpha type-2	4506181	HIGLVySGMGPDYR	Y6(Phospho)	Y(6): 100.0; S(7): 0.0; Y(13): 0.0	Y76	Y6;	2	1	TKHIGLVySGMGPDY	4.10	32	1644.72405	0	0.90
PGAM1	5223	phosphoglycerate mutase 1	4505753	LNERHyGGLTGLNK	R4(Label:13C(6)); Y6(Phospho); K14(Label:13C(6))	Y(6): 100.0; T(10): 0.0	Y92	Y6;	3	1	WRLNERHyGGLTGLN	3.49	44	1663.83323	1	0.61
JAK2	3717	tyrosine-protein kinase JAK2	4826776	REVGDyGQLHETEVLLK	Y6(Phospho)	Y(6): 100.0; T(12): 0.0	Y570	Y6;	2	1	VRREVGDyGQLHETE	3.32		2065.99674	1	1.15

ZNF185	7739	zinc finger protein 185 isoform 6	296010968	GALADyEGKDVATR	Y6(Phospho)	Y(6): 100.0; T(13): 0.0	Y349	Y6;	4	1	PKGALADyEGKDVAT	3.85	98	1545.6939	1	1.55
EPHA1	2041	ephrin type-A receptor 1 precursor	221316650	LWLKPyVDLQAYEDPAQGALDFTR	Y6(Phospho)	Y(6): 100.0; Y(12): 0.0; T(23): 0.0	Y599	Y6;	4	1	DKLWLKPyVDLQAYE	4.85	52	2889.39054	0	12.45
BCAR3	8412	breast cancer anti-estrogen resistance protein 3 isoform 1	387157906	CLEEHyGTSPGQAR	C1(Carbamidomethyl); Y6(Phospho)	Y(6): 97.3; T(8): 1.3; S(9): 1.3	Y266	Y6;	9	1	LRCLEEHyGTSPGQA	1.81	32	1684.67681	0	3.12
G6PD	2539	glucose-6-phosphate 1-dehydrogenase isofor	108773793	RVGFQyEGTYK	Y6(Phospho)	Y(6): 98.7; T(9): 1.3; Y(10): 0.0	Y503	Y6;	5	1	MKRVGFQyEGTYKWV	3.70	52	1427.6364	1	1.19

		m b														
YES1	7525	tyrosine-protein kinase Yes	4885661	KLDNGGYyITTR	K1(Label:13C(6)); Y8(Phospho); R12(Label:13C(6))	Y(7): 1.8; Y(8): 98.2; T(10): 0.0; T(11): 0.0	Y223	Y8;	12	1	KLDNGGYyITTRAQF	4.68	63	1492.72356	1	1.25
TES	26136	testin isoform 2	23238188	EGDPAIyAER	Y7(Phospho)	Y(7): 100.0	Y242	Y7;	2	1	KEGDPAIyAERAGYD	1.12	31	1200.49199	0	2.46
ARAP2	116984	arf-GAP with Rho-GAP domain, ANK repeat and PH domain-containing protein 2	21264592	MQDIPIyANVHK	Y7(Phospho)	Y(7): 100.0	Y77	Y7;	2	1	KMQDIPIyANVHKTK	1.81	33	1508.69682	0	1.99

GAB1	2549	GRB2-associated-binding protein 1 isoform b	46370073	GDKQVEyLDLDLDSGK	Y7(Phospho)	Y(7): 100.0; S(14): 0.0	Y627	Y7;	11	1	KGDKQVEyLDLDLDS	4.77	83	1874.84233	1	2.51
GAB1	2549	GRB2-associated-binding protein 1 isoform b	46370073	GDKQVEyLDLDLDSGK	Y7(Phospho)	Y(7): 100.0; S(14): 0.0	Y627	Y7;	11	1	KGDKQVEyLDLDLDS	4.77	49	1874.84067	1	2.39
ANKS1A	23294	ankyrin repeat and SAM domain-containing protein 1A	140161500	EEDEHPyELLLTAETK	Y7(Phospho)	Y(7): 100.0; T(12): 0.0; T(15): 0.0	Y455	Y7;	8	1	REEDEHPyELLLTAE	3.08		1996.87883	0	2.06

S100A10	6281	protein S100-A10	4506761	FAGDKGyLTKEDLR	K5(Label:13C(6)); Y7(Phospho); K10(Label:13C(6)); R14(Label:13C(6))	Y(7): 100.0; T(9): 0.0	Y25	Y7;	4	1	KFAGDKGyLTKEDLR	3.40	83	1710.85991	2	0.09
CD46	4179	membrane cofactor protein isoform 12 precursor	27502427	ADGGAEyATYQTK	Y7(Phospho)	Y(7): 100.0; T(9): 0.0; Y(10): 0.0; T(12): 0.0	Y340	Y7;	10	1	KADGGAEyATYQTKS	2.68	50	1454.58293	0	0.75
YES1	7525	tyrosine-protein kinase Yes	4885661	KLDNGGyYITTR	K1(Label:13C(6)); Y7(Phospho); R12(Label:13C(6))	Y(7): 100.0; Y(8): 0.0; T(10): 0.0; T(11): 0.0	Y222	Y7;	12	1	RKLDNGGyYITTRAQ	4.68	99	1492.72331	1	1.57
ARHGAP35	2909	rho GTPase-activat	150417981	NEEENIySVPHDSTQGK	Y7(Phospho); K17(Label:13C	Y(7): 97.4; S(8): 2.6; S(13): 0.0; T(14): 0.0	Y1105	Y7;	6	1	RNEEENIySVPHDST	4.69	33	2032.85739	0	0.36

		ing protein 35			(6))											
CFL1	1072	cofilin-1	5031635	HELQANCyEEVKDR	C7(Carbamidomethyl); Y8(Phospho); K12(Label:13C(6)); R14(Label:13C(6))	Y(8): 100.0	Y140	Y8;	2	1	HELQANCyEEVKDRC	5.26	65	1882.82187	1	0.74
RPLP0	6175	60S acidic ribosomal protein P0	16933546	IIQLLDDyPK	Y8(Phospho)	Y(8): 100.0	Y24	Y8;	2	1	IIQLLDDyPKCFIVG	2.54	69	1297.64397	0	11.27
ACTN1	87	alpha-actinin-1 isoform c	194097352	HRPELIDyGK	Y8(Phospho)	Y(8): 100.0	Y193	Y8;	2	1	HRPELIDyGKLRKDD	4.06	45	1307.61298	0	1.28
LCK	3932	tyrosine-protein kinase Lck	112789546	NLDNGGFyISPR	Y8(Phospho); R12(Label:13C(6))	Y(8): 100.0; S(10): 0.0	Y192	Y8;	8	1	NLDNGGFyISPRITF	3.29	73	1438.64214	0	0.08

		precursor														
ERBB2IP	55914	protein LAP2 isoform 7	55770895	AQIPEGDyLSYR	Y8(Phospho); R12(Label:13C(6))	Y(8): 100.0; S(10): 0.0; Y(11): 0.0	Y1104	Y8;	5	1	AQIPEGDyLSYREFH	2.87	56	1497.68279	0	0.52
PXN	5829	paxillin isoform 1	170932516	VGEEEHVySFPNK	Y8(Phospho)	Y(8): 100.0; S(9): 0.0	Y118	Y8;	16	1	VGEEEHVySFPNKQK	4.27	49	1614.68401	0	2.73
PXN	5829	paxillin isoform 1	170932516	VGEEEHVySFPNKQK	Y8(Phospho); K13(Label:13C(6)); K15(Label:13C(6))	Y(8): 100.0; S(9): 0.0	Y118	Y8;	26	1	VGEEEHVySFPNKQK	4.68	54	1882.87676	1	2.83
PXN	5829	paxillin isoform 1	170932516	VGEEEHVySFPNK	Y8(Phospho)	Y(8): 98.3; S(9): 1.7	Y118	Y8;	16	1	VGEEEHVySFPNKQK	4.27	40	1614.68369	0	2.95
FAM20B	9917	glycosaminoglycan xylosylkinase	7662150	DHVVEGEPYAGyDR	Y12(Phospho); R14(Label:13C(6))	Y(9): 1.9; Y(12): 98.1	Y141	Y12;	4	1	EGEPYAGyDRHNAEV	1.63	38	1692.70159	0	0.58

LYN	4067	tyrosine-protein kinase Lyn isoform A	4505055	VENCPDELyDIMK	C4(Carbamidomethyl); Y9(Phospho); K13(Label:13C(6))	Y(9): 100.0	Y473	Y9;	2	1	ENCPDELyDIMKMCW	2.09	35	1711.70342	0	0.42
HNRNPH3	3189	heterogeneous nuclear ribonucleoprotein H3 isoform b	14141159	DGMDNQGGyGSVGR	Y9(Phospho)	Y(9): 100.0; S(11): 0.0	Y281	Y9;	4	1	GMDNQGGyGSVGRMG	2.36	60	1492.55242	0	2.69
CALM2	805	calmodulin	4502549	VFDKDGNGyISAAELR	Y9(Phospho)	Y(9): 100.0; S(11): 0.0	Y100	Y9;	14	1	FDKDGNGyISAAELR	4.37	92	1834.83916	1	0.44
CALM2	805	calmodulin	4502549	VFDKDGNGyISAAELR	Y9(Phospho)	Y(9): 100.0; S(11): 0.0	Y100	Y9;	14	1	FDKDGNGyISAAELR	4.37	52	1834.83908	1	0.43

ERBB2IP	55914	protein LAP2 isoform 7	55770895	RAQIPEGDyLSYR	R1(Label:13C(6)); Y9(Phospho); R13(Label:13C(6))	Y(9): 100.0; S(11): 0.0; Y(12): 0.0	Y1104	Y9;	5	1	AQIPEGDyLSYREFH	3.29	65	1659.79302	1	0.60
CALM2	805	calmodulin	4502549	VFDKDGNGyISAAELR	K4(Label:13C(6)); Y9(Phospho); R16(Label:13C(6))	Y(9): 99.9; S(11): 0.1	Y100	Y9;	14	1	FDKDGNGyISAAELR	4.37	55	1846.89079	1	0.41
